# Internet-Delivered Health Interventions That Work: Systematic Review of Meta-Analyses and Evaluation of Website Availability

**DOI:** 10.2196/jmir.7111

**Published:** 2017-03-24

**Authors:** Mary AM Rogers, Kelsey Lemmen, Rachel Kramer, Jason Mann, Vineet Chopra

**Affiliations:** ^1^ Department of Internal Medicine University of Michigan Ann Arbor, MI United States

**Keywords:** Internet, public health, randomized controlled trial, computer-assisted therapy, global health

## Abstract

**Background:**

Due to easy access and low cost, Internet-delivered therapies offer an attractive alternative to improving health. Although numerous websites contain health-related information, finding evidence-based programs (as demonstrated through randomized controlled trials, RCTs) can be challenging. We sought to bridge the divide between the knowledge gained from RCTs and communication of the results by conducting a global systematic review and analyzing the availability of evidence-based Internet health programs.

**Objectives:**

The study aimed to (1) discover the range of health-related topics that are addressed through Internet-delivered interventions, (2) generate a list of current websites used in the trials which demonstrate a health benefit, and (3) identify gaps in the research that may have hindered dissemination. Our focus was on Internet-delivered self-guided health interventions that did not require real-time clinical support.

**Methods:**

A systematic review of meta-analyses was conducted using Preferred Reporting Items for Systematic Reviews and Meta-Analyses (PRISMA) guidelines (PROSPERO Registration Number CRD42016041258). MEDLINE via Ovid, PsycINFO, Embase, Cochrane Database of Systematic Reviews, and the Cumulative Index to Nursing and Allied Health Literature (CINAHL) were searched. Inclusion criteria included (1) meta-analyses of RCTs, (2) at least one Internet-delivered intervention that measured a health-related outcome, and (3) use of at least one self-guided intervention. We excluded group-based therapies. There were no language restrictions.

**Results:**

Of the 363 records identified through the search, 71 meta-analyses met inclusion criteria. Within the 71 meta-analyses, there were 1733 studies that contained 268 unique RCTs which tested self-help interventions. On review of the 268 studies, 21.3% (57/268) had functional websites. These included evidence-based Web programs on substance abuse (alcohol, tobacco, cannabis), mental health (depression, anxiety, post-traumatic stress disorder [PTSD], phobias, panic disorders, obsessive compulsive disorder [OCD]), and on diet and physical activity. There were also evidence-based programs on insomnia, chronic pain, cardiovascular risk, and childhood health problems. These programs tended to be intensive, requiring weeks to months of engagement by the user, often including interaction, personalized and normative feedback, and self-monitoring. English was the most common language, although some were available in Spanish, French, Portuguese, Dutch, German, Norwegian, Finnish, Swedish, and Mandarin. There were several interventions with numbers needed to treat of <5; these included painACTION, Mental Health Online for panic disorders, Deprexis, Triple P Online (TPOL), and U Can POOP Too. Hyperlinks of the sites have been listed.

**Conclusions:**

A wide range of evidence-based Internet programs are currently available for health-related behaviors, as well as disease prevention and treatment. However, the majority of Internet-delivered health interventions found to be efficacious in RCTs do not have websites for general use. Increased efforts to provide mechanisms to host “interventions that work” on the Web and to assist the public in locating these sites are necessary.

## Introduction

### Background

The World Health Organization recognizes that implementation of population-based strategies to improve health is critical [1,2]. Likewise, the Institute of Medicine’s list of suggestions for action includes the implementation of population-based strategies to improve health [3]. The need for population approaches to solve health problems was recently reviewed by David Hunter as he stated, “As countries struggle to transform their health systems to cope with rising demand, aging populations, and largely avoidable lifestyle related illnesses within limited budgets, policy makers are desperate for the right kind of evidence” [4]. With such broad goals in mind, it is surprising that evidence-based mechanisms are not yet fully engaged so that Internet-delivered health interventions can be exploited to achieve these goals.

Although there are numerous websites that contain health-related information, the ability of the consumer—or the patient—to find scientifically robust (ie, evidence-based) health interventions is not fully known. Data from the Pew Research Center indicates that 72% of adults who use the Internet have searched for health-related information in the previous year (based on 2012 survey data) [5]. Furthermore, there is insufficient information to assist the public in deciphering which sites contain useful information that could help them stay healthy, ameliorate risky behaviors, recognize early disease, or assist with treatment of their existing disorders.

The central question is, “Which Internet-delivered health interventions actually work?” For scientists, the answer to this question can be addressed by evaluating the results from randomized controlled trials (RCTs). In fact, the efficacy of some Internet-delivered interventions has already been assessed by investigators. Yet, there is not yet a fully formed mechanism to link these results with the individuals who may wish to use this information.

**Figure 1:**
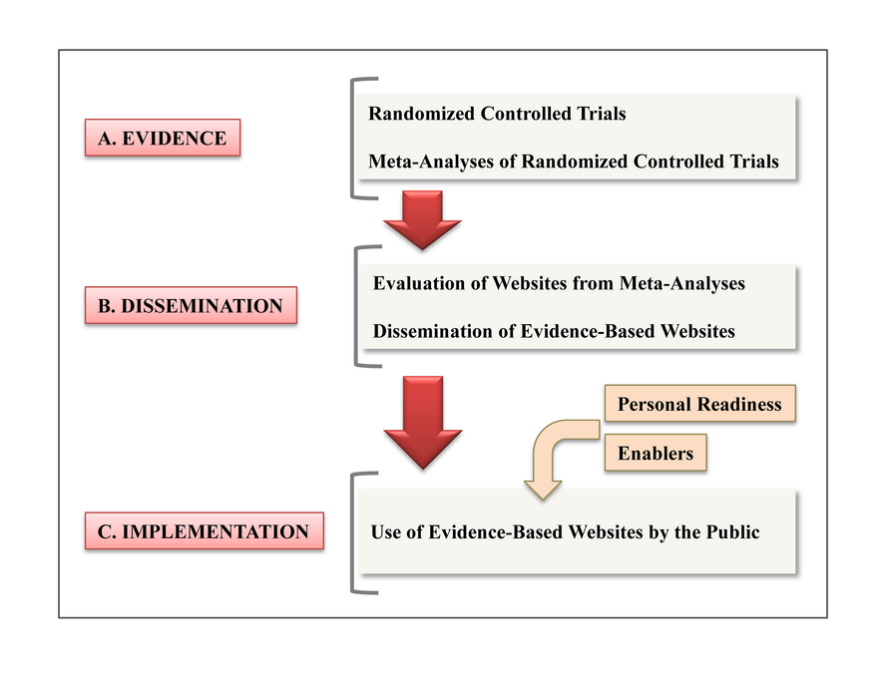
Translating research into implementation for Internet-delivered health interventions.

To expedite this process, there are necessary preparatory steps before implementation. Our translational model is shown in Figure 1 and illustrates the steps. Many RCTs and meta-analyses of RCTs of Internet-delivered health-related interventions are already published and, therefore, some evidence is available. We now, through this report, present the results from the evaluation step of dissemination. That is, we conducted a systematic review of published meta-analyses of RCTs on Internet-delivered health-related interventions. We evaluated this evidence and generated a list of evidence-based websites currently available for use. We were especially interested in Internet-delivered therapies that do not require real-time interaction with a therapist or other health care provider. That is, the application was housed on the Internet for general use by the public.

### Aims of the Study

The aims of this study were to (1) discover the range of health-related topics that were addressed through Internet-delivered interventions, (2) generate a list of current websites used in the trials which demonstrated a health benefit, and (3) identify gaps in the research that may have hindered dissemination.

## Methods

### Inclusion Criteria

The Preferred Reporting Items for Systematic Reviews and Meta-Analyses (PRISMA) guidelines were used [6]. To be eligible for inclusion, studies were required to be meta-analyses of RCTs with at least one intervention that was Internet-delivered and reported a health-related outcome. Within each meta-analysis, we required that there be at least one self-guided intervention without therapist or clinician support. For this review, we excluded group-based interventions (ie, trials that enrolled groups of people to experience the intervention together). There were no restrictions on the types of individuals in the trials or the type of health outcomes.

### Search Strategy

A comprehensive search strategy was developed with a biomedical research librarian and was undertaken to identify articles for inclusion. The following electronic databases were searched: MEDLINE via Ovid, PsycINFO, Embase, Cochrane Database of Systematic Reviews, and the Cumulative Index to Nursing and Allied Health Literature (CINAHL). Search strategies utilized a combination of keywords and MeSH headings (Table 1). The last date of the search was June 14, 2016. There were no language restrictions on the search.

**Table 1:** Search strategy for the systematic review.

Database	Search terms
MEDLINE via Ovid	1. (internet or web or “web-delivered” or online).ti.
	2. (“meta-analysis” or “metaanalysis” or “meta-analytic”).ab,ti.
	3. Random*.ab,ti.
	4. 1 AND 2 AND 3
PsycINFO	1. AB (web OR internet OR web-based OR online OR web-delivered)
	2. AB (meta-analysis or meta-analytic or meta-analysis)
	3. AB random*
	4. 1 AND 2 AND 3
	5. Restrict 4 to “meta-analysis”
Embase	1. (web:ti OR internet:ti OR online:ti OR ‘web based’:ti OR ‘web delivered’:ti)
	2. ‘meta analysis’:ab,ti
	3. Random*ab,ti
	4. 1 AND 2 AND 3
Cochrane Database of Systematic Reviews	1. web OR internet OR online OR “web-based” OR “web-delivered”(record title)
CINAHL (Cumulative Index to Nursing and Allied Health Literature )	1. TI (web OR internet OR web-based OR online OR web-delivered)
	2. TI (meta-analysis OR metaanalysis OR meta-analytic)
	3. TI (random*)
	4. 1 AND 2 AND 3

### Screening of Articles

Three authors (KL, RK, and MR) independently reviewed the title and abstract of each record to determine eligibility. Any disagreements regarding inclusion or exclusion were resolved by a discussion between two authors (MR and KL). Full papers of the selected title and abstracts were reviewed independently by three authors (KL, RK, and MR) and disagreements regarding inclusion or exclusion were resolved by a discussion between two authors (KL and MR). All RCTs within each meta-analysis were screened for eligibility (self-guided Internet-based health-related intervention).

### Analyses

The purpose of the analyses was to combine the results from across all the meta-analyses so that the results could be summarized and the Internet programs could be located. Individual RCTs within each meta-analysis were grouped by topic: Substance Abuse, Mental Health, Diet and Physical Activity, Disease Management, Disease Prevention, and Childhood Health Problems. A health benefit was defined as a statistically significant improvement in any health-related outcome within an RCT; all trials assessed outcomes through inferential statistics with alpha set at .05, 2-tailed. The concurrent control groups did not receive the Internet-delivered intervention (generally a wait list) unless specifically stated. Measures of efficacy were calculated when data were available within each RCT; for binary outcomes, number needed to treat (NNT) was calculated when absolute measures were reported. For outcomes measured using continuous scales, mean changes were listed (intervention relative to control).

Each RCT was reviewed for the name of the intervention and the website that housed the intervention. Functional websites of such evidence-based interventions (demonstrating a health benefit) were located on November 18, 2016. We defined functional website as those sites which housed the program which was tested in the RCTs and was available for general use.

## Results

**Figure 2:**
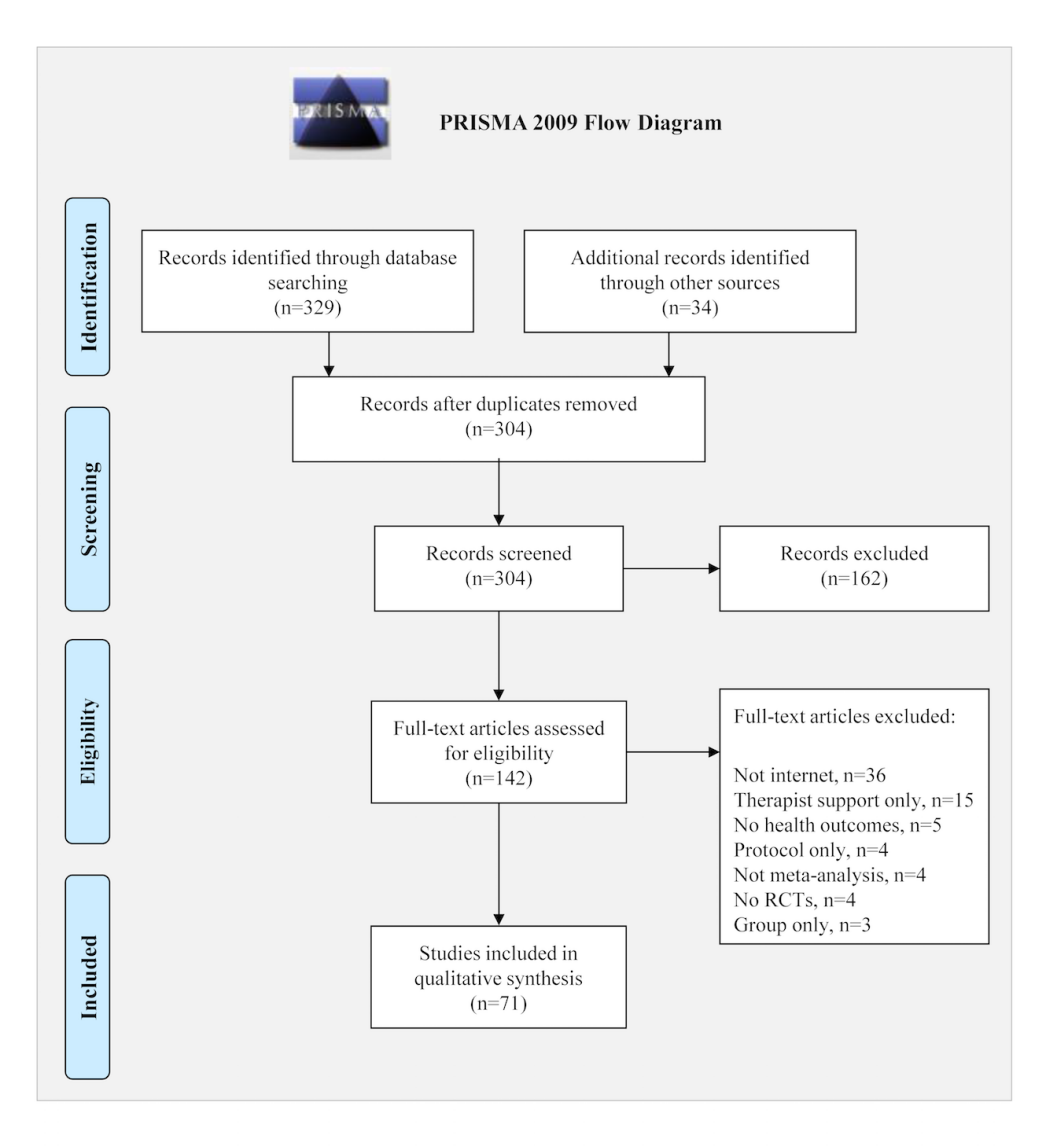
The Preferred Reporting Items for Systematic Reviews and Meta-Analyses (PRISMA) flow diagram.

There were 363 records identified through the search (Figure 2) which yielded 304 records after removing the duplicates. The abstracts were reviewed and 162 were excluded because they did not meet the eligibility criteria. Full-text articles were reviewed for the 142 remaining articles and 36 were excluded due to noninvolvement of the Internet, 15 were excluded due to therapist or clinician support only, and 20 were excluded due to either protocol only, no health outcomes, group-based interventions only, not a meta-analysis, or did not include RCTs. There were 71 meta-analyses of Internet-based interventions that met eligibility criteria and were included in this study [7-76].

Within the 71 meta-analyses, there were 1733 studies. Of these studies, there were 268 unique RCTs that were self-help Internet-based interventions; and of the 268 studies, there were 57 trials demonstrating a health benefit with a functional website [77-138]. The topics covered are listed in Table 2.

**Table 2:** Number of randomized controlled trials with functional websites of self-help Internet-delivered health interventions.

Category	Topic	Number of RCTs reviewed	Number of RCTs with websites^a^	RCTs with websites^a^ (%)
**Substance abuse**				
	Alcohol	72	8	11
	Smoking (tobacco)	30	7	23
	Cannabis	3	2	67
	Drug use (general)	1	0	0
	Total	106	17	16.0
**Mental health**				
	Depression	15	4	27
	Anxiety	16	5	31
	Post-traumatic stress disorder	11	2	18
	Phobias	9	3	33
	Panic disorders	5	2	40
	Obsessive compulsive disorder	1	1	100
	Mental health (general)	3	0	0
	Eating disorders	4	0	0
	Infertility distress	2	0	0
	Total	66	17	26
**Diet and physical activity**				
	Diet	13	7	54
	Physical activity	33	6	18
	Total	46	13	28
**Disease management**				
	Insomnia	13	4	31
	Chronic pain	10	2	20
	Diabetes	5	0	0
	Fatigue	1	0	0
	Tinnitus	2	0	0
	Total	31	6	19
**Disease prevention**				
	Cardiovascular risk	9	2	22
	Cancer prevention (skin)	1	0	0
	Sexual health	7	0	0
	Total	17	2	12
**Childhood health problem**s				
	Childhood behavior problems	1	1	100
	Encopresis	1	1	100
	Total	2	2	100
	Grand total	268	57	21.3

^a^Websites in which there was a health benefit demonstrated in an RCT (randomized controlled trial).

Internet self-help for substance abuse was the most frequent topic in RCTs, with alcohol having the greatest number of trials. Of the 72 trials on alcohol use, there were 8 with functioning websites. Tobacco use was also a common subject for interventions with 7 websites (from the 30 trials reviewed). Mental health interventions were available, including anxiety (5 websites on generalized anxiety disorder, 3 on phobias, 2 on panic disorders, 2 on post-traumatic distress disorder, and 1 on obsessive compulsive disorder [OCD]) and depression (4 websites). There were 46 RCTs reviewed for diet and physical activity interventions, and 13 of those yielded a functioning website. There were fewer RCTs on disease management, with insomnia and chronic pain yielding 13 and 10 trials, respectively. Within the meta-analyses, there were a few RCTs specifically on cardiovascular risk factors (blood pressure, cholesterol, and hyperlipidemia) and several on sexual health (sexually transmitted disease, sexual dysfunction, unintended pregnancy). However, there was only one RCT on self-help regarding cancer prevention (for skin cancer). Finally, there were two trials targeted to parents of children with health problems—one on behavioral problems and one on encopresis.

In most instances, the Internet-delivered interventions were offered only to the study participants in the context of the RCT; websites to deliver the intervention after the conclusion of the study were not available. For example, *Student Bodies* was an efficacious Internet-delivered program for eating disorders in girls but the program was not available for general use [139-141]. In the area of sexual health, there were several efficacious Internet-delivered programs regarding sexually transmitted diseases [142-144]. However, these sites were only available for study participants during the course of the research study. Overall, in only 21.3% (57/268) of instances, there was a functional website for the interventions after the conclusion of the trial.

We compiled a list of websites of the Internet-delivered interventions providing a health benefit and these are shown in Table 3 with the name of the program, hyperlink to the site, cost, and the languages utilized for delivery of the program.

**Table 3:** Evidence-based websites of Internet-delivered health-related interventions.

	Target population	Name	Websites	Cost	Language
**Alcohol**					
	Adult drinkers	Check Your Drinking	http://www.checkyourdrinking.net/CYD/CYDScreenerP1_0.aspx	Free	English, French, Portuguese, Spanish
	Male adults	Drinktest.nl	http://www.drinktest.nl/	Free	Dutch
	Adult at-risk drinkers	Balance	http://akan.no/verktoy/balance/	Free	Norwegian
	Adult problem drinkers	Drinking Less	http://minderdrinken.nl/	Free	Dutch
	Universities	Alcohol eCHECKUP TO GO (eCHUG)	http://www.echeckuptogo.com/programs/alcohol	Commercial	English
	Universities	Alcohol Edu	https://everfi.com/health-wellness/	Commercial	English
	University students	MyStudentBody	https://www.mystudentbody.com	Commercial	English
	University students	Alcohol-Wise	https://web.3rdmilclassrooms.com/courses/college/alcohol-wise	Commercial	English
**Tobacco**					
	Smokers	Smokefree	https://smokefree.gov/	Free	English, Spanish
	Smokers	QuitCoach	http://www.quitcoach.org.au/	Free	English
	Smokers	Stop-tobacco	http://www.stop-tobacco.ch/en/	Free	French, German, Italian, English, Spanish, Portuguese
	Smokers	Dejar de Fumar (Give Up Smoking)	http://www.apsiol.uned.es/dejardefumar/	Free	Spanish
	Smokers	Guía para dejar de fumar (Guide to Quitting Smoking)	https://www.aecc.es/Comunicacion/publicaciones/Documents/Guia_dejar_fumar.pdf	Free	Spanish
	Smokers	Slutta (Quit)	https://helsenorge.no/rus-og-avhengighet/snus-og-roykeslutt/	Free	Norwegian
	Smokers	QuitNet	https://quitnet.meyouhealth.com/#/	Some features free, some require a fee	English
**Cannabis**					
	Cannabis users	Reduce Your Use: How to Break the Cannabis Habit	https://reduceyouruse.org.au/sign-up/	Free	English
	Cannabis users	Quit the Shit	www.drugcom.de/?id=quittheshit	Free	German
**Depression**					
	Individuals with depression	MoodGYM	https://moodgym.anu.edu.au/welcome	Free	English, Finnish, Norwegian, Dutch
	Individuals with depression	BluePages	http://bluepages.anu.edu.au/	Free	English, Norwegian
	Individuals with depression	Deprexis	http://www.deprexis.com/	Commercial	English, German
	Individuals with depression	Kleur Je Leven (Color Your Life)	http://www.kleurjeleven.nl/	Commercial	Dutch
**Generalized anxiety disorder**					
	Individuals with anxiety	Mental Health Online	https://www.mentalhealthonline.org.au/Default.aspx	Free	English
	Individuals with anxiety	This Way Up	https://thiswayup.org.au/	Some free, some commercial	English
	Workers	Stress and Mood Management	http://centerforworkforcehealth.com/programs/stress-and-mood-management/	Commercial	English
	Colleges, universities	My Student Body—Stress	https://www.mystudentbody.com/	Commercial	English
	Individuals with anxiety	Internet-based Mindfulness Treatment	https://www.mindfulnesscenter.se/en	Commercial	Swedish, English, Norwegian
**Post-traumatic stress disorder**					
	Individuals who experienced trauma	My Trauma Recovery	http://mytraumarecovery.com/	Free	English
	Individuals who experienced disasters	My Disaster Recovery	http://disaster.bluesunsupport.com/	Free	English, Spanish, Mandarin
**Panic disorder and phobias**					
	Individuals with panic disorders or Phobias	Mental Health Online	https://www.mentalhealthonline.org.au/Default.aspx	Free	English
	Individuals with panic disorders or phobias	This Way Up	https://thiswayup.org.au/	Some free, some commercial	English
	Adults with glossophobia (fear of public speaking)	Talk to Me	http://www.internetmeayuda.com/mhpEnglish/saludo.htm	Free	Spanish, English
**Obsessive compulsive disorder**					
	Individuals with OCD^a^	This Way Up	https://thiswayup.org.au/	Some free, some commercial	English
**Diet and physical activity**					
	Adults	Gezond Leven Check (Healthy Living Check)	http://www.gezondlevencheck.nl/	Free	Dutch
	Sedentary overweight adults	Active Living Every Day (ALED-I)	http://www.activeliving.info/demo/demo_osg_welcome.cfm	Free (fee for extra materials)	English
	Adults with diabetes	My Path to Healthy Life	http://mypathtohealthylife.com/	Free	English, Spanish
	Adults in workplace	Food Smart	http://centerforworkforcehealth.com/index.cfm/programs/food-smart/	Commercial	English
	Overweight and obese adults	The Biggest Loser Club	https://www.biggestloserclub.com/	Commercial	English
	University students	My Student Body—Nutrition	https://www.mystudentbody.com/	Commercial	English
	Managers of organizations	ExecuPrev	https://www.execuprev.com	Commercial	English
	Employees in workforce	DASH^b^ for Health	http://www.dashforhealth.com/	Commercial	English
**Insomnia**					
	Adults with chronic insomnia	Insomnie	http://www.insomnie.nl/	Free	Dutch
	Adults with insomnia	SHUTi	http://www.myshuti.com/	Commercial	English
	Individuals with sleep problems	Sleepio	https://www.sleepio.com/	Commercial	English
	Adults with chronic insomnia	**REST**ORE	http://cobalttx.com/Products/restore.html	Commercial	English
**Chronic pain**					
	Adults with chronic pain	painACTION	http://www.painaction.com/#	Free	English
	Adults with chronic pain	Chronic Pain Management Program	https://pain.goalistics.com/	Commercial	English
**Hypertension and hyperlipidemia**					
	Adults with hypertension	Blood Pressure Action Plan	http://www.heartandstroke.on.ca/site/c.pvI3IeNWJwE/b.3582093/k.8AB3/Blood_Pressure_Action_Plan.htm	Free	English
	Employees in workforce	DASH^b^ for Health	http://www.dashforhealth.com/	Commercial	English
**Childhood health problems**					
	Parents with children who have behavior problems	Triple P Online	http://www.triplep-parenting.com	Commercial	English, Spanish, Dutch, German
	Parents of children with encopresis	U Can POOP Too	http://www.ucanpooptoo.com/	Free	English

^a^OCD: obsessive compulsive disorder.

^b^DASH: Dietary Approaches to Stop Hypertension.

### Substance Abuse

From this review, we found that there are eight currently available evidence-based websites on alcohol use. All but three were conducted using college or university students; some are specifically targeted to universities, offering a suite of programs regarding substance abuse, health, and wellness. The most common techniques utilized in these interventions (Table 4) were personalized and normative feedback, as well as goal setting. Some sites included more tailored feedback and interactive journaling. The health benefit observed in the trials was generally a reduction in alcohol consumption, although some trials showed a reduction in the consequences of heavy drinking such as impairment in control and fewer embarrassing actions. The length of the programs varied—some being rather brief screening tools and others encompassing 6 months of structured activities [77-91]. The freely available websites for alcohol emanated from various European countries. Screen shots of each home page of the websites are given in Multimedia Appendix 1.

For tobacco use, there are several free evidence-based websites for reducing or quitting smoking. Four of these sites were available in English, 4 in Spanish, and 1 in Norwegian; the *Stop-tobacco* program was available in multiple languages. Often they were supported with governmental or public health funding such as *smokefree* in the United States, *Stop-tobacco* in Switzerland, and *Quit* in Norway. In general, the primary outcome was greater abstinence rates of smoking which were achieved through structured cognitive behavioral techniques, including motivational materials, personalized and tailored advice, goal setting, feedback mechanisms, and self-monitoring.

There were a few RCTs on curbing cannabis use which reported a reduction in the frequency or quantity of use. The mechanisms used to achieve the health benefits were similar to those used for tobacco use, relying heavily on cognitive behavioral approaches (listed in Table 4). One free Internet-delivered intervention *Reduce Your Use* comes from Australia and another was developed in Germany (*Quit the Shit*).

**Table 4:** Health benefits of evidence-based websites of Internet-delivered health-related interventions.

	Name		Website		Intervention techniques		Health benefits	
**Alcohol**							
	Check Your Drinking		http://www.checkyourdrinking.net/CYD/CYDScreenerP1_0.aspx		Personalized and normative feedback		Reduction in weekly alcohol consumption
	Drinktest.nl		http://www.drinktest.nl/		Personalized and normative feedback		Reduction in alcohol consumption
	Balance		http://akan.no/verktoy/balance/		Personalized and normative feedback		Reduction in alcohol consumption
	Drinking Less		http://minderdrinken.nl/		Goal setting, analysis of drinking behavior, maintenance, and relapse prevention		Reduction in alcohol consumption
	Alcohol eCHECKUP TO GO (eCHUG)		http://www.echeckuptogo.com/programs/alcohol		Individualized feedback, recognition of harms		Reduction in alcohol consumption
	Alcohol Edu		https://everfi.com/health-wellness/		Cognitive-behavioral skills, normative drinking, motivational information		Reduction in alcohol consumption, reduction in heavy alcohol use, reduction in consequences (eg, embarrassing actions, impaired control)
	MyStudentBody		https://www.mystudentbody.com		Tailored feedback, normative feedback, educational tools on behavior and consequences		Reduction in binge drinking, reduction in alcohol consumption among persistent heavy drinkers
	Alcohol-Wise		https://web.3rdmilclassrooms.com/courses/college/alcohol-wise		Information on social norms, interactive journaling, educational feedback		Reduction in peak number of drinks and blood alcohol concentration
**Tobacco**							
	Smokefree		https://smokefree.gov/		Motivational materials, step-by-step quitting guide, task charts, self-monitoring tools, personal calendar		Greater abstinence rates of smoking
	QuitCoach		http://www.quitcoach.org.au/		Personalized, tailored advice. Feedback with suggestions and encouragement.		Greater abstinence rates of smoking
	Stop-tobacco		http://www.stop-tobacco.ch/en/		Tailoring with stages of change, coping methods, self-change strategies, feedback		Greater abstinence rates of smoking
	Dejar de Fumar (Give Up Smoking)		http://www.apsiol.uned.es/dejardefumar/		Education, self-monitoring, self-control, relapse prevention, coping skills, lifestyle change		Greater abstinence rates of smoking in program completers, decrease in number of cigarettes in smokers
	Guía para dejar de fumar (Guide to Quitting Smoking)		https://www.aecc.es/Comunicacion/publicaciones/Documents/Guia_dejar_fumar.pdf		Noninteractive smoking cessation guide, cigarette counter, online journal		Increase in cigarette quit rates
	Slutta (Quit)		https://helsenorge.no/rus-og-avhengighet/snus-og-roykeslutt/		Personalized, adaptive messages with feedback. Coordinated with steps in behavioral change.		Greater short-term abstinence rates of smoking
	QuitNet		https://quitnet.meyouhealth.com/#/		Advice, setting quit date, individually tailored feedback, problem solving skills, support		Increase in abstinence rates of smoking
**Cannabis**							
	Reduce Your Use: How to Break the Cannabis Habit		https://reduceyouruse.org.au/sign-up/		Tracking use, information regarding attitudes, goal setting, expenditures, motivational feedback		Reduction in days of use and quantity of use
	Quit the Shit		www.drugcom.de/?id=quittheshit		Personalized feedback, goal setting, information on strategies		Reduction in frequency of use and quantity of use
**Depression**							
	MoodGYM		https://moodgym.anu.edu.au/welcome		Cognitive behavioral therapy		Reduction in symptoms of depression
	BluePages		http://bluepages.anu.edu.au/		Information on depression, symptoms, prevention, and treatments, sources of help (used in conjunction with MoodGYM)		Reduction in symptoms of depression
	Deprexis		http://www.deprexis.com/		Cognitive behavioral therapy		Reduction in symptoms of depression
	Kleur Je Leven (Color Your Life)		http://www.kleurjeleven.nl/		Cognitive behavioral therapy		Improvement in short-term depressive symptoms in frequent users of the site
**Generalized anxiety disorder**							
	Mental Health Online		https://www.mentalhealthonline.org.au/Default.aspx		Cognitive behavioral therapy		Reduction in general anxiety levels
	This Way Up		https://thiswayup.org.au/		Cognitive behavioral therapy		Reduction in anxiety in those who completed the program
	Stress and Mood Management		http://centerforworkforcehealth.com/programs/stress-and-mood-management/		Cognitive behavioral therapy		Reduction in stress
	My Student Body—Stress		https://www.mystudentbody.com/		Cognitive behavioral therapy		Decreased anxiety and family problems
	Internet-based Mindfulness Treatment		https://www.mindfulnesscenter.se/en		Mindfulness		Decreased anxiety, severity of insomnia, and depression. Increased quality of life.
**Post-traumatic stress disorder**							
	My Trauma Recovery		http://mytraumarecovery.com/		Interactive modules: seeking help, relaxation, social support, coping, self-talk, triggers, and memories		Reduction in post-traumatic symptom severity
	My Disaster Recovery		http://disaster.bluesunsupport.com/		Interactive modules: seeking help, relaxation, social support, coping, self-talk, triggers, and memories		Decreased worry
**Panic disorder and phobias**							
	Mental Health Online		https://www.mentalhealthonline.org.au/Default.aspx		Cognitive behavioral therapy		Reduction in frequency of panic and anticipatory fear of panic
	This Way Up		https://thiswayup.org.au/		Cognitive behavioral therapy		Reduction in social phobia in those who completed the program
	Talk to Me		http://www.internetmeayuda.com/mhpEnglish/saludo.htm		Cognitive behavioral therapy		Decrease in fear and avoidance of public speaking
**Obsessive compulsive disorder**							
	This Way Up		https://thiswayup.org.au/		Cognitive behavioral therapy		Reduction in symptoms of OCD^a^, distress and depression
**Diet and physical activity**							
	Gezond Leven Check (Healthy Living Check)		http://www.gezondlevencheck.nl/		Precaution adoption process model including tailored, personalized feedback, normative behaviors, and suggestions		Reduction in saturated fat intake and increase in physical activity
	Active Living Every Day (ALED-I)		http://www.activeliving.info/demo/demo_osg_welcome.cfm		Self-paced program, interactive activities, behavior modification strategies		Increased daily steps in people with low baseline activity. Reduction in waist circumference.
	My Path to Healthy Life		http://mypathtohealthylife.com/		Goal setting, tracking progress, feedback, resources, interaction, motivational tips		Increase in healthy eating habits and physical activity. Decrease in fat intake.
	Food Smart		http://centerforworkforcehealth.com/index.cfm/programs/food-smart/		Information on diet, stress, fitness or physical activity. Self-tailored content. Interactive activities.		Improvement in dietary self-efficacy, dietary attitudes, and dietary stage of change
	The Biggest Loser Club		https://www.biggestloserclub.com/		Social cognitive theory. Self-efficacy, goal setting, self-monitoring, outcome expectations, interaction.		Reduction in body mass index, weight, waist circumference, and waist-to-height ratio
	My Student Body— Nutrition		https://www.mystudentbody.com/		Information targeted to students, rate myself assessment with feedback, diet and physical activity information, resources		Increase in fruit and vegetable intake
	ExecuPrev		https://www.execuprev.com		Animated and interactive learning on health and executive leadership, self-assessment, simulations, advice from experts, coaching and webinars		Improvement in dietary self-efficacy and dietary attitudes. Decrease in waist circumference in women.
	DASH^b^ for Health		http://www.dashforhealth.com/		Advice on nutrition and physical activity. Feedback on 24-h food recall, weight, blood pressure. Progress reports.		Decrease in weight for obese or overweight individuals. Increased intake of fruits and vegetables. Lowered consumption of carbonated beverages.
**Insomnia**							
	Insomnie		http://www.insomnie.nl/		Cognitive behavioral therapy		Improvement in sleep efficiency, total sleep time, sleep onset latency, wake after sleep onset and number of nocturnal awakenings. Decrease in anxiety and depression.
	SHUTi		http://www.myshuti.com/		Cognitive behavioral therapy		Improvement in sleep efficiency, insomnia severity, sleep onset latency, soundness of sleep, restored feeling on awakening, and general fatigue
	Sleepio		https://www.sleepio.com/		Cognitive behavioral therapy		Improvement in sleep efficiency, total sleep time, sleep onset latency, wake after sleep onset, sleep quality, and daytime functioning
	**REST**ORE		http://cobalttx.com/Products/restore.html		Cognitive behavioral therapy		Improvement in sleep quality, insomnia severity, and daytime fatigue
**Chronic pain**							
	painACTION		http://www.painaction.com/#		Self-management strategies and cognitive behavioral therapy		Reduction in pain intensity, depression, anxiety, stress. Increased improvement in pain, coping, use of social support.
	Chronic Pain Management Program		https://pain.goalistics.com/		Self-directed interactive learning, integrating social networking, and self-management tools		Reduction in pain severity, pain-related interference, emotional burden, perceived disability, catastrophizing, and pain-induced fear. Decreased depression, anxiety, and stress.
**Hypertension and hyperlipidemia**							
	Blood Pressure Action Plan		http://www.heartandstroke.on.ca/site/c.pvI3IeNWJwE/b.3582093/k.8AB3/Blood_Pressure_Action_Plan.htm		Stages of readiness, setting priorities, motivation, tailored advice, self-direction		Decrease in systolic blood pressure and total cholesterol in persons completing the program
	DASH for Health		http://www.dashforhealth.com/		Advice on nutrition and physical activity. Feedback on 24-h food recall, weight, blood pressure. Progress reports.		Decrease in systolic blood pressure in individuals with hypertension


**Childhood health problems**							
	Triple P Online		http://www.triplep-parenting.com		Interactive, self-directed positive parenting skills. Goal setting, evaluation, self-efficacy, and personal agency skills. Video-based modeling, experiential learning, prompting. Customizable output. Cultural sensitivity.		Reduction in the frequency and intensity of child behavioral and emotional problems. Increase in child adjustment. Decrease in dysfunctional parenting styles and parental anger. Increase in parents’ confidence.
	U Can POOP Too		http://www.ucanpooptoo.com/		Behavioral approach with reinforcement for spontaneous toilet use and clean pants, instructions and modeling of behaviors and actions, education		Reduced fecal soiling, increased defecation in the toilet, and increased unprompted trips to the toilet

^a^OCD: obsessive compulsive disorder.

^b^DASH: Dietary Approaches to Stop Hypertension.

### Mental Health

There were 4 functional websites that were intended to help individuals with depression. *MoodGYM* and *BluePages* are generally used together, the first for the delivery of cognitive behavioral therapy and the following as an adjunct. *MoodGYM* was more effective when the entire program was completed—not brief interventions [145]. *MoodGYM* contains five sequential modules which are completed at the pace of each user. There were a few commercial sites as well— *Deprexis* (9-week program) and *Color Your Life* (8 weeks with a 9th-week booster). Both of these programs led to a reduction in the symptoms of depression. Cognitive behavioral therapy was the mechanism utilized in each of these interventions which was delivered in a modular, stepwise manner over several months. It is important to note that one of the Internet-delivered programs for treating insomnia (*Insomnie*) also led to a decrease in depression and anxiety.

There were several websites that addressed generalized anxiety, most delivering cognitive behavioral therapy and one delivering mindfulness therapy. *Mental Health Online* and *This Way Up* both emanate from Australia. *Stress and Mood Management* is from the Center for Workforce Health and is a commercial program targeting workers. On the site, a suite of programs is offered on various health-related topics. *My Student Body*, likewise, offers a suite of programs, one being on *Stress* targeted to colleges and universities. Cognitive behavioral therapy was the mechanism used in all of the anxiety interventions except for one which used mindfulness. Mindfulness treatment was available in several languages which reduced anxiety, depression, and the severity of insomnia.

Post-traumatic stress disorder (PTSD) was addressed at two partner websites—one focusing on various trauma recoveries and the other directed to individuals who experienced disasters. It was tested in an RCT for hurricane survivors [112] and is available in English, Spanish, and Mandarin. The Internet sites for PTSD used various coping strategies and behaviors which were based on social cognitive theory. The modules included social support, self-talk, relaxation, trauma triggers, unhelpful coping, and professional help.

There are functional websites for the treatment of specific phobias and panic disorders as well. Both *Mental Health Online* and *This Way Up* address these disorders through cognitive behavioral therapy. In addition, *This Way Up* offers a 10-week program for individuals with OCD which was effective in reducing the symptoms of OCD, as well as reducing distress and depression in those with OCD.

*Talk to Me* was developed by Spanish psychologists to treat fear of public speaking and is available for use. This 2-month program using cognitive behavioral techniques was effective in decreasing the fear and avoidance of public speaking. This same group developed *Without Fear* which is an Internet-delivered program for fear of small animals (spiders, cockroaches, mice).

### Diet and Physical Activity

There were several websites on diet and physical activity interventions. In general, these programs included interactive components with goal setting and personalized feedback. Often self-monitoring and tracking of progress were included. Some of the commercial websites were found to be efficacious in terms of reducing body mass index or weight. *The Biggest Loser Club* was efficacious (decreased weight, body mass index, waist circumference) with a 12-week program [123]. Additional support (periodic reminders) did not improve the basic Internet program [123].

*Dietary Approaches to Stop Hypertension (DASH) for Health* is another evidence-based program with a focus on diet and physical activity. For those who completed 12 months of use, overweight or obese individuals lost weight (mean decrease of 4 pounds) [126]. Overall, people with hypertension lowered their systolic blood pressure by an average of 7 mmHg [126]. It also led to increased consumption of fruits and vegetables and lower consumption of carbonated beverages. The program included weekly education, motivation, and mechanisms for self-monitoring with progress reports [126].

The Center for Workforce Health includes a suite of programs, some of which specifically address diet and physical activity. The RCTs captured in this review indicated that the two modules entitled *Stress and Mood Management* and *Food Smart* showed health benefits. There was a reduction in stress after the completion of *Stress and Mood Management* (3-month program), and after the *Food Smart* program, there was improvement in dietary self-efficacy, dietary attitudes, and dietary stage of change [108,122].

*My Student Body* also contains a suite of Internet programs. Our review indicated that the packages for *Nutrition*, *Alcohol* use, and *Stress* were efficacious in various RCTs. These are now combined and sold commercially, generally to colleges and universities. *My Student Body—Nutrition* specifically increased the intake of fruit and vegetables.

*Healthy Living Check*, *Active Living Every Day*, and *My Path to Healthy Life* addressed both diet and physical activity. Completion of these programs led to various health benefits including a reduction in the intake of saturated fat, reduction in waist circumference, and an increase in physical activity. *My Path to Healthy Life* was targeted to adults with diabetes mellitus [121], and completion of this program led to a decrease in fat intake and an increase in physical activity.

Some health interventions were paired with other interventions. *ExecuPrev* (*LeadWell LiveWell*) paired a leadership intervention with cardiovascular disease prevention. This program decreased waist circumference in women, although it did not affect body mass index overall [125].

### Disease Management

All of the meta-analyses of RCTs on insomnia indicated that Internet-based cognitive behavioral therapy for insomnia was efficacious. In general, the therapy was delivered over several months through a series of modules and included self-monitoring through sleep diaries. The content of the therapy often included sleep information, sleep hygiene, relaxation, stimulus control, sleep restriction, and various cognitive techniques such as restructuring, paradox, mindfulness, imagery, putting day to rest, and thought stopping. Only some of the applications, however, are currently available. *Insomnie* was developed in the Netherlands and is available in Dutch. *SHUTi*, *Sleepio*, and *RESTORE* are available commercially in English. Generally, completion of these programs takes weeks to months, with specified activities required during each step of the program. The main health benefits were improvement in sleep efficiency and sleep quality, with a decrease in the severity of insomnia.

There were two evidence-based sites for chronic pain: *painACTION* and the *Chronic Pain Management Program*. The free site *painACTION* offers programs in back pain, migraines, neuropathic pain, and pain due to cancer or arthritis. *painACTION* is a 4-week course followed by 5 monthly boosters and includes self-management education in which problem solving skills were taught to reach specific goals [133,134]. The 6-week *Chronic Pain Management Program* covered four domains: cognitive (thinking better), behavioral (doing more), social (relating better), and emotional (feeling better). Both pain-related sites utilize various cognitive behavioral approaches with self-management strategies and interactive elements. These programs led to a reduction in the intensity or severity of pain, as well as a reduction in stress, anxiety, and depression.

### Disease Prevention

The *Blood Pressure Action Plan* (now called the *Heart and Stroke Foundation Health e-Support* program) resulted in lower systolic blood pressure, lower pulse pressure, and lower total cholesterol in those individuals who completed the 4-month program [136]. *DASH for Health* also lowered systolic blood pressure [126]. These programs involved setting priorities and included self-monitoring, progress reports, and tailored advice.

### Childhood Health Problems

There were two efficacious programs on childhood health problems. *Triple P Online* (*TPOL*) assists parents in addressing behavioral programs in children through teaching positive parenting skills. It was shown to decrease problematic child behavior, dysfunctional parenting styles, parental anger, and to improve parent’s confidence. It has been studied quite extensively and is used in 25 countries throughout the world (with availability in English, Spanish, Dutch, and German). The techniques used included goal setting, evaluation, self-efficacy, personal agency skills, with video-based modeling, experiential learning, prompting, and customizable output. The other site for childhood problems is entitled *U Can POOP Too* which addresses encopresis. It has been shown to reduce fecal soiling and improve toileting skills through various cognitive behavioral approaches including reinforcement and modeling of behaviors and actions.

### Measures of Efficacy

The principal measure of efficacy was NNT; these were calculated for binary outcomes and are shown in Table 5. For continuous outcomes, changes in mean differences between the intervention and control groups are shown. Overall for substance abuse (alcohol, tobacco, and cannabis), the effect was moderate with NNTs of 9-26 for avoidance or reduction in use over a short-term period (up to 6 months). For mental health problems, the degrees of effect were commonly reported using conventional scales within each field. In general, the effects were moderate with a decrease in depressive symptoms, anxiety, or stress. There were two interventions (Deprexis and Mental Health Online for panic disorders) which demonstrated particular efficacy (ie, low NNTs of 4 and 2, respectively). The efficacy of the interventions for diet and physical activity, although significant, was modest (eg, 2.1 kg mean weight reduction compared with a 0.4 kg increase in controls). There were several efficacious interventions for insomnia; the severity of symptoms, in general, decreased moderately. For example, SHUTi showed an 8-point relative reduction in severity on the Insomnia Severity Index. In addition, Sleepio also demonstrated an increase in daytime performance (2.5 points on a 5-point scale). The intervention painACTION was particularly efficacious, with an NNT of 4 for back pain and a NNT of 3 for migraine headaches. The interventions targeted to parents of small children were also very efficacious. Triple P Online had an NNT of 3 for clinical improvement in behavioral problems in children and U Can POOP Too had an NNT of 4 for prevention of fecal accidents.

**Table 5:** Measures of efficacy for Internet-delivered health-related interventions.

	Target population	Name	Measures of efficacy
**Alcohol**			
	Adult drinkers	Check Your Drinking	After a 3-month period, the Internet group drank 2.4 fewer drinks per week (on average) than the control (information without Internet).
	Male adults	Drinktest.nl	For every 9 people who completed the Internet program, 1 person reduced their drinking levels below recommended levels after 1 month, compared with controls (information without Internet).
	Adult at-risk drinkers	Balance	After use of an Internet program for 6 months, people who had an intensive Internet therapy drank approximately 3 fewer drinks per day than people in the brief self-help program.
	Adult problem drinkers	Drinking Less	For every 9 people who completed the Internet program, 1 person reduced their drinking levels below recommended levels after 6 weeks, compared with controls (informational brochure).
	Universities	Alcohol eCHECKUP TO GO (eCHUG)	Students who were heavy drinkers and completed the Internet intervention reduced their drinking by 8 drinks per week, were less likely to drink to intoxication, and had fewer alcohol-related problems after 3 months than students who did not use this intervention. Binge drinkers who completed the Internet intervention had 5 less drinks (on average) on any given night and were less likely to drink to intoxication after 3 months than students not using the intervention.
	Universities	Alcohol Edu	First year students who participated in the Internet intervention showed a reduction in alcohol use and binge drinking within a 30-day period compared with those who did not use the intervention.
	University students	MyStudentBody	Heavy drinkers who used the Internet intervention had (on average) one-half drink less than those not using the intervention. Women, in particular, lowered their alcohol intake with the Internet intervention.
	University students	Alcohol-Wise	Students at a public urban university who completed the Internet intervention drank (on average) 2 fewer drinks per week than the control (without the intervention).
**Tobacco**			
	Smokers	Smokefree	For every 9 people who used the Internet intervention (without email support), 1 person abstained from smoking for 3 months compared with people who used an abbreviated version of the website. For every 18 people who used the Internet intervention (without email support), 1 person abstained from smoking for 7 months compared with people who used an abbreviated version of the intervention.
	Smokers	QuitCoach	For every 15 people who used the Internet intervention with Web-based structured planning, 1 person abstained from smoking for 6 months compared with people who did not use structured planning.
	Smokers	Stop-tobacco	For every 26 people who completed the Internet intervention, 1 person abstained from smoking for 7 days compared with a modified program (with less information regarding risks and coping).
	Smokers	Slutta (Quit)	For every 24 people who used the tailored Internet intervention, 1 person abstained from smoking for 3 months compared with people who used a nontailored website.
**Cannabis**			
	Cannabis users	Reduce Your Use: How to Break the Cannabis Habit	After 3 months, people who used the Internet intervention had 3 fewer days of cannabis use (per month) compared with people not using the intervention.
	Cannabis users	Quit the Shit	After 3 months, people who used the Internet intervention had 4 fewer days of cannabis use (per 30-day period) compared with people not using the intervention.
**Depression**			
	Individuals with depression	MoodGYM with BluePages	Symptoms of depression decreased by 4 points (on the Centre for Epidemiologic Studies Depression Scale) for people using the Internet intervention, whereas symptoms of depression increased by 3 points for people who were not using the intervention. For every 6 people with depression using the Internet intervention, 1 person could be classified as not having depression after completing the intervention (compared with a control group not using the intervention).
	Individuals with depression	Deprexis	For every 4 people with depression who completed the Internet intervention, 1 person recovered from their depression (compared with people who did not use the Internet intervention).
**Generalized anxiety disorder**			
	Workers	Stress and Mood Management	People using the Internet intervention decreased their stress level to a greater extent (1-point relative decrease on the Symptoms of Distress scale) than people not using the intervention.
	Individuals with anxiety	Internet-based Mindfulness Treatment	After completing the Internet intervention, people decreased their anxiety level (7-point relative decrease on the Beck Anxiety Inventory scale) compared with people who used a discussion forum (control). For every 3 people who completed the Internet intervention, 1 person recovered from their anxiety (compared with people who used a discussion forum).
**Post-traumatic stress disorder**			
	Individuals who experienced disasters	My Disaster Recovery	People who completed the Internet intervention worried less (6-point relative decrease on the Penn State Worry Questionnaire) compared with people who did not use the intervention.
**Panic disorder and phobias**			
	Individuals with panic disorders or phobias	Mental Health Online	After completing the Internet intervention, 67% of the people did not experience any panic attacks in the previous week; this compared with 11% for those who did not use the intervention. The number needed to treat was 2.
	Adults with glossophobia (fear of public speaking)	Talk to Me	People who completed the Internet intervention reduced their fear and avoidance behaviors (3-point relative reduction for fear and 5-point relative reduction for avoidance on 10-point scales) compared with people who did not complete the intervention.
**Diet and physical activity**			
	Overweight and obese adults	The Biggest Loser Club	People who completed the Internet intervention lost 2.1 kg weight, whereas people who did not complete the intervention added 0.4 kg. People who completed the Internet intervention reduced their waist circumference by 2.6 cm, whereas people who did not complete the intervention added 0.3 cm to their waist circumference.
	Managers of organizations	ExecuPrev	Women who completed the Internet intervention reduced their waist circumference by 1.3 inches more than women who did not complete the intervention.
**Insomnia**			
	Adults with chronic insomnia	Insomnie	In people who used the Internet intervention, sleep efficacy increased by 3% above that of people who did not use the intervention (measured at 48 weeks after the start of the intervention). Symptoms of depression also decreased by 3 points (on the Centre for Epidemiologic Studies Depression Scale) for people using the Internet intervention relative to people not using the intervention.
	Adults with insomnia	SHUTi	For people who used the Internet intervention, the severity of insomnia (measured by the Insomnia Severity Index, a 28-point scale) decreased by 8 points relative to the people who did not use the intervention.
	Individuals with sleep problems	Sleepio	In people who used the Internet intervention, sleep efficacy increased by 10% above that of people who did not use the intervention (measured at 8 weeks after the start of the intervention). Daytime performance also improved (by 2.5 points on a 5-point scale) in those who used the Internet intervention compared with those who did use the intervention.
	Adults with chronic insomnia	RESTORE	People who used the Internet intervention improved their sleep quality by 0.5 points (on a 5-point scale) after 4 weeks, whereas the sleep quality of those who did not use the intervention decreased by 0.2 points.
**Chronic pain**			
	Adults with chronic pain	painACTION	For every 4 people with back pain who used the Internet intervention, 1 person experienced improvement compared with a control (text-based material). For every 3 people with migraines who used the Internet intervention, 1 person experienced improvement compared with the control (usual treatment).
**Childhood health problems**			
	Parents with children who have behavior problems	Triple P Online	For every 3 parents who completed the Internet intervention, 1 had a child who experienced clinical improvement in behavioral problems (control group was usual use of the Internet without the intervention). For every 2 parents who completed the Internet intervention, 1 experienced clinical improvement in parental confidence in dealing with behavior problems in their children.
	Parents of children with encopresis	U Can POOP Too	For every 4 parents who completed the Internet intervention, 1 had a child who had no fecal accidents (comparison group was usual care).

## Discussion

### Principal Findings

In this systematic review, we developed a list of Internet health-related programs that demonstrated an evidence-based health benefit. The majority of programs dealt with substance abuse, mental health, or diet and physical activity. In addition, there were Internet programs dealing with disease management such as insomnia and chronic pain, as well as evidence-based Internet therapies for childhood health problems. There were some interventions with considerable efficacy (NNT<5); these included painACTION, Mental Health Online for panic disorders, Deprexis, Triple P Online, and U Can POOP Too.

There were several characteristics of successful Internet-delivered health interventions. First, most of the programs were rather intensive; they required assignments and engagement by the user over the course of weeks to months. For a number of the programs, not only were there interactive elements that prompted personalized feedback and self-monitoring, but also there were assignments that required the user to implement actions when they were not on the Internet such as tracking their sleeping habits via a diary, recording their eating habits throughout the day, or conducting physical activities throughout the week. In all of the therapies, educational materials were presented but these were often adjuncts to the main therapeutic approaches—not the principal tactic. Often the interventions followed cognitive behavioral strategies that were well-grounded in the psychological literature. Thus, most of the successful interventions were not truncated bits of information delivered in a short period of time. They were well-thought out progressive modules of engagement with multilayers of targeted approaches. Many also encouraged individuals to seek professional assistance if further help was needed.

Perhaps the most desirable aspect of having Internet evidence-based programs is the sheer magnitude of the audience. There were 3.5 billion Internet users in the world by December 2016 with a steady increase over the past decade [146]. By providing evidence-based programs, the potential to ameliorate some health problems or behaviors is enormous—even if the completion rates are rather low. The challenge is to determine whether these types of programs work equally well when translated into other languages and delivered to people with different social and cultural backgrounds. More information is needed regarding the triggers of personal readiness to use such programs and what factors appear to serve as enablers to use.

We found that 25 Internet programs were free to the public although some require registration. The availability of free health information removes a key barrier to the public, particularly individuals with lower incomes. The Pew Research Center found that 26% of Internet users who wanted health information were asked to pay, but only 2% of them actually paid for the information [5]. Request for payment resulted in lower-income individuals giving up the search, whereas wealthier individuals sought other avenues for the information [5]. This is of consequence because uninsured and poor individuals tend to have disproportionately higher rates of some health-related behaviors that such programs may help to abate [147].

Therapies that are Internet-based offer an attractive option for certain types of conditions due to easy access and low cost. In some locations, there may be insufficient numbers of clinicians who provide specific therapies, such as cognitive behavioral therapy for insomnia. These programs may also resolve other access problems, such as long wait times or lack of transportation to services. Such programs may be a choice for the first-line of engagement and, if the problem is not resolved, further in-person visits could be arranged. Many of these successful programs provide links to additional resources and some have specific information for health care professionals. The substance abuse websites are particularly strong in providing such links.

Another desirable feature of Internet programs is the ability to reach individuals who shun public places and therefore, are less likely to seek face-to-face care. Our review indicates that there are evidence-based programs for several phobias including social phobias (including shyness), panic attacks, and OCD. Moreover, such programs may reduce the likelihood of social stigma which sometimes occurs when seeking traditional avenues for assistance. For individuals with such problems, Internet programs may have the potential to provide the first step to eventual engagement with medical and neighborhood communities.

Although we anticipated that the Internet would be a valuable location for programs related to sexual health, it was surprising not to find any current evidence-based websites on the prevention of sexually transmitted disease through this review. There were several evidence-based programs for HIV prevention and unintended pregnancy that yielded a health-related benefit, but the programs that were tested did not yield a functional site to continue the program after the completion of the trials.

One of the challenges of Internet-delivered therapies relates to the constraints of the modality itself. There may be problems for individuals with vision problems or those with specific functional disabilities. However, adaptive approaches may be possible to deliver audio programs for those who are blind and modifications may be available for those with specific motor-related disabilities. There are many case studies of computer technologies which have advanced the functional capabilities of those with various limitations; these include approaches which alter input devices, the use of assistive tools for processing, and restructuring the output [148,149].

### Limitations

There are several limitations to this systematic review. There may be some evidence-based websites on health that were missed; we only included those RCTs that were part of a meta-analysis. Therefore, continuous updating will be necessary. This review is but the first step in this process; the development of mechanisms for continuous review is the next. Another limitation was that our focus was on self-help Internet programs. In this review, we did not include Internet-delivered health interventions that integrally involved clinicians, peer-to-peer therapies, or group therapies; an exhaustive review of each of these programs would be helpful for future research studies, so that the breadth of this field could be appreciated and any deficiencies identified. Moreover, this review is meant to initiate the process of dissemination of evidence-based websites and, therefore, additional steps will be necessary. We consider that this process will eventually become analogous to the procedures utilized during dissemination and implementation of conventional medical therapies. That is, RCTs are conducted and reviewed through meta-analysis. This is followed by professional guidelines and recommendations for use, based on the RCT evidence. This, then, typically yields studies which evaluate implementation in the wider population or within specific subgroups. Because these interventions are housed on the Internet, mechanisms for dissemination will involve Internet engagement but will likely require participation of public health professionals, policy makers, and providers of health care.

There are some precautions, however, when delivering Internet therapies directly to the public. Researchers understand that the demonstration of an overall benefit in an RCT relates to a group effect and that this does not necessarily indicate that every single person will receive a benefit. Therefore, part of the implementation process to the public should involve education regarding the limitations of evidence-based Web interventions. They do not guarantee a specific result; they only promise a greater likelihood of a benefit if the therapy is completed.

### Conclusions

We identified several evidence-based health interventions that are currently available on the Internet. They include therapies related to substance abuse, mental health, diet and physical activity, disease management, disease prevention, and childhood health-related problems. Unfortunately, most of the Internet-delivered health interventions that were efficacious through RCTs were not available after the conclusion of the trials. The challenge is to find avenues through governments, organizations, universities, and interested corporations to host the evidence-based Internet programs and to notify the public of their locations. If this process is expanded, such therapies provide hope of a cost-effective mechanism to achieve healthier populations globally.

## Appendix

Multimedia Appendix 1 Home pages of evidence-based online programs.

## Abbreviations

CINAHL: Cumulative Index to Nursing and Allied Health Literature
DASH: Dietary Approaches to Stop Hypertension
NNT: number needed to treat
OCD: obsessive compulsive disorder
PRISMA: Preferred Reporting Items for Systematic Reviews and Meta-Analyses
PTSD: post-traumatic stress disorder
RCT: randomized controlled trial
TPOL: Triple P Online
