# Supplementary material for: Internet-Delivered Health Interventions That Work: Systematic Review of Meta-Analyses and Evaluation of Website Availability
Source: J Med Internet Res. 2017 Mar 24;19(3):e90. doi: 10.2196/jmir.7111 (PMC5384996; doi:10.2196/jmir.7111)
Supplement: Multimedia Appendix 1 [file jmir_v19i3e90_app1.pdf]

Home Pages of Evidence-Based Online Programs that Improved Health

Check Your Drinking

Archived at: http://www.webcitation.org/6mYgJmCfj

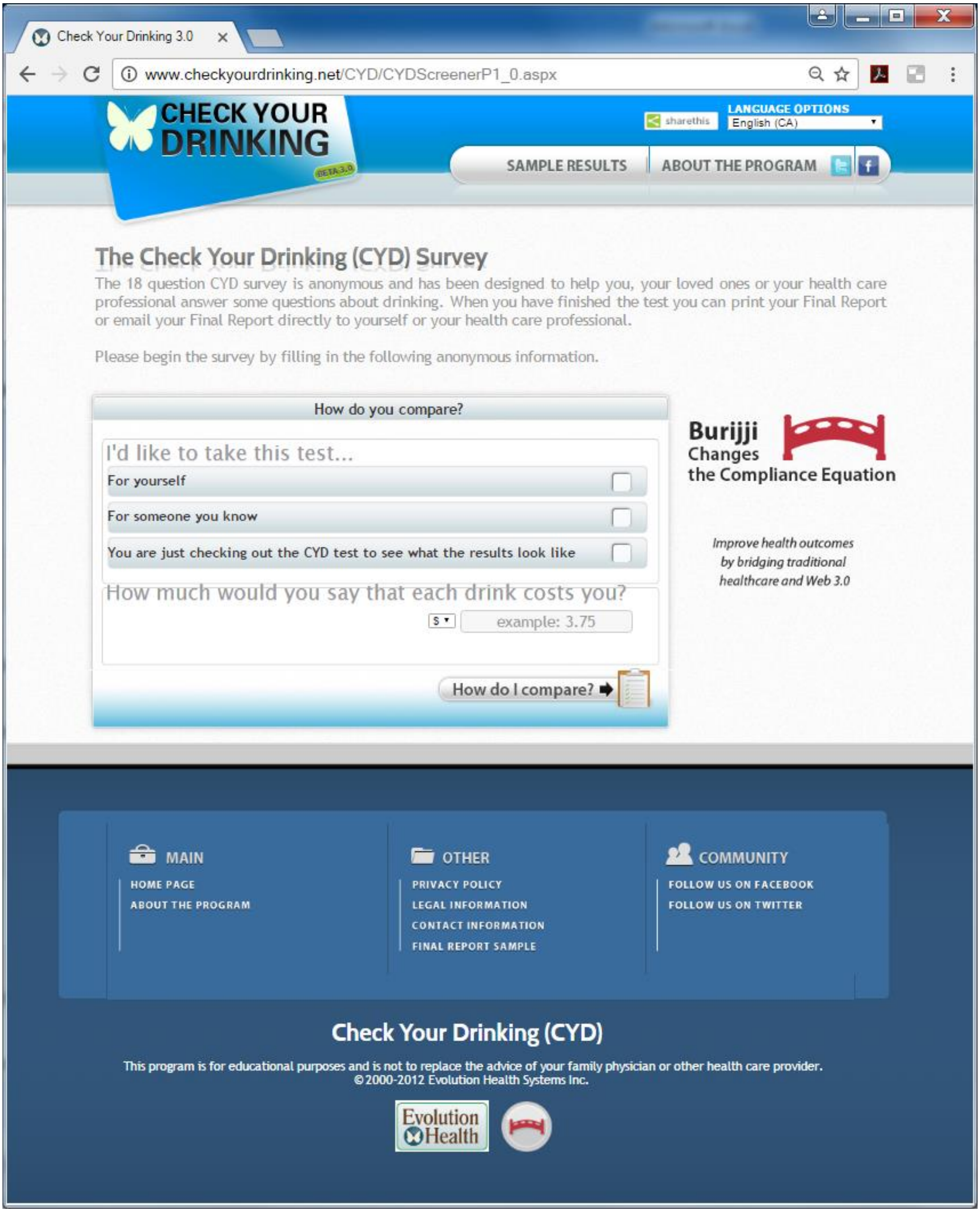

Drinktest

Archived at: http://www.webcitation.org/6mYgXiK7Q

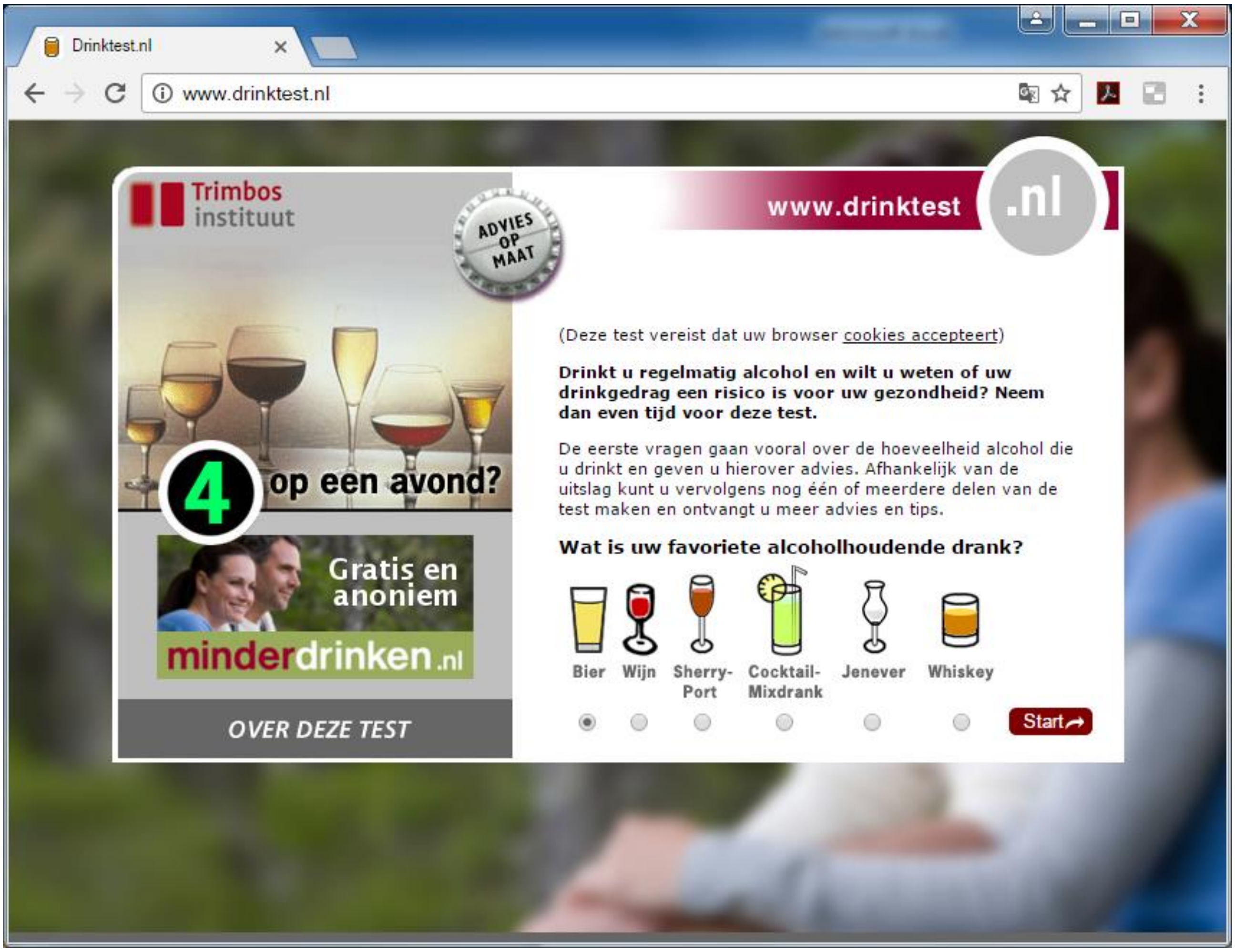

Balance

Archived at: http://www.webcitation.org/6mYghMiJq

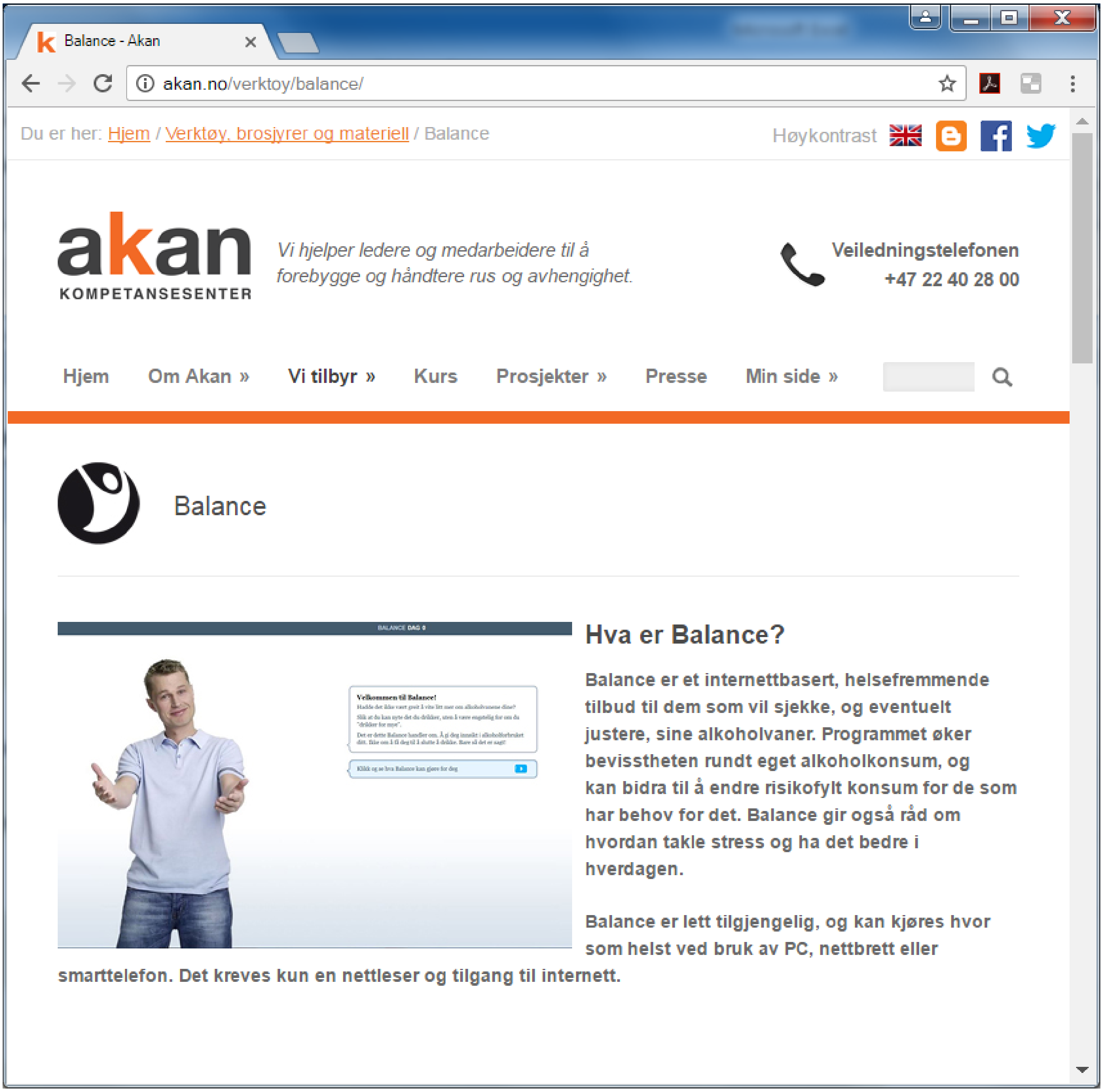

Balance - Akan

akan.no/verktoy/balance/

Du er her: Hjem / Verktøy, brosjyrer og materiell / Balance

Høykontrast

akan KOMPETANSESENTER

Vi hjelper ledere og medarbeidere til å forebygge og håndtere rus og avhengighet.

Veiledningstelefonen
+47 22 40 28 00

Hjem | Om Akan » | Vi tilbyr » | Kurs | Prosjekter » | Presse | Min side »

Balance

Hva er Balance?

Balance er et internettbasert, helsefremmende tilbud til dem som vil sjekke, og eventuelt justere, sine alkoholvaner. Programmet øker bevisstheten rundt eget alkoholkonsum, og kan bidra til å endre risikofylt konsum for de som har behov for det. Balance gir også råd om hvordan takle stress og ha det bedre i hverdagen.

Balance er lett tilgjengelig, og kan kjøres hvor som helst ved bruk av PC, nettbrett eller smarttelefon. Det kreves kun en nettleser og tilgang til internett.

Drinking Less

Archived at: http://www.webcitation.org/6mYgv5ULr

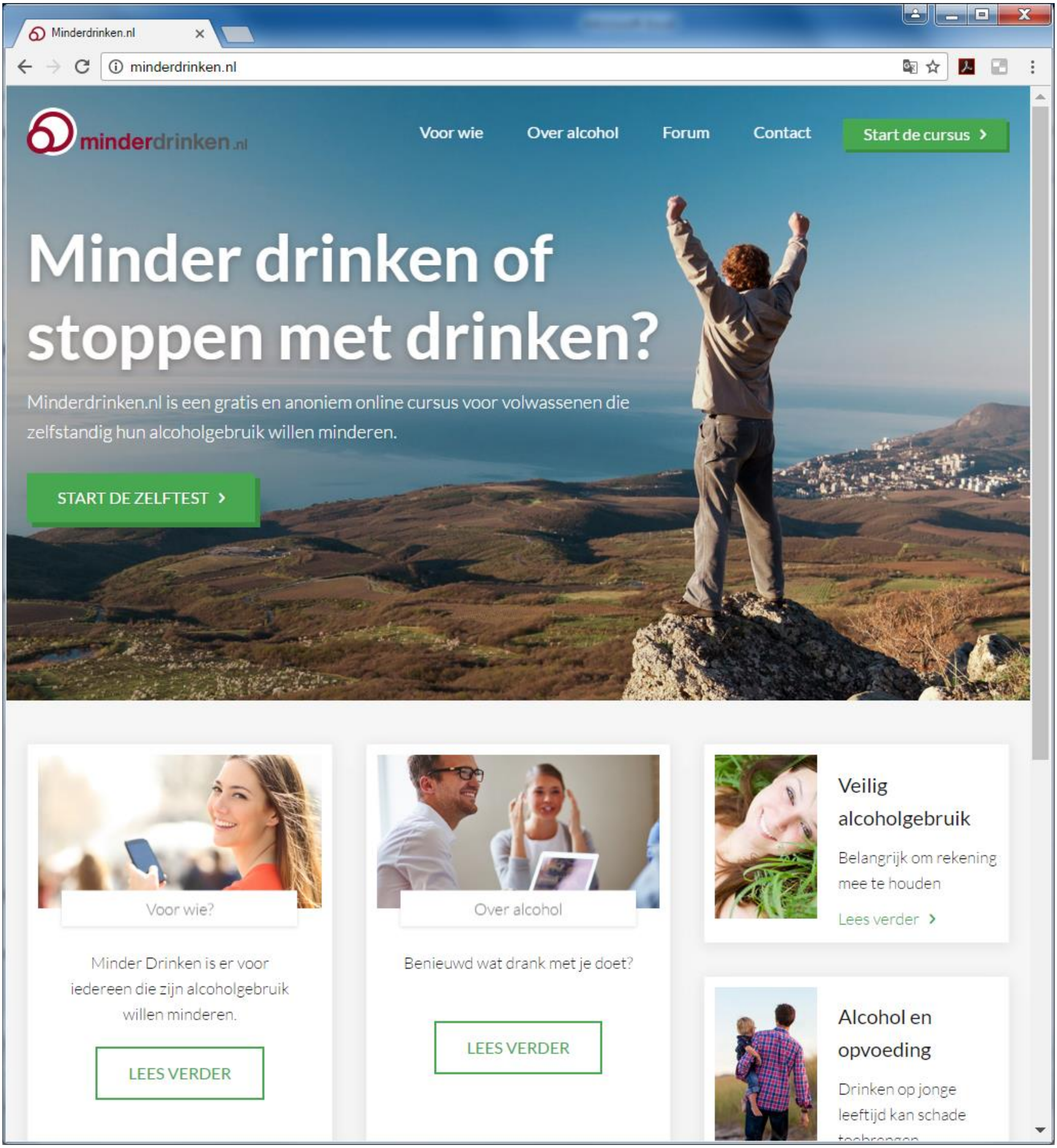

Alcohol eCHECKUP TO GO (eCHUG)

Archived at: http://www.webcitation.org/6mYh1USjU

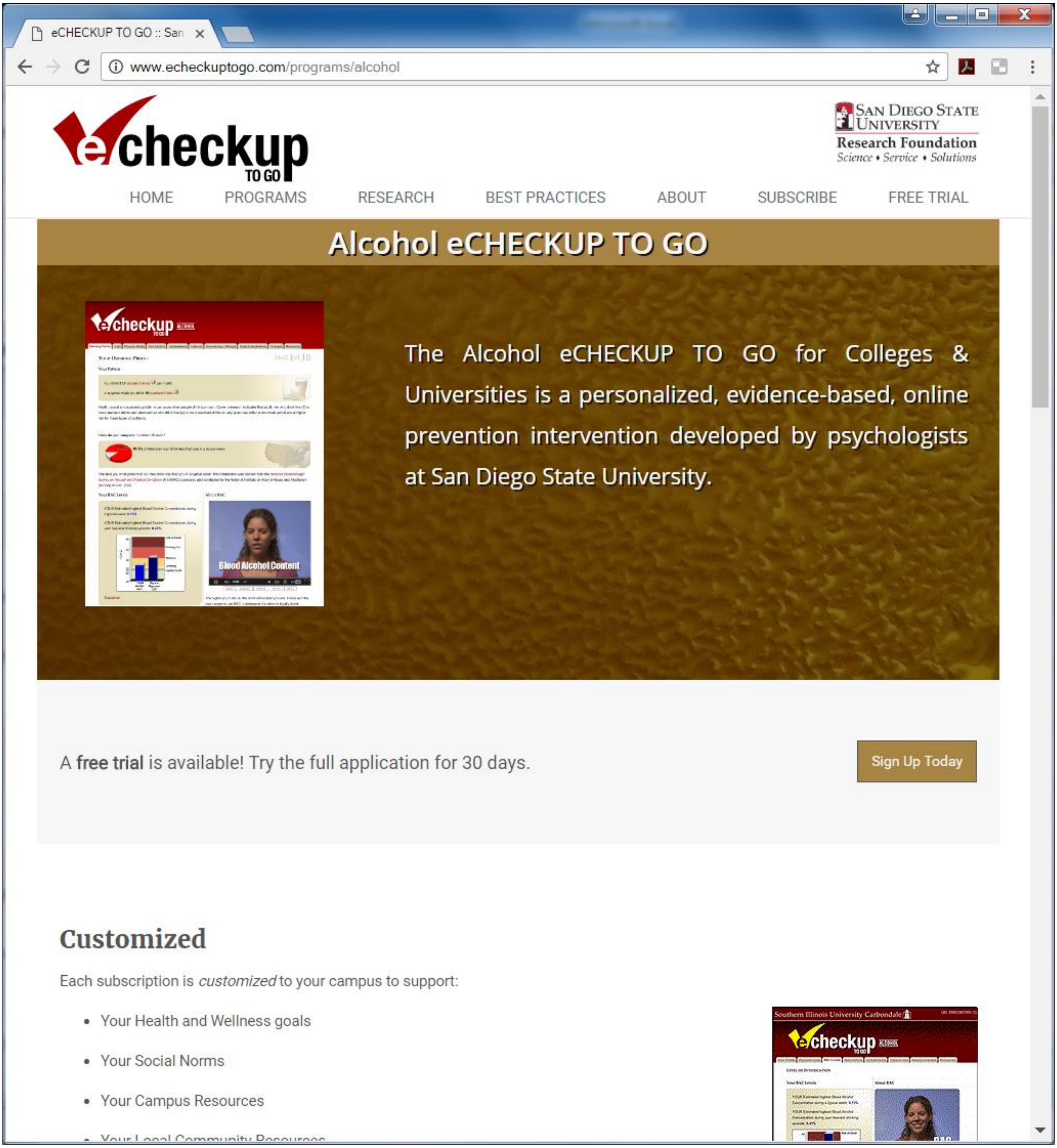

Alcohol Edu

Archived at: http://www.webcitation.org/6mYhCe4M9

Health & Wellness - EverFi

https://everfi.com/health-wellness/

REQUEST A DEMO | Login | Support

EVERFI

OUR FOCUS | MARKETS | SKILL AREAS | SPONSORS | BLOG | ABOUT

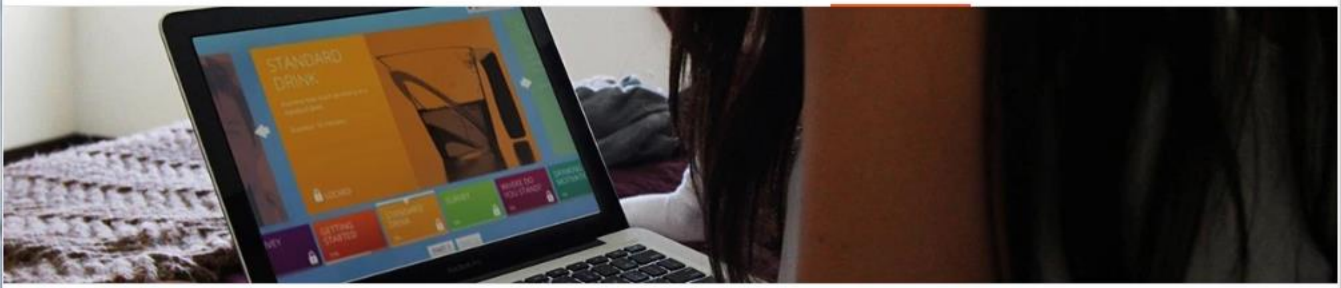

Health & Wellness

Select a Skill Area

Inspiring students and employees to lead safer, healthier, and more productive lives

Our mission is to drive lasting, large-scale change on critical wellness issues facing students and employees. We help organizations make transformative impact on sexual assault, high-risk drinking, and broader wellness through evidence-based online programs, data, and advisory services.

Solutions by Market

K-12

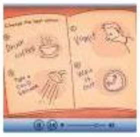

AlcoholEdu® for High School

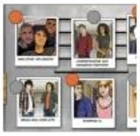

Healthy Relationships

Higher Education

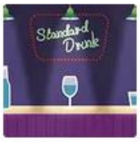

AlcoholEdu® for College

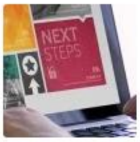

Haven - Understanding Sexual Assault™

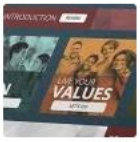

GreekLifeEdu®

My Student Body

Archived at: http://www.webcitation.org/6mYhHw5LF

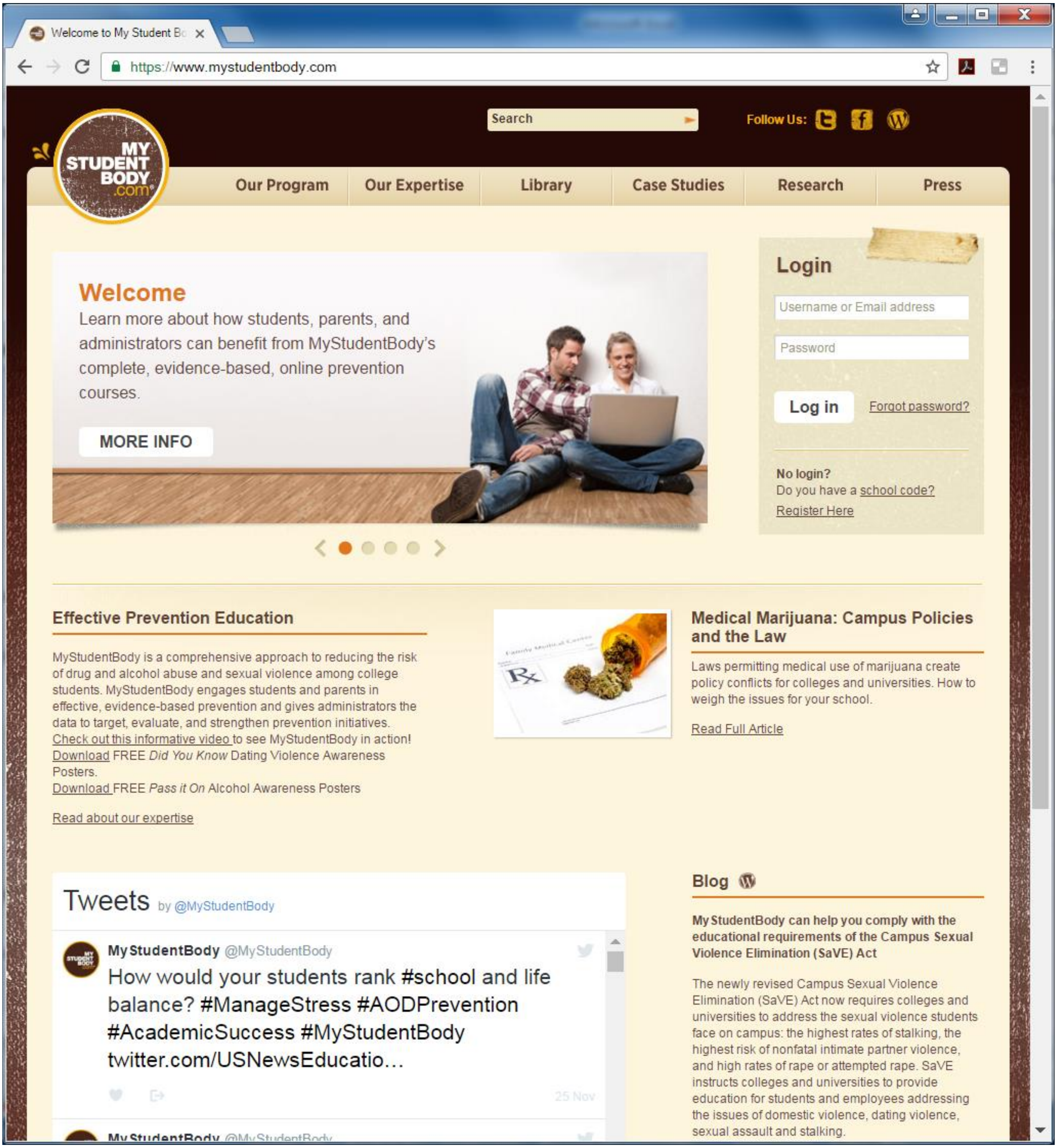

Welcome to My Student Bo

https://www.mystudentbody.com

Search

Follow Us:

MY STUDENT BODY .com

Our Program | Our Expertise | Library | Case Studies | Research | Press

Welcome

Learn more about how students, parents, and administrators can benefit from MyStudentBody's complete, evidence-based, online prevention courses.

MORE INFO

Login

Username or Email address

Password

Log in

Forgot password?

No login?

Do you have a school code?

Register Here

Effective Prevention Education

MyStudentBody is a comprehensive approach to reducing the risk of drug and alcohol abuse and sexual violence among college students. MyStudentBody engages students and parents in effective, evidence-based prevention and gives administrators the data to target, evaluate, and strengthen prevention initiatives.

Check out this informative video to see MyStudentBody in action!

Download FREE *Did You Know* Dating Violence Awareness Posters.

Download FREE *Pass it On* Alcohol Awareness Posters

Read about our expertise

Medical Marijuana: Campus Policies and the Law

Laws permitting medical use of marijuana create policy conflicts for colleges and universities. How to weigh the issues for your school.

Read Full Article

Tweets by @MyStudentBody

MyStudentBody @MyStudentBody

How would your students rank #school and life balance? #ManageStress #AODPrevention #AcademicSuccess #MyStudentBody twitter.com/USNewsEducatio…

25 Nov

MyStudentBody @MyStudentBody

Blog

MyStudentBody can help you comply with the educational requirements of the Campus Sexual Violence Elimination (SaVE) Act

The newly revised Campus Sexual Violence Elimination (SaVE) Act now requires colleges and universities to address the sexual violence students face on campus: the highest rates of stalking, the highest risk of nonfatal intimate partner violence, and high rates of rape or attempted rape. SaVE instructs colleges and universities to provide education for students and employees addressing the issues of domestic violence, dating violence, sexual assault and stalking.

Alcohol-Wise

Archived at: http://www.webcitation.org/6mYhU0TXm

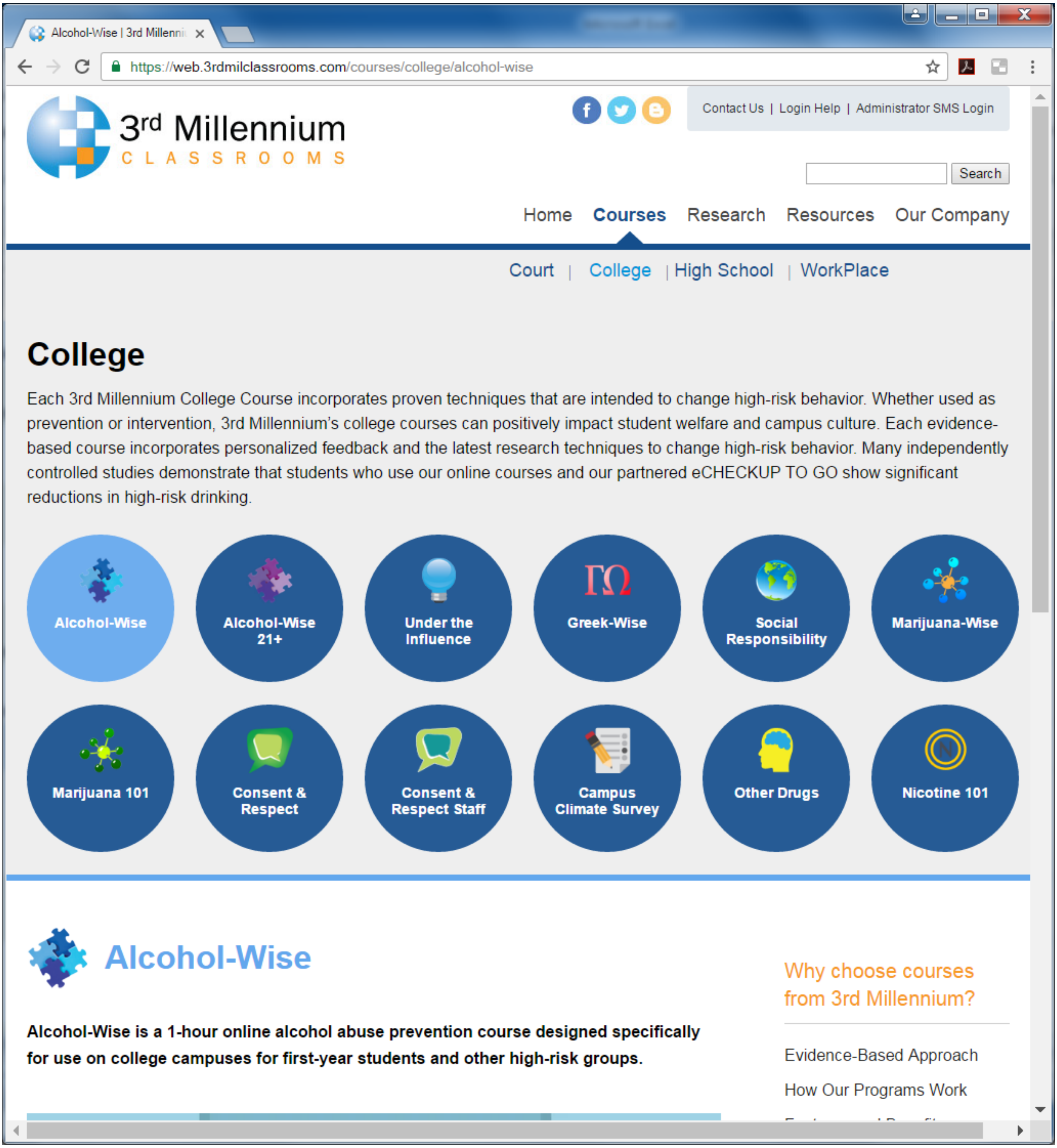

Alcohol-Wise | 3rd Millenni

https://web.3rdmilclassrooms.com/courses/college/alcohol-wise

3rd Millennium CLASSROOMS

Contact Us | Login Help | Administrator SMS Login

Search

Home Courses Research Resources Our Company

Court | College | High School | WorkPlace

College

Each 3rd Millennium College Course incorporates proven techniques that are intended to change high-risk behavior. Whether used as prevention or intervention, 3rd Millennium's college courses can positively impact student welfare and campus culture. Each evidence-based course incorporates personalized feedback and the latest research techniques to change high-risk behavior. Many independently controlled studies demonstrate that students who use our online courses and our partnered eCHECKUP TO GO show significant reductions in high-risk drinking.

Alcohol-Wise
Alcohol-Wise 21+
Under the Influence
Greek-Wise
Social Responsibility
Marijuana-Wise
Marijuana 101
Consent & Respect
Consent & Respect Staff
Campus Climate Survey
Other Drugs
Nicotine 101

Alcohol-Wise

Alcohol-Wise is a 1-hour online alcohol abuse prevention course designed specifically for use on college campuses for first-year students and other high-risk groups.

Why choose courses from 3rd Millennium?

Evidence-Based Approach

How Our Programs Work

Smokefree

Archived at: http://www.webcitation.org/6mYhc49dh

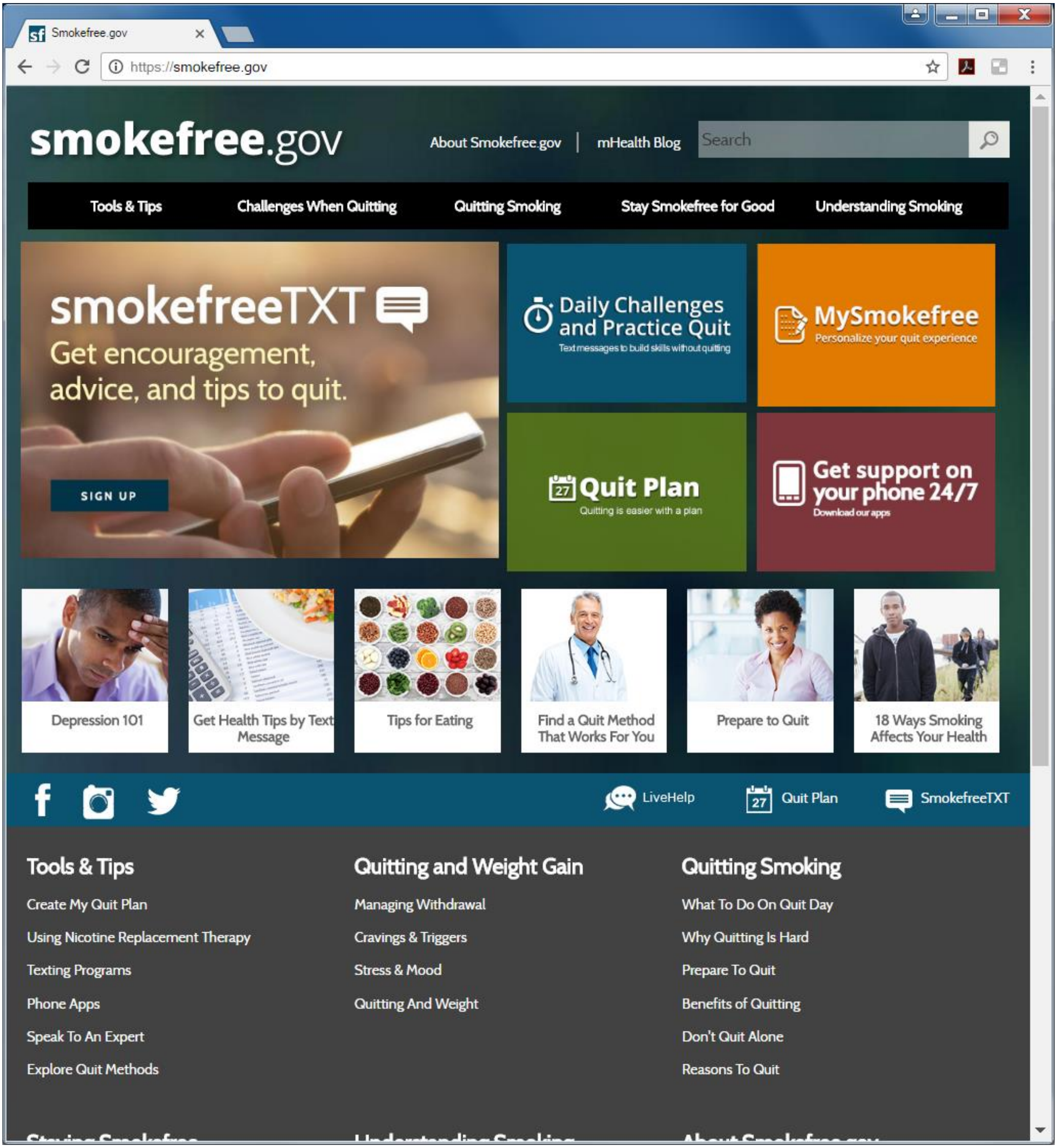

QuitCoach

Archived at: http://www.webcitation.org/6mYhwFhU4

QuitCoach > Home

www.quitcoach.org.au

QuitCoach

Quitline 137848

Join QuitCoach

Get expert advice to help you quit smoking and stay quit forever!

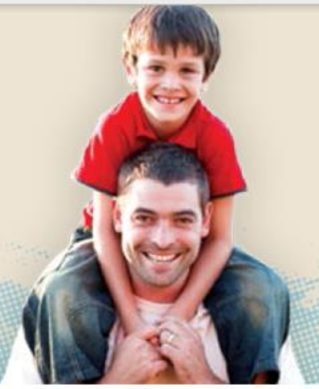

What is QuitCoach?

QuitCoach provides you with a personalised quitting plan. It creates this plan based on your responses to questions about your motivation, confidence and past attempts. It is designed to provide useful advice wherever you are on your quitting journey.

QuitCoach is free, and has helped thousands to achieve their goal of becoming a nonsmoker. Research shows it increases your chances of quitting.[1]

Quitting smoking is a process that can take time. QuitCoach works best if you return and review your situation when things have changed e.g. after you quit. QuitCoach then asks questions relevant to your current situation and will adjust your quitting plan to reflect the progress you have made. It can also help you to recover if things don't go to plan.

If you would also like to receive regular reminders to keep on track, join Quit's new SMS service, QuitTxt. QuitTxt works well in partnership with QuitCoach.

How QuitCoach Helps You

QuitCoach gives you advice to help you:

- understand your addiction
- know what to expect when you quit
- have strategies to help you resist urges to smoke
- know what tools are available to help you

Quitting is a process and QuitCoach can help you with all of the key challenges, such as:

- making the decision to quit
- setting a quit date and actually stopping
- dealing with urges to smoke and nicotine withdrawal
- dealing with situations where you used to smoke
- starting to think and feel like a nonsmoker

Already a member of QuitCoach or QuitTxt?

Username:

Password:

Log in

Not a member of QuitCoach or QuitTxt?

Join now

Enter as guest

Password Retrieval

Please enter your email address to retrieve your password

Stop-tobacco

Archived at: http://www.webcitation.org/6mYluqZeC

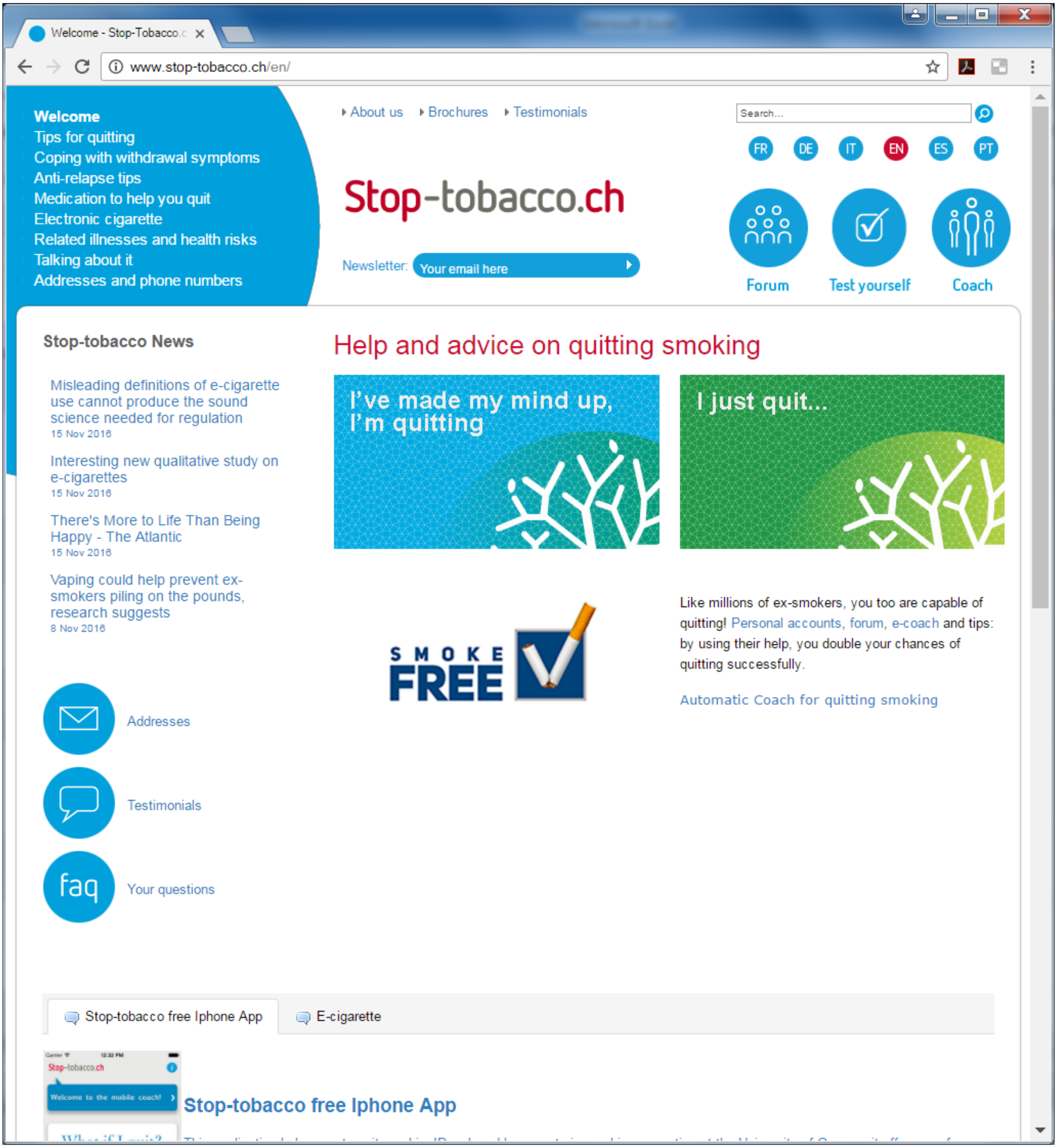

Give Up Smoking

Archived at: http://www.webcitation.org/6mYi7EMYi

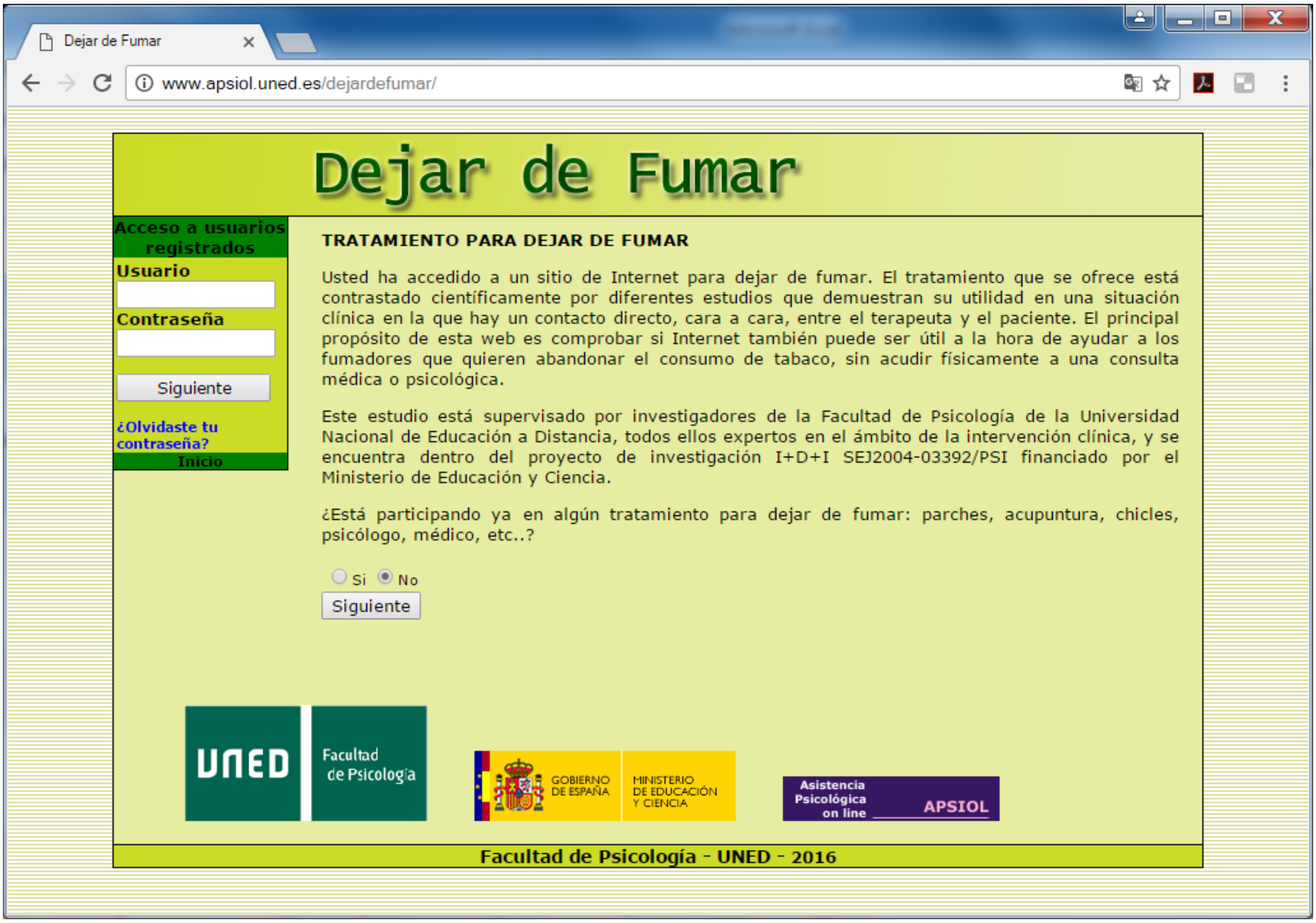

Guide to Quitting Smoking

Archived at: http://www.webcitation.org/6mYiD7Mh9

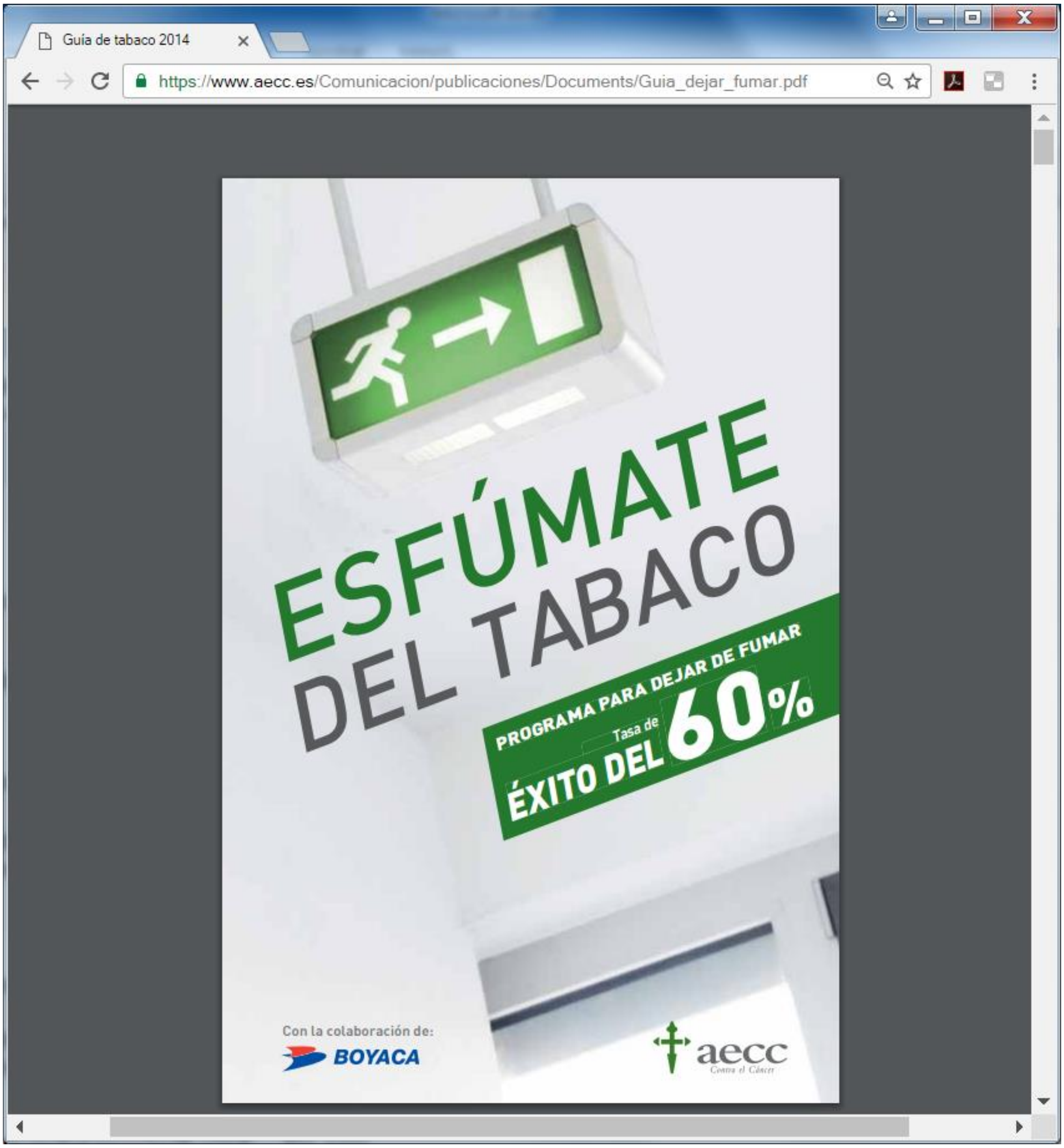

Quit

Archived at: http://www.webcitation.org/6mYiHsRm2

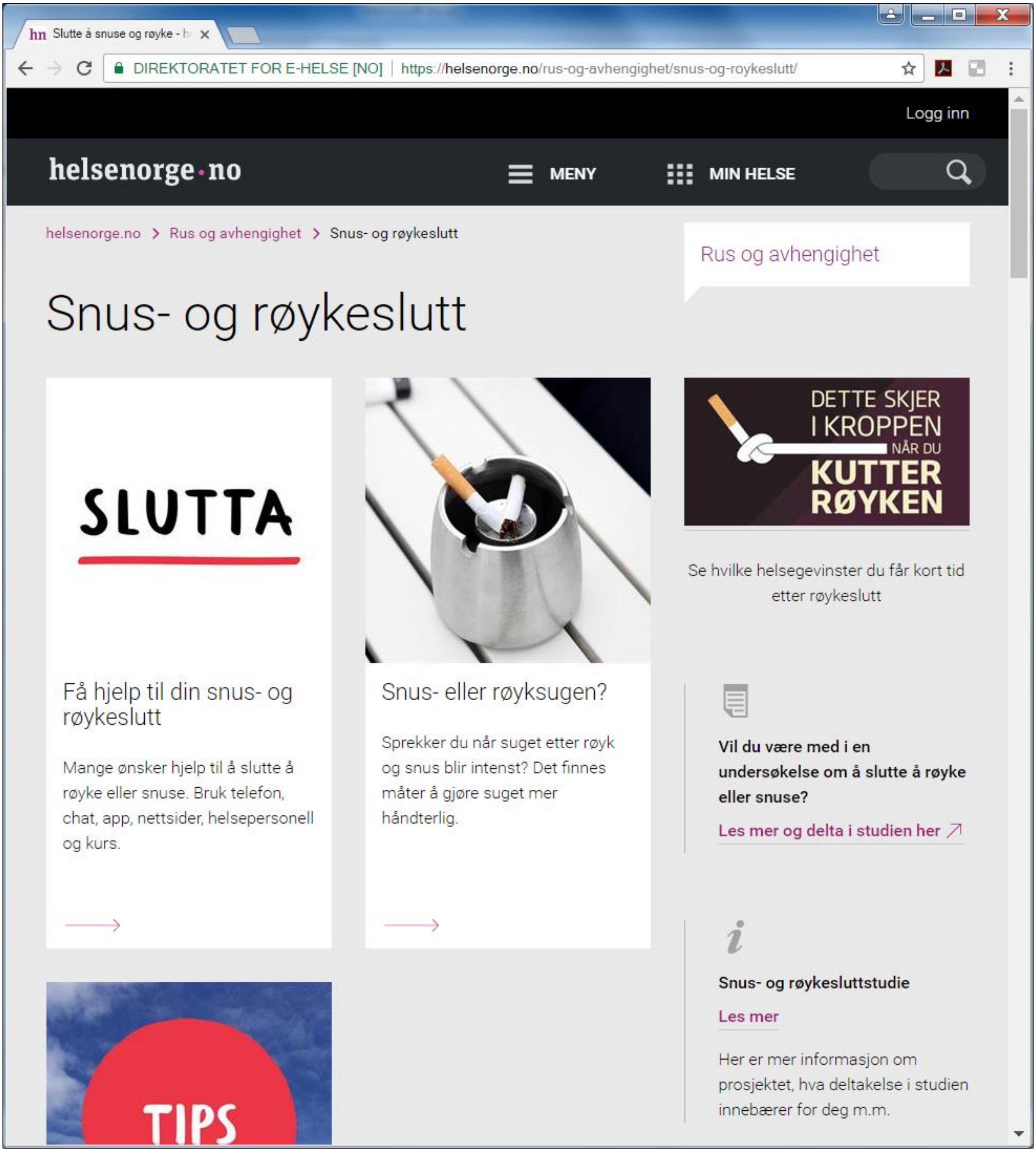

QuitNet

Archived at: http://www.webcitation.org/6mYiMLeFO

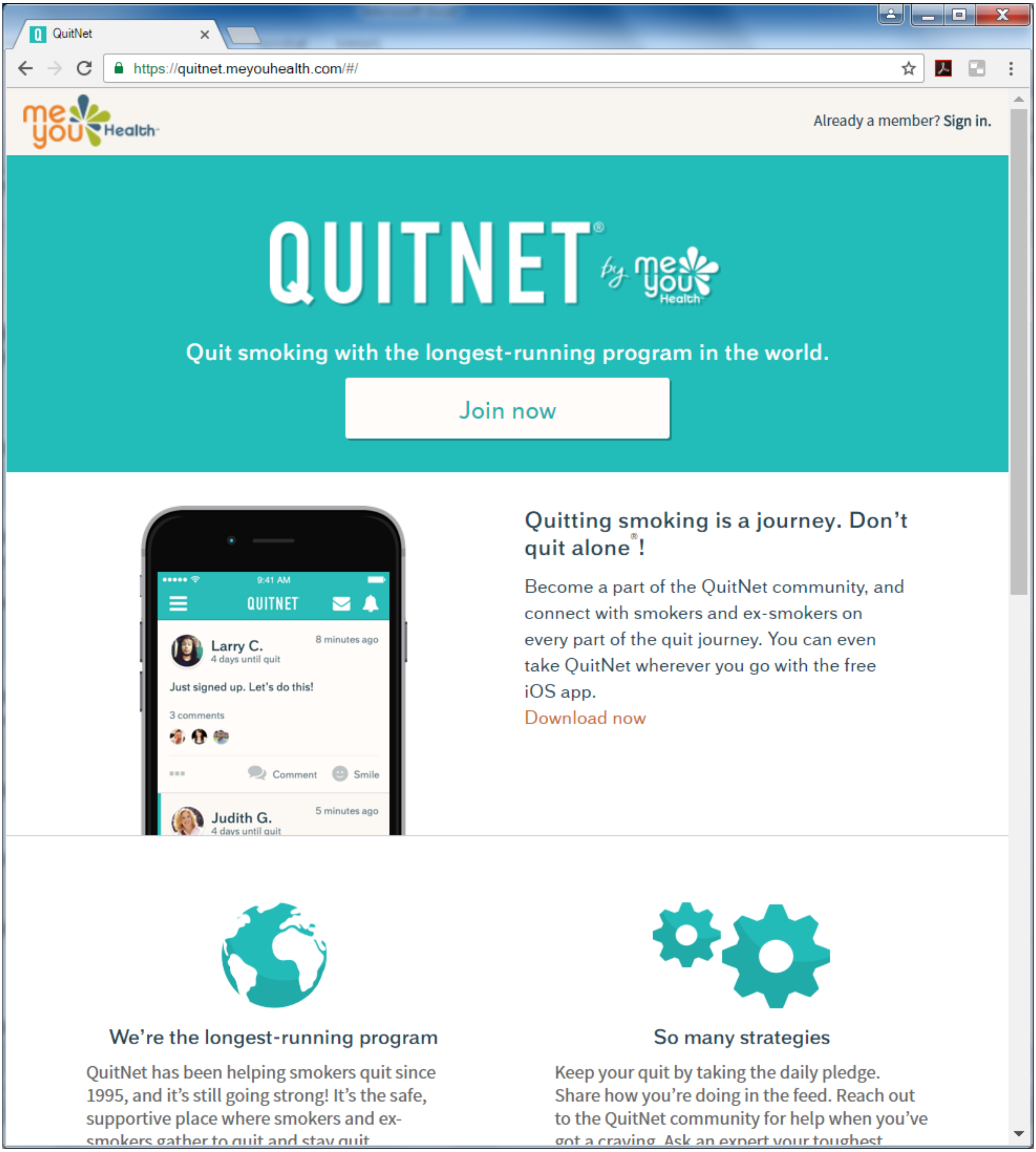

Reduce Your Use

Archived at: http://www.webcitation.org/6mYiU6kjC

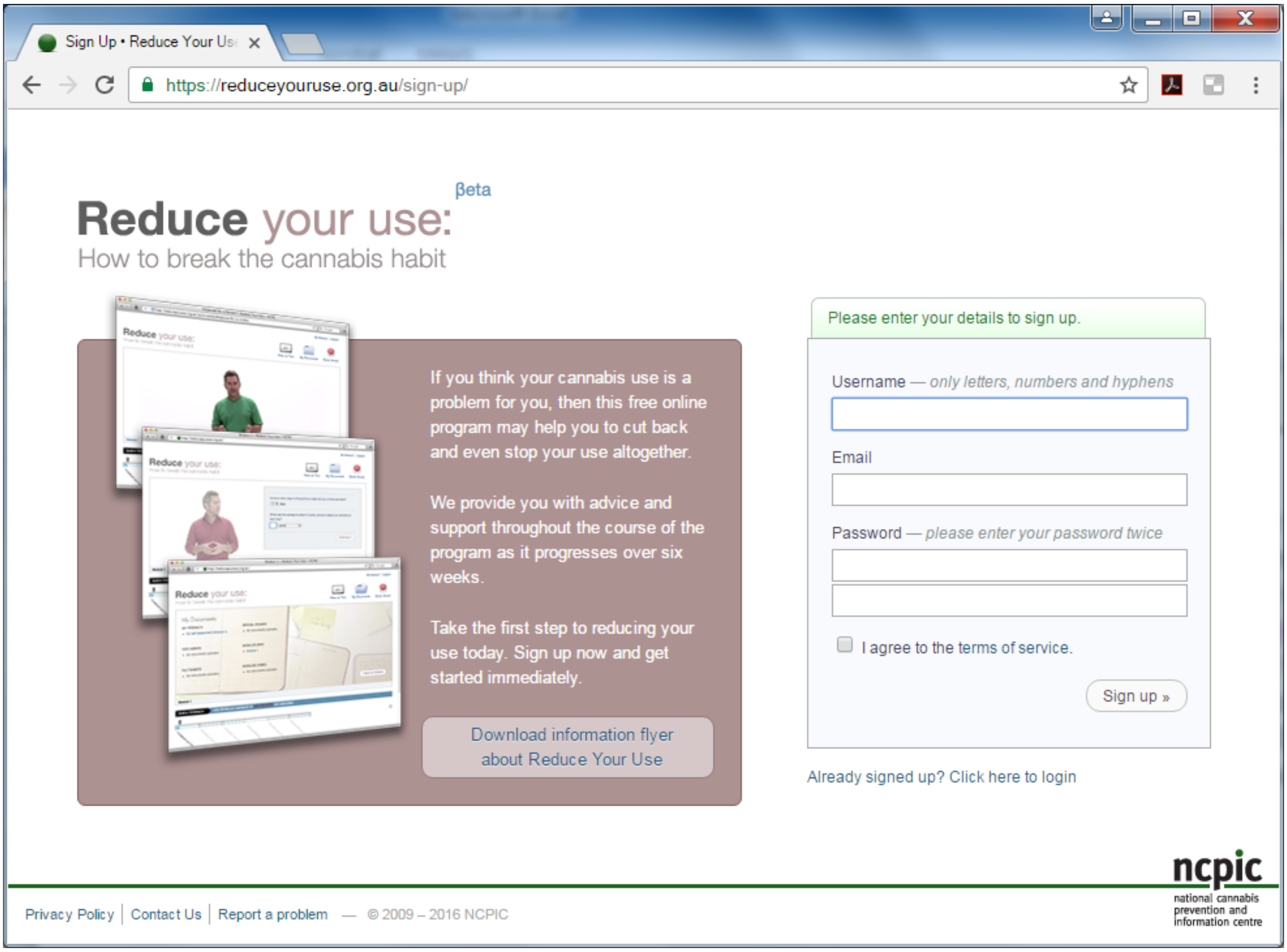

Quit the Shit

Archived at: http://www.webcitation.org/6mYiYUee2

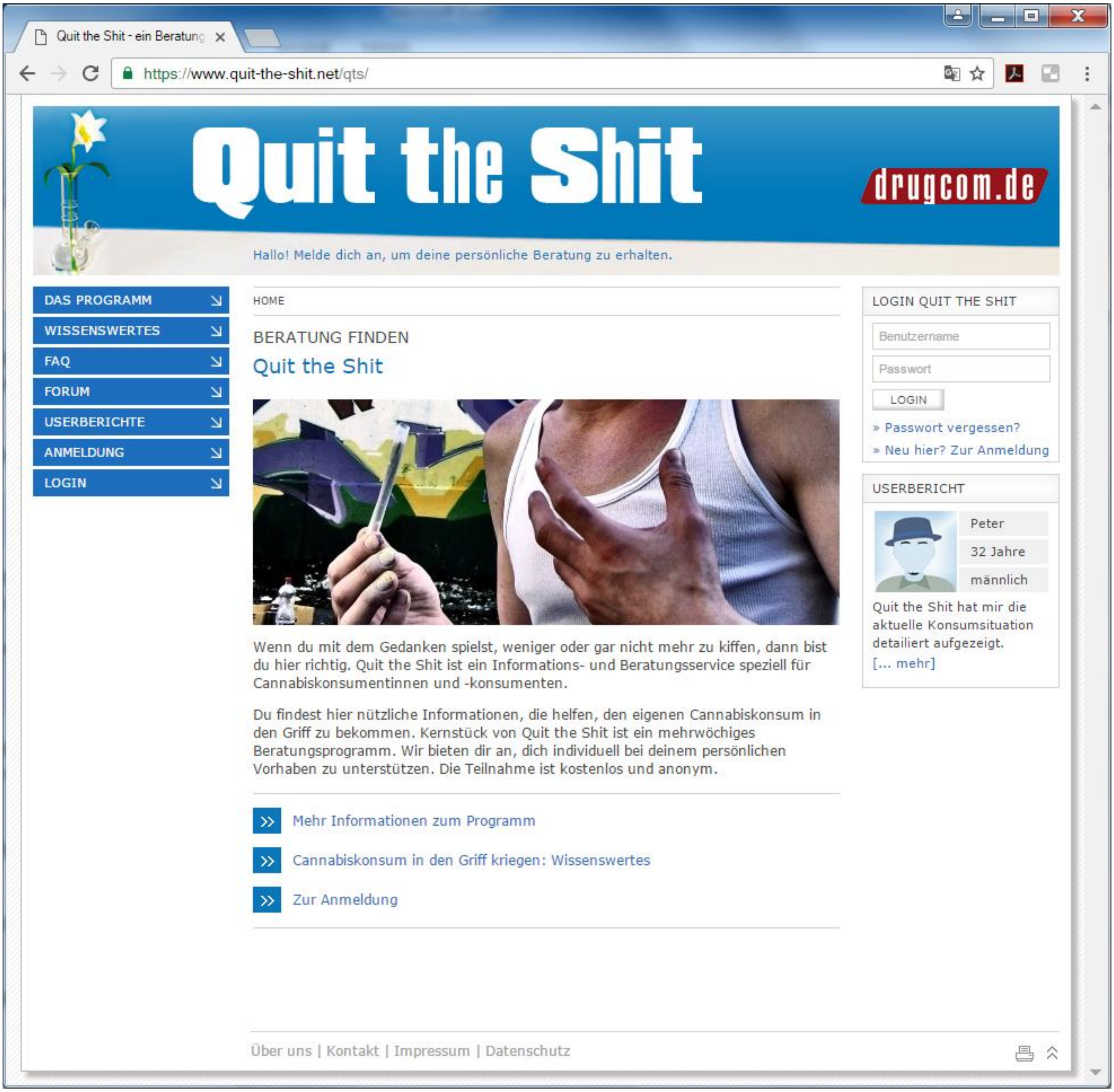

Quit the Shit - ein Beratung

https://www.quit-the-shit.net/qts/

Quit the Shit

drugcom.de

Hallo! Melde dich an, um deine persönliche Beratung zu erhalten.

- DAS PROGRAMM
- WISSENSWERTES
- FAQ
- FORUM
- USERBERICHTE
- ANMELDUNG
- LOGIN

HOME

BERATUNG FINDEN

Quit the Shit

Wenn du mit dem Gedanken spielst, weniger oder gar nicht mehr zu kiffen, dann bist du hier richtig. Quit the Shit ist ein Informations- und Beratungsservice speziell für Cannabiskonsumentinnen und -konsumenten.

Du findest hier nützliche Informationen, die helfen, den eigenen Cannabiskonsum in den Griff zu bekommen. Kernstück von Quit the Shit ist ein mehrwöchiges Beratungsprogramm. Wir bieten dir an, dich individuell bei deinem persönlichen Vorhaben zu unterstützen. Die Teilnahme ist kostenlos und anonym.

» Mehr Informationen zum Programm

» Cannabiskonsum in den Griff kriegen: Wissenswertes

» Zur Anmeldung

LOGIN QUIT THE SHIT

Benutzername

Passwort

LOGIN

» Passwort vergessen?

» Neu hier? Zur Anmeldung

USERBERICHT

Peter

32 Jahre

männlich

Quit the Shit hat mir die aktuelle Konsumsituation detailiert aufgezeigt.

[... mehr]

Über uns | Kontakt | Impressum | Datenschutz

MoodGYM

Archived at: http://www.webcitation.org/6mYie9JlI

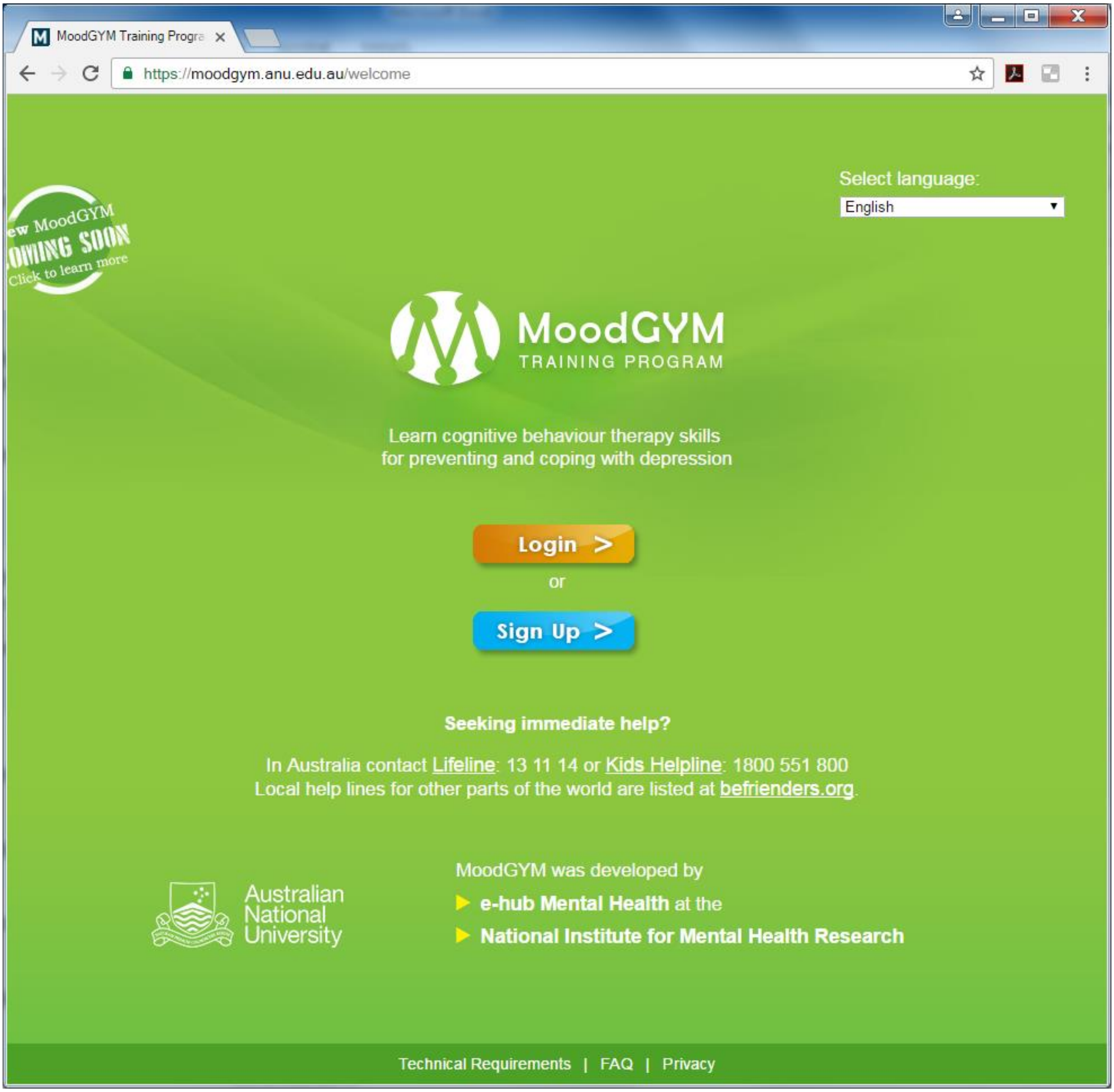

BluePages

Archived at: http://www.webcitation.org/6mYiigPaJ

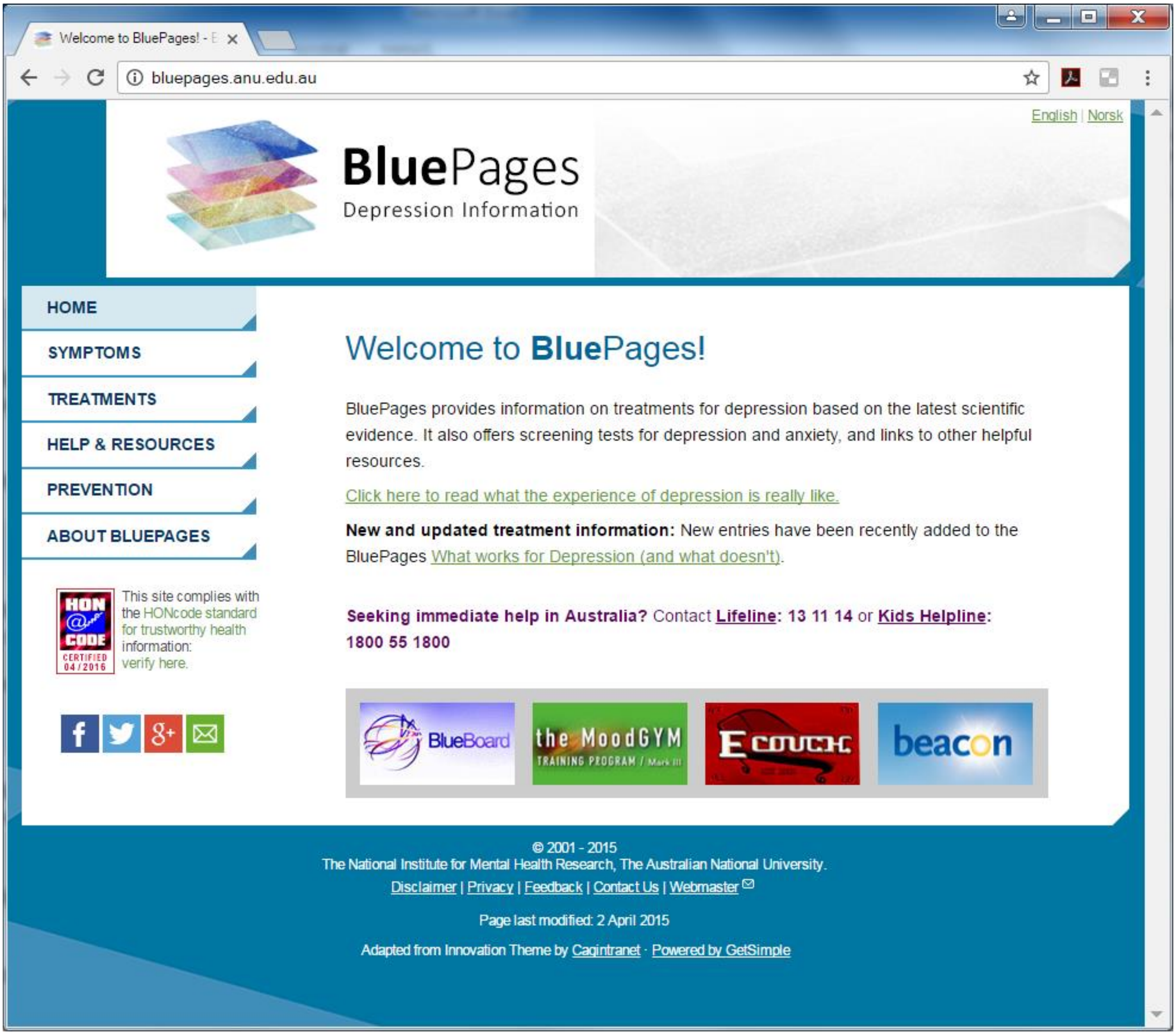

Deprexis

Archived at: http://www.webcitation.org/6mYinoNcW

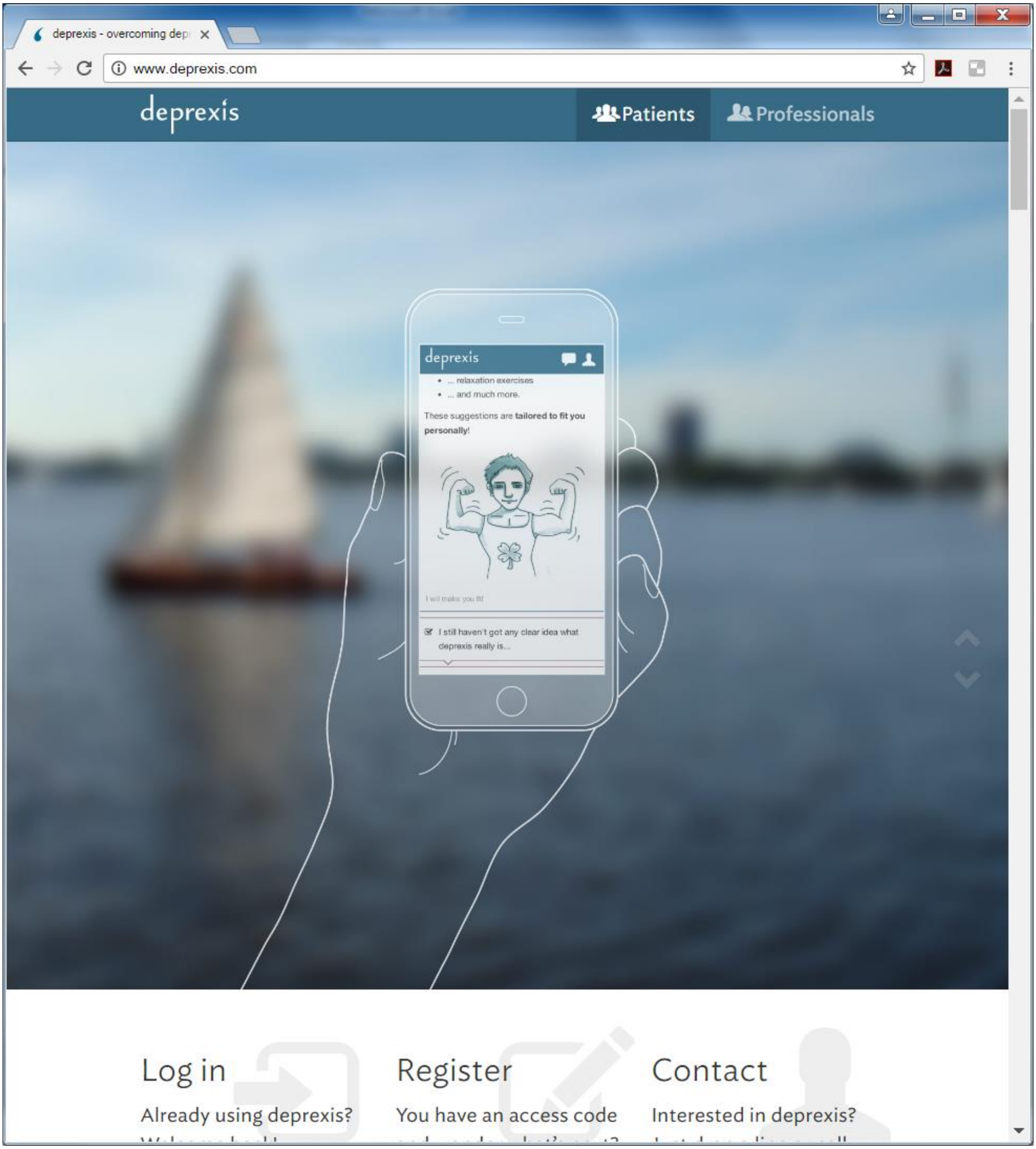

Color Your Life

Archived at: http://www.webcitation.org/6mYisjzyh

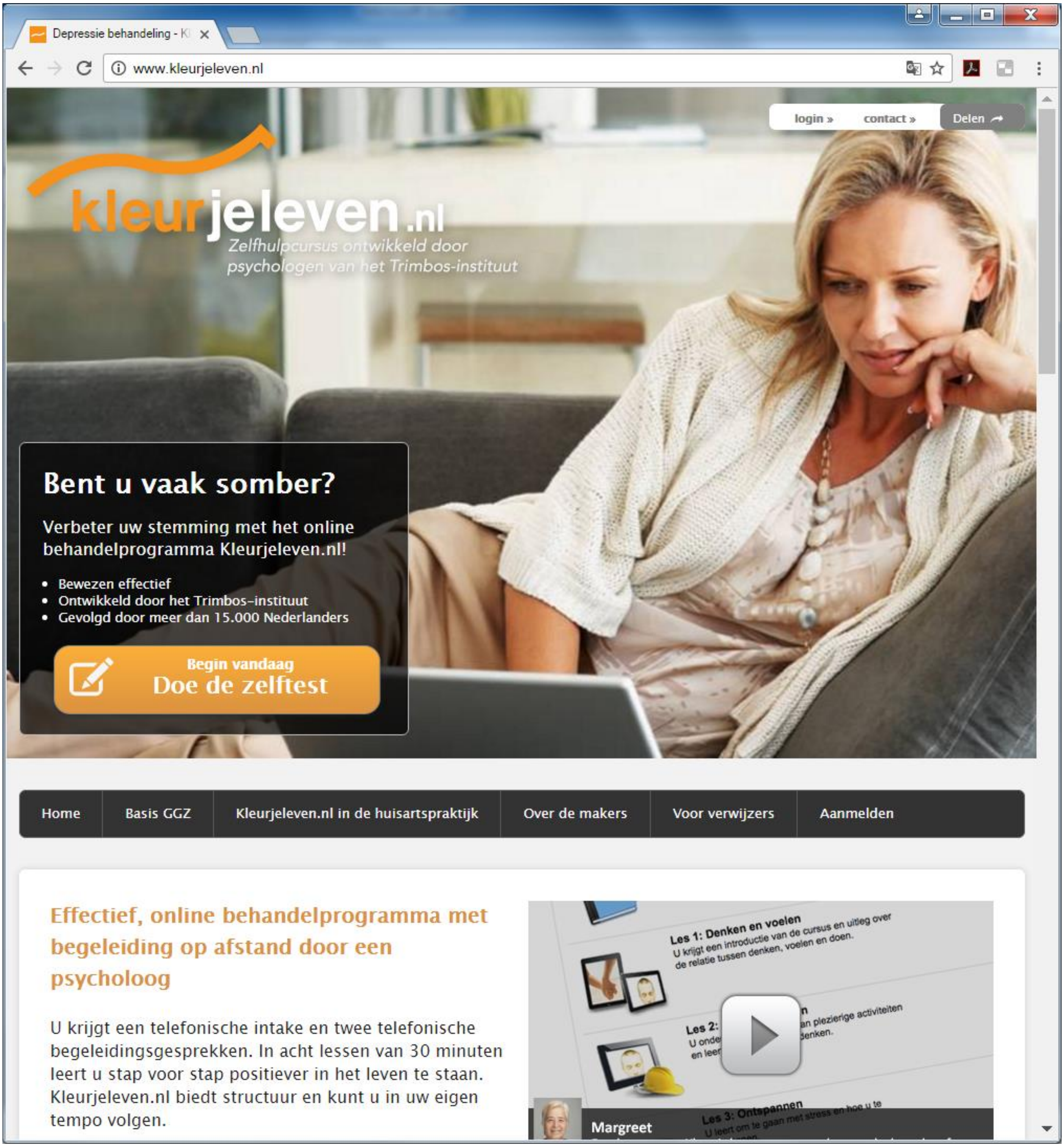

Mental Health Online

Archived at: http://www.webcitation.org/6mYj15VeL

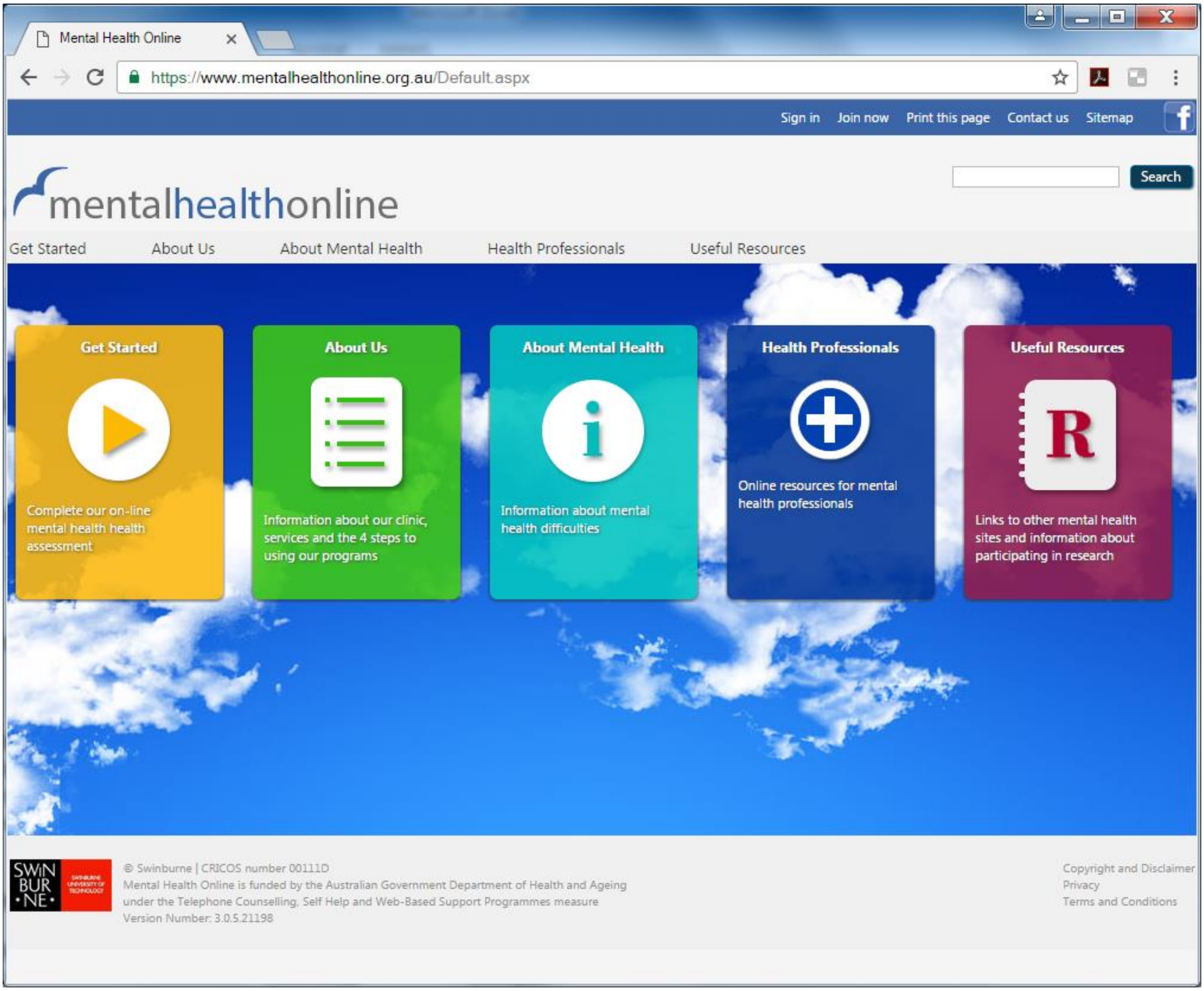

This Way Up

Archived at: http://www.webcitation.org/6mYjG4I5J

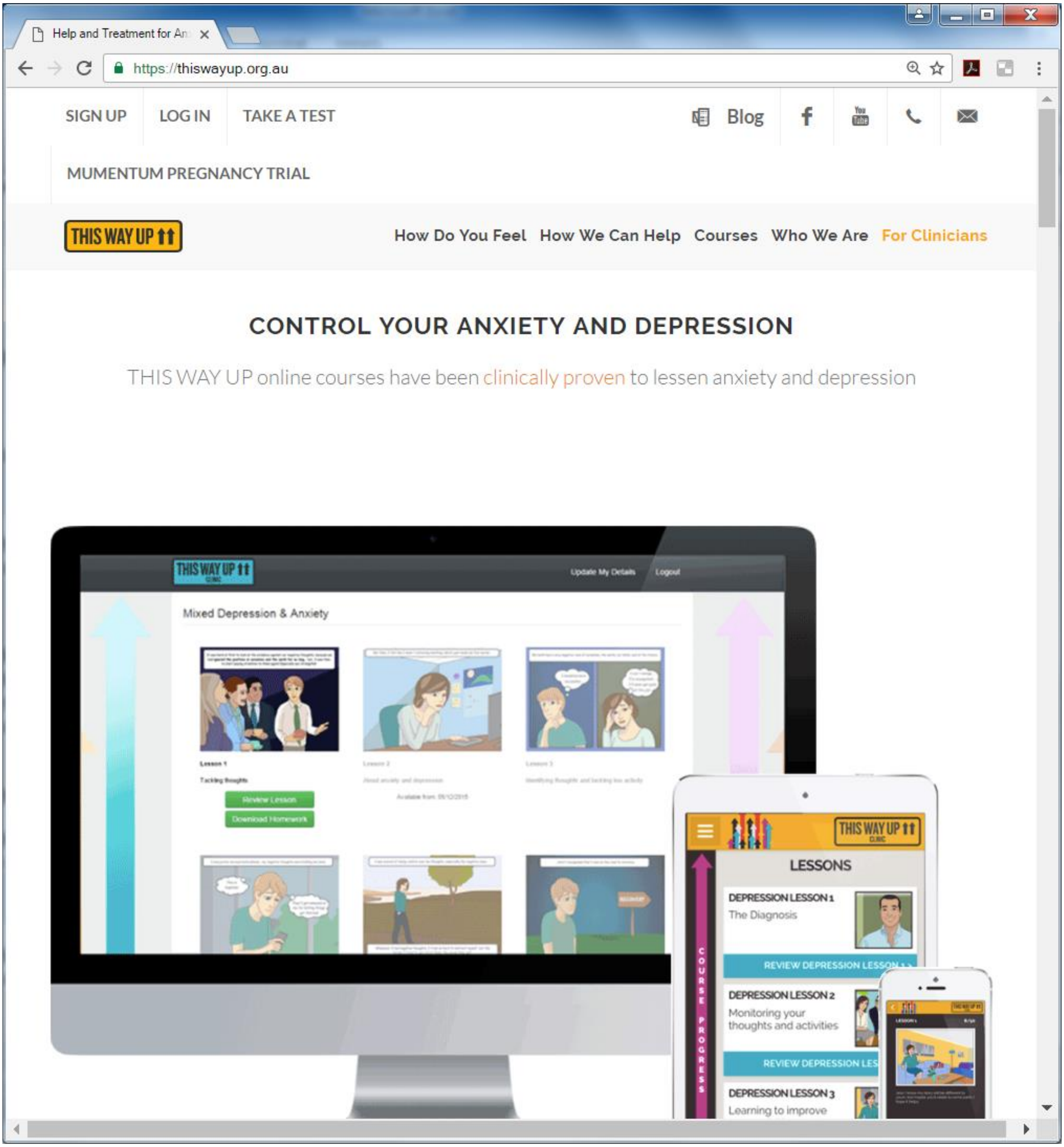

Stress and Mood Management

Archived at: http://www.webcitation.org/6mYjKc4DL

Stress and Mood Managem ×

centerforworkforcehealth.com/programs/stress-and-mood-management/

Center for Workforce Health
Improving the Health and Productivity of Today's Workforce

home | programs | approach | who we are | outcome studies | clients & partners | contact

Our Programs

Back to Program List

Stress and Mood Management

Overview

Stress & Mood Management is designed to help build resiliency and skills to tackle the strains and stresses of everyday life. Developed through a grant from the National Institute of Mental Health, this program includes segments on the disorders of depression and anxiety, as well as a variety of effective stress management techniques.

Approach

Stress & Mood Management contains a vast amount of information about stress, depression, and anxiety. Although aimed at people in the workplace, the content is of value to anyone interested in these mental health issues. The site provides self-assessment tools to gauge levels of stress, anxiety or depression, and includes information on the body's response to stress and the physiology associated with depression and anxiety. Through the use of full narration, animation, text, and graphics, the program gives helpful strategies and tools to help minimize the negative effects of these states. The program also addresses the dangers of self-medication though alcohol and drugs and provides information on treatments that work. Finally, the program features a useful resource section with links to government and private organizations that handle mental health issues.

Evidence

The NIH-supported randomized trial conducted at America Online demonstrated significant positive effects on stress, alcohol consumption and knowledge of depression and anxiety disorders. See Outcome Studies for information on the article published in the *Journal of Occupational and Environmental Medicine.*

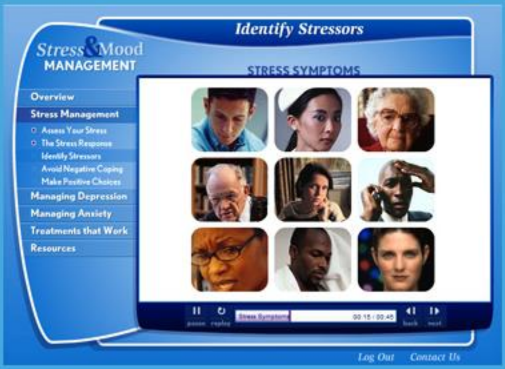

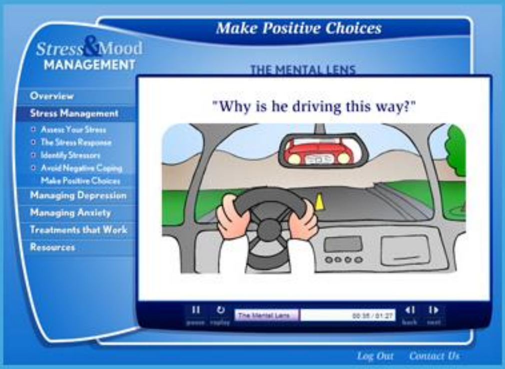

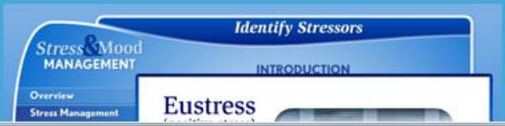

Internet-based Mindfulness Treatment

Archived at: http://www.webcitation.org/6mYjX09NU

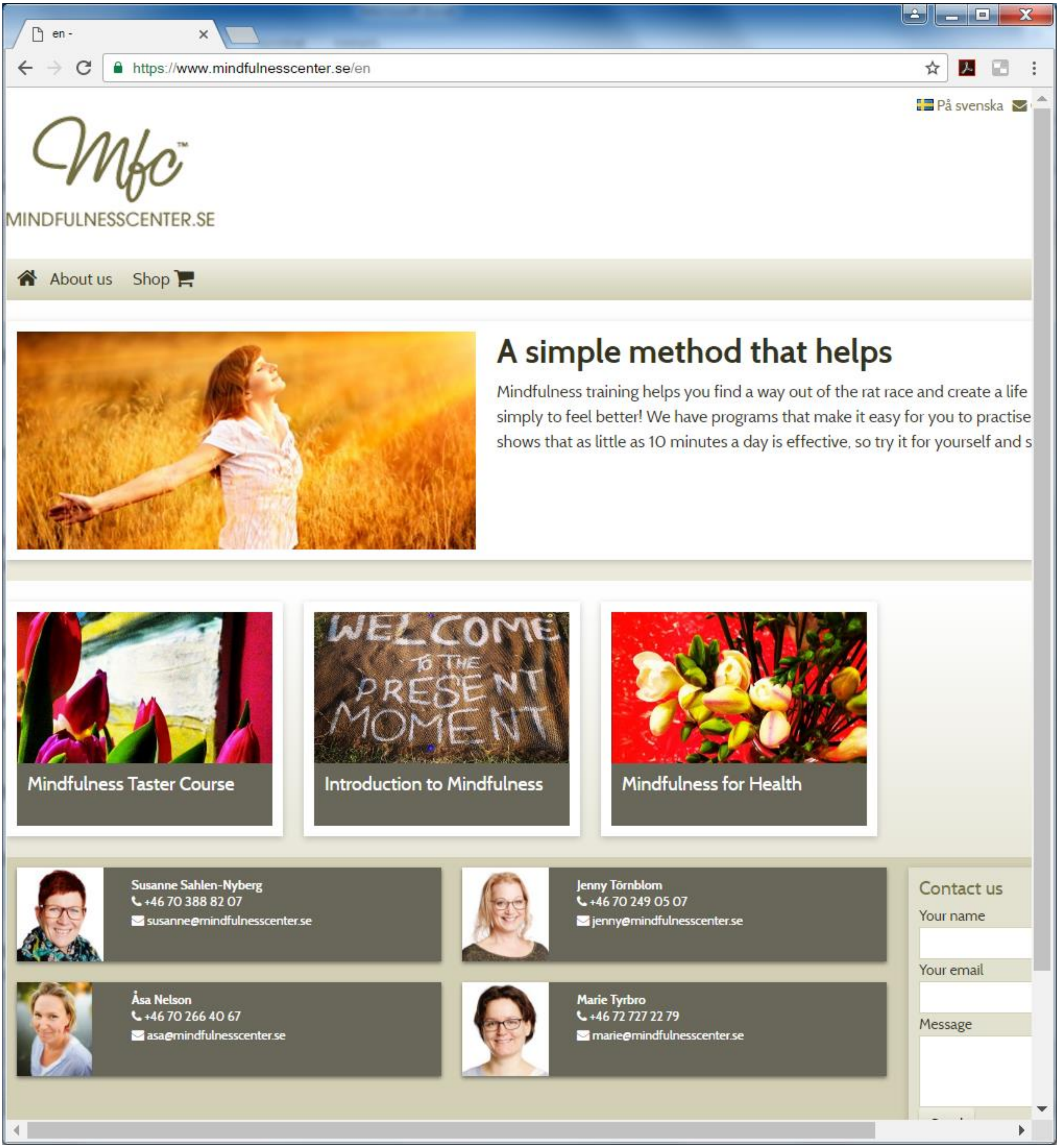

My Trauma Recovery

Archived at: http://www.webcitation.org/6mYjc236R

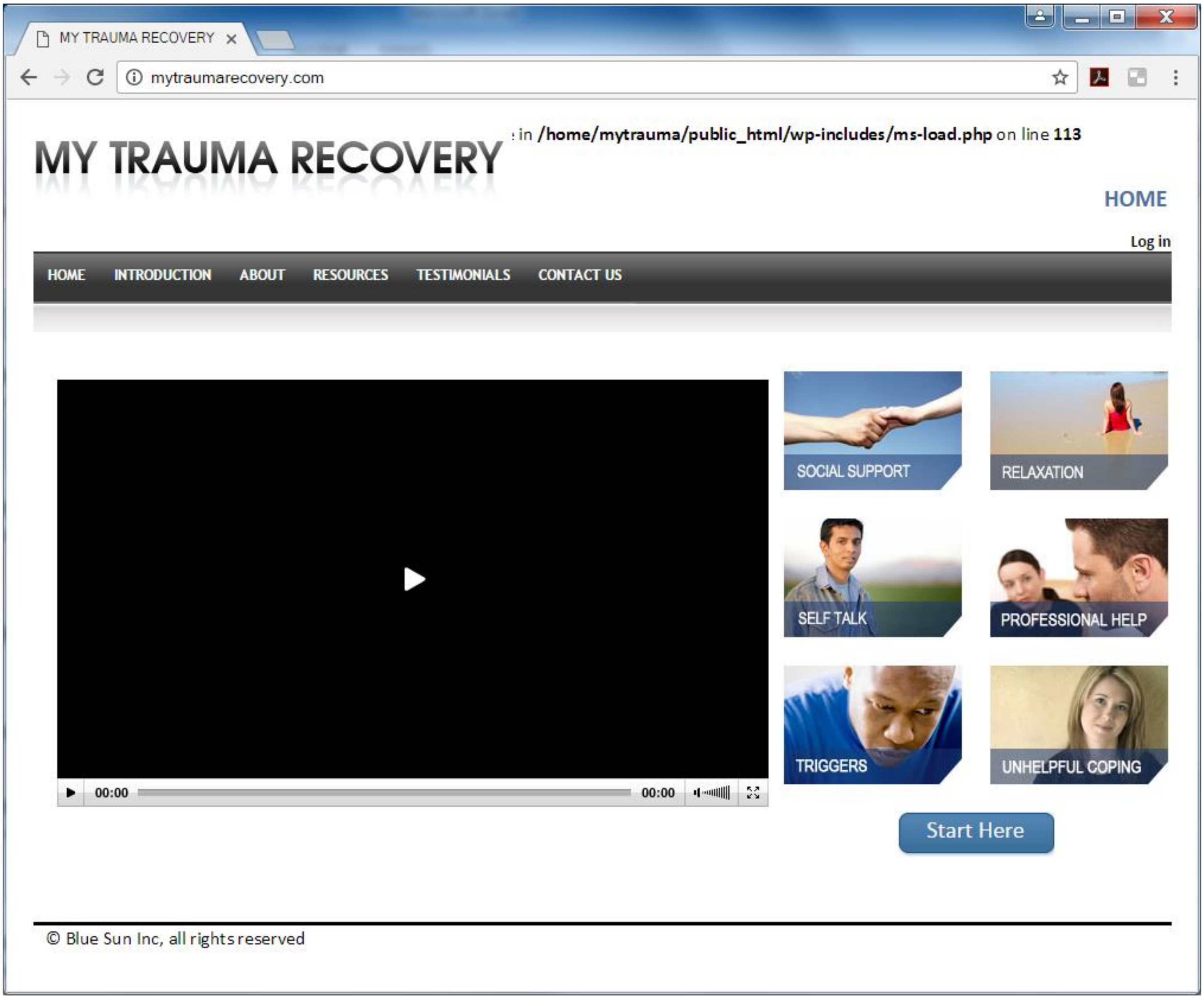

My Disaster Recovery

Archived at: http://www.webcitation.org/6mYjgJg0A

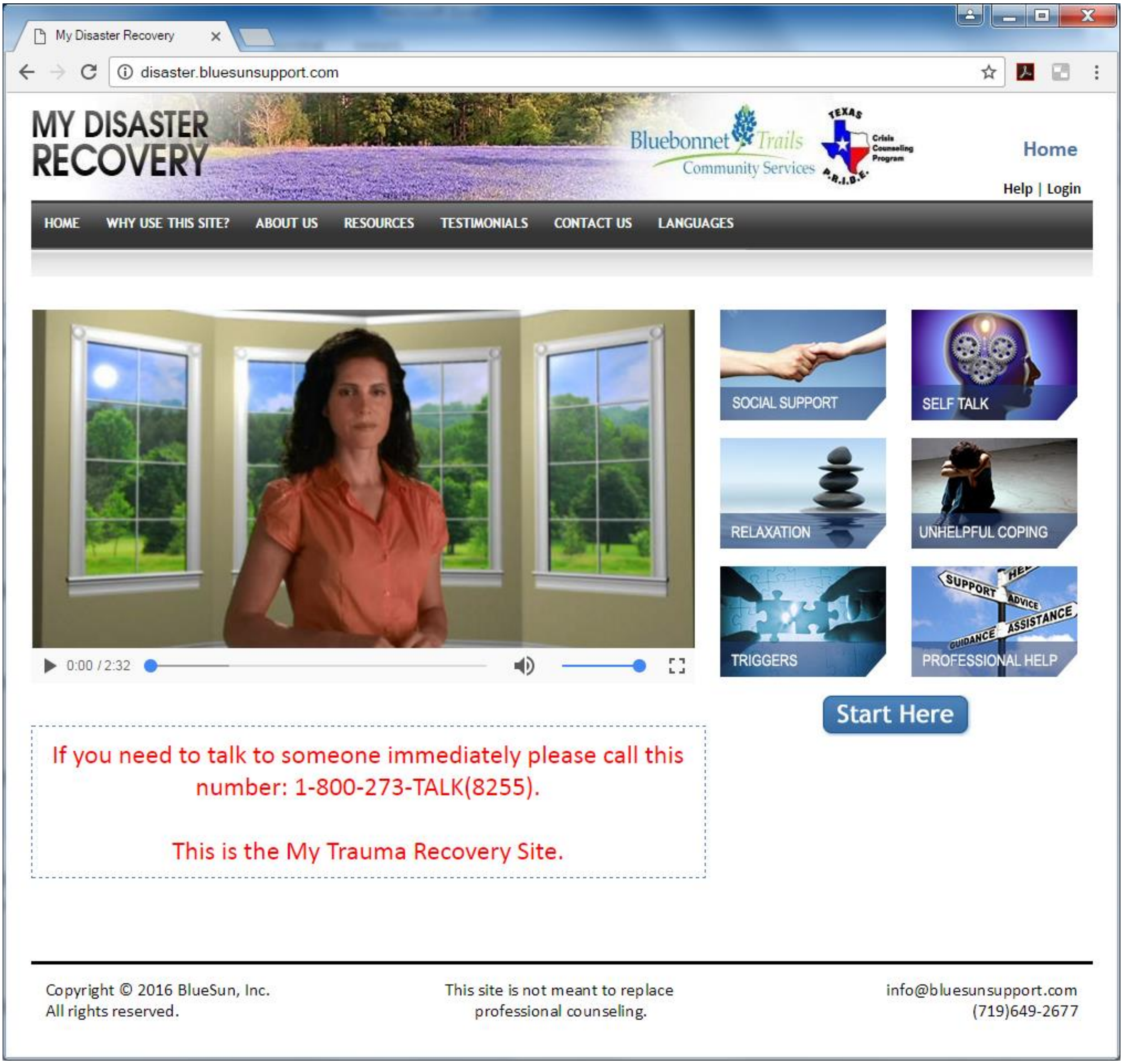

Talk to Me

Archived at: http://www.webcitation.org/6mYjlVGfO

Greetings from Dr. Net

www.internetmeayuda.com/mhpEnglish/saludo.htm

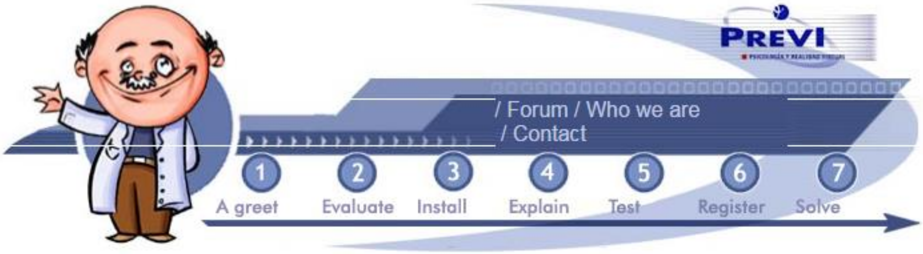

1. Greetings from Dr.Net!

Are you afraid of **giving a talk or a speech, taking an oral exam or presenting a project in front of a group of people?** Have you missed opportunities of promotion at work, or to obtain better grades in your studies for not having dared to speak in public?

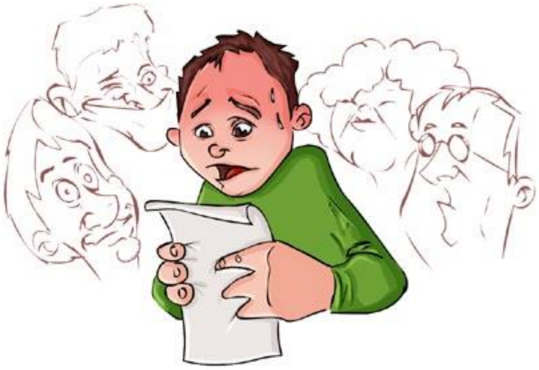

People with fear of public speaking, when they have to face these types of situations, experience great anxiety, they also tend to think that they are going to do it badly, and often think that their performance has been deficient. They think that others will notice that their voice or hands are shaking, or they believe that at any moment a great level of anxiety is going to invade them, or that they will not be able to correctly articulate words, etc...

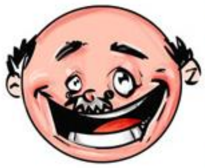

It is possible that you are reading these lines because you identified yourself with this situation. If this is the case, welcome, **you might find the solution to your problem here.** In order to do so, you must follow the steps that will guide you in how to do it.

Dr. Net will help you!

Healthy Living Check

Archived at: http://www.webcitation.org/6mYjqAbQS

Check. Leid je een gezond ×

www.gezondlevencheck.nl

Gezond leven check

Doe de gezondheidstests

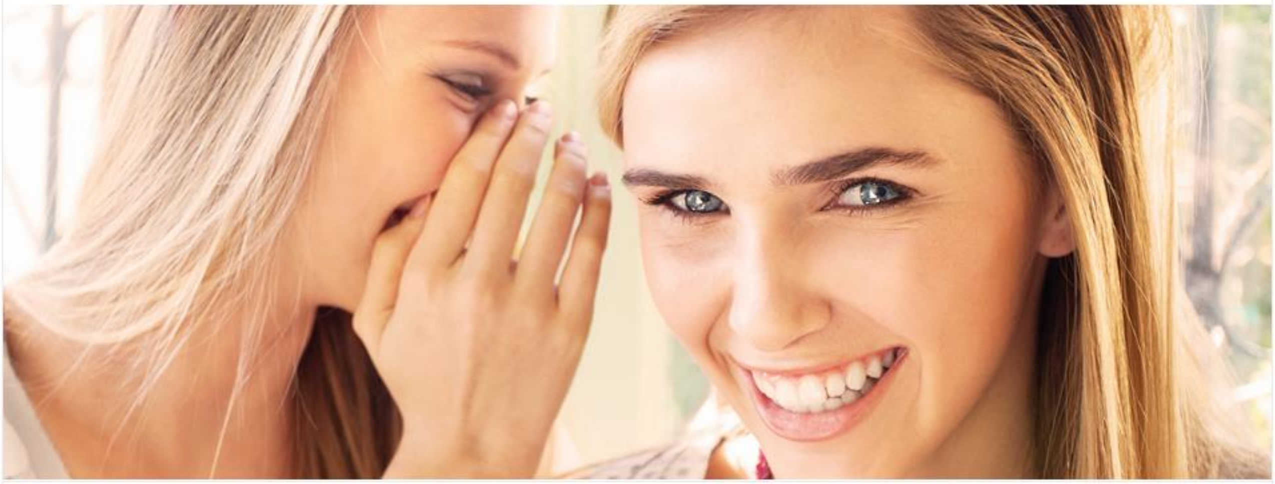

BMI check

Tweet

De BMI -check

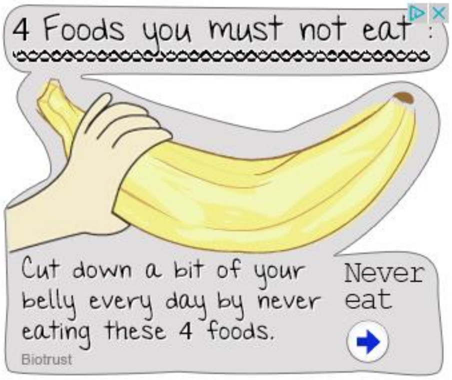

In relatie tot overgewicht, hoor je de laatste jaren vaak de term

Menu

- BMI check
- Leefstijl check
- Gezond eten check
  - Gezonde recepten
- Gehoor check
- Gezond bewegen check
- Huid check
- Ogen check
- Griep herkennen
- Contact
  - Sitemap
- En verder

Active Living Every Day

Archived at: http://www.webcitation.org/6mYjx8SFG

Active Living Every Day

www.activeliving.info/demo/demo_osg_welcome.cfm

ACTIVE LIVING partners

ACTIVE LIVING *Every Day*

ALED HOME

- Getting Started
- My Journal
- Weeks 1 – 5
- Weeks 6 – 10
- Weeks 11 – 15
- Weeks 16 – 20
- Sign Out

CLOSE NEXT

Active Living Every Day Online Study Guide!

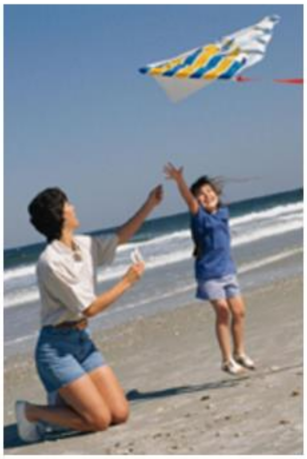

We at the Active Living Partners program have designed the *Active Living Every Day* course to help you become more physically active. As you work through the online study guide, you'll see that being more physically active may improve your health, give you more energy, and help you greet life with a brighter mental outlook.

The *Active Living Every Day* course takes 20 weeks to complete. Don't let the long timeline overwhelm you! We'll start out gradually and build new skills each week. We know you can do it because other people just like you have become more physically active by practicing the skills they learned in this course. By the end of our time together, you'll likely be up and moving more than before.

To read about the online study guide sessions, click on **Next.**

RETURN TO TOP CLOSE NEXT

My Path to Healthy Life

Archived at: http://www.webcitation.org/6mYk14Dw7

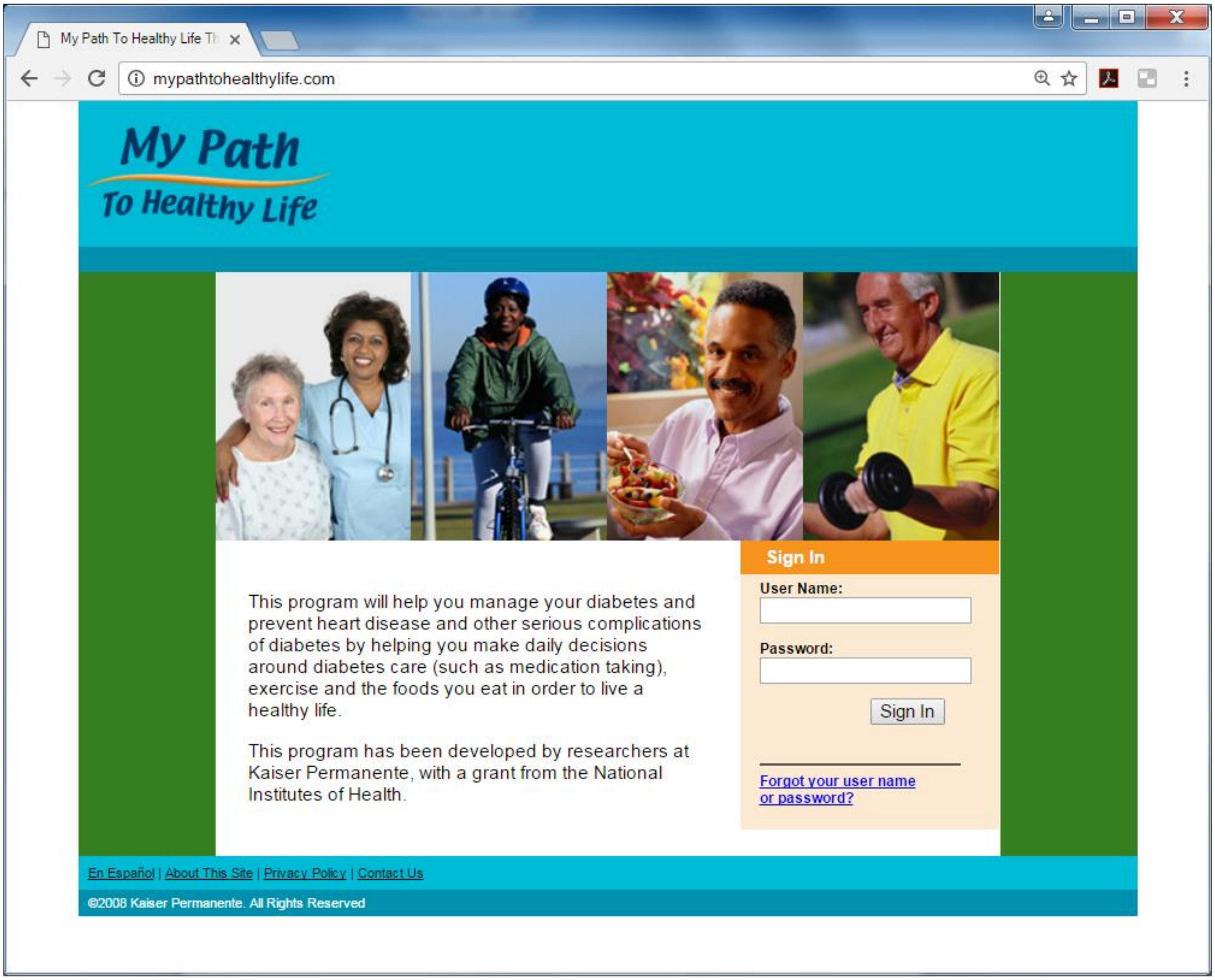

Food Smart

Archived at: http://www.webcitation.org/6mYk636L2

Food Smart - Center for W

centerforworkforcehealth.com/index.cfm/programs/food-smart/

Center for Workforce Health

Improving the Health and Productivity of Today's Workforce

home | programs | approach | who we are | outcome studies | clients & partners | contact

Our Programs

Back to Program List

Food Smart

Overview

Everyone wants to stay trim and eat a nutritious diet that will lead to a healthier, longer life. The *Food Smart* program, an interactive, multimedia program based on the latest approaches to weight management and nutrition, will enable users to analyze their eating habits, formulate an improved dietary plan, and engage in effective strategies for healthy weight management.

Approach

In the *Food Smart* program, science based information and strategies are presented to provide the user with the motivation and skills necessary to make lasting dietary changes. The purpose of this program is to assist the user in adopting a nutritious diet and managing weight, thereby *reducing the risk of major disease, including cancer, cardiovascular disease, and diabetes*. The program contains the following segments:

- Interactive exercises to help analyze current eating practices and choose new, more healthful practices.
- Learn about the Healthy Eating Plate from Harvard—a new guide to making smart food choices.
- See and hear though video testimonials from people who have successfully lost weight—and maintained a healthy weight.
- Strategies for food shopping, meal planning, and overcoming challenges.

The *Food Smart* program can be used alone or purchased as part of the *Health Connection* suite of programs that also includes *Active Lifestyle* and *Stress Management*.

Evidence

The NIH-supported randomized trial conducted at Ceridian [illegible]

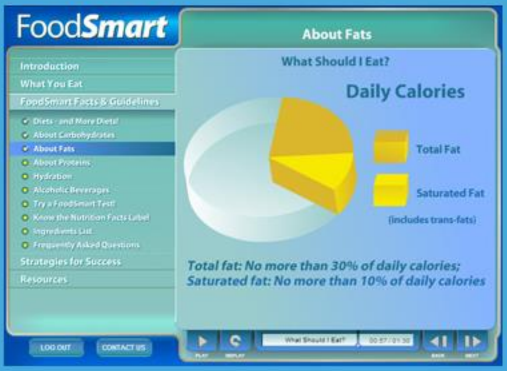

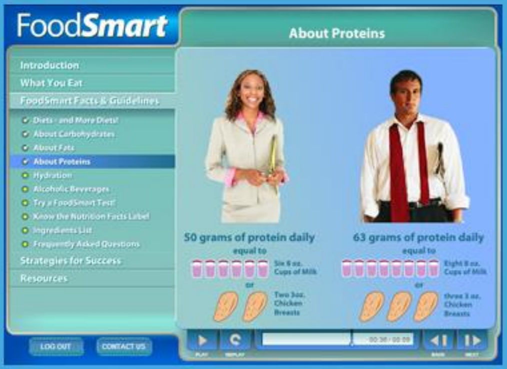

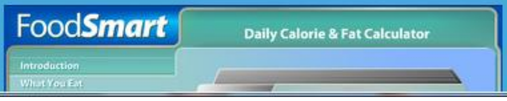

The Biggest Loser Club

Archived at: http://www.webcitation.org/6mYkAtZTq

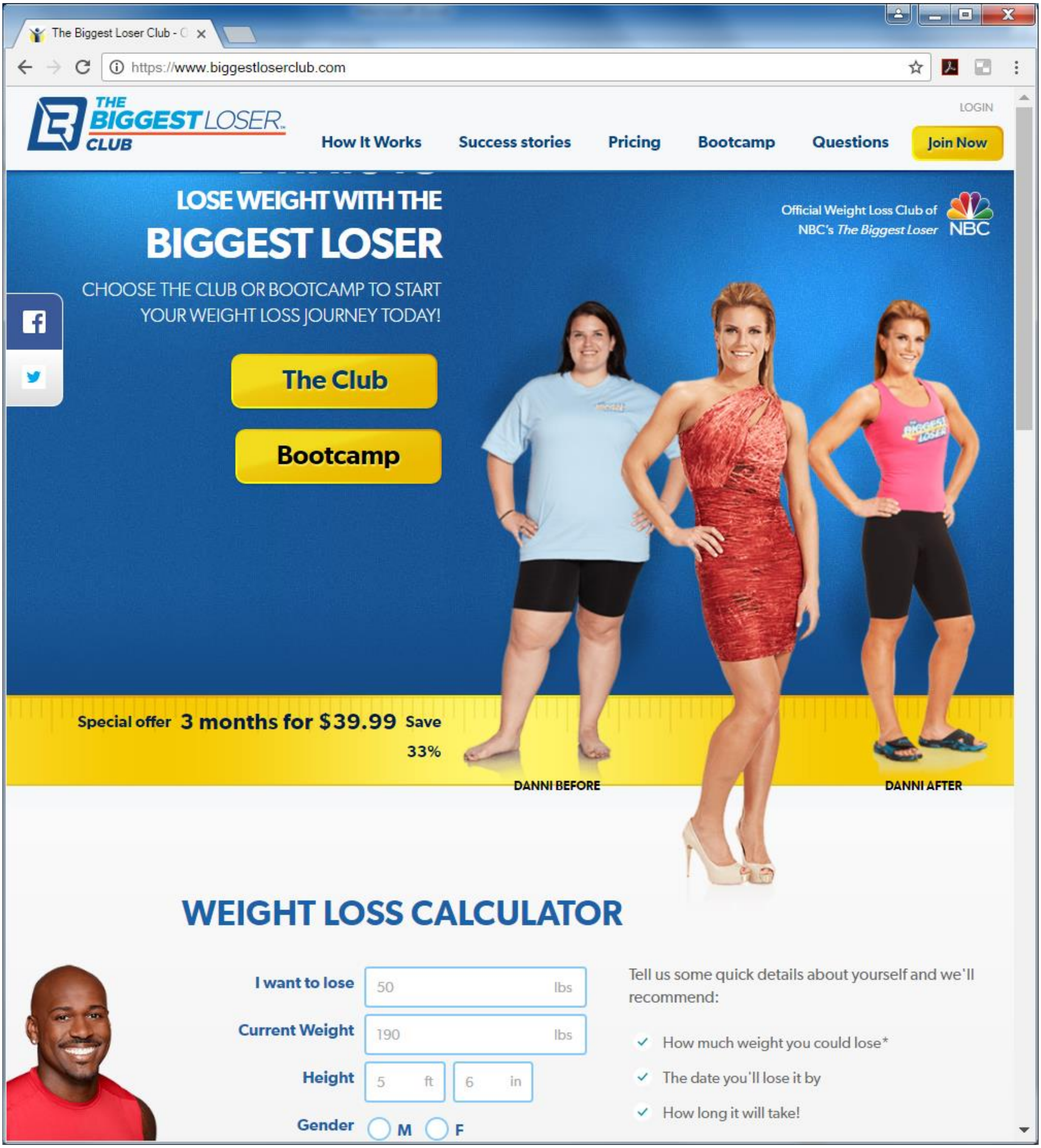

ExecuPrev

Archived at: http://www.webcitation.org/6mYkLJWdc

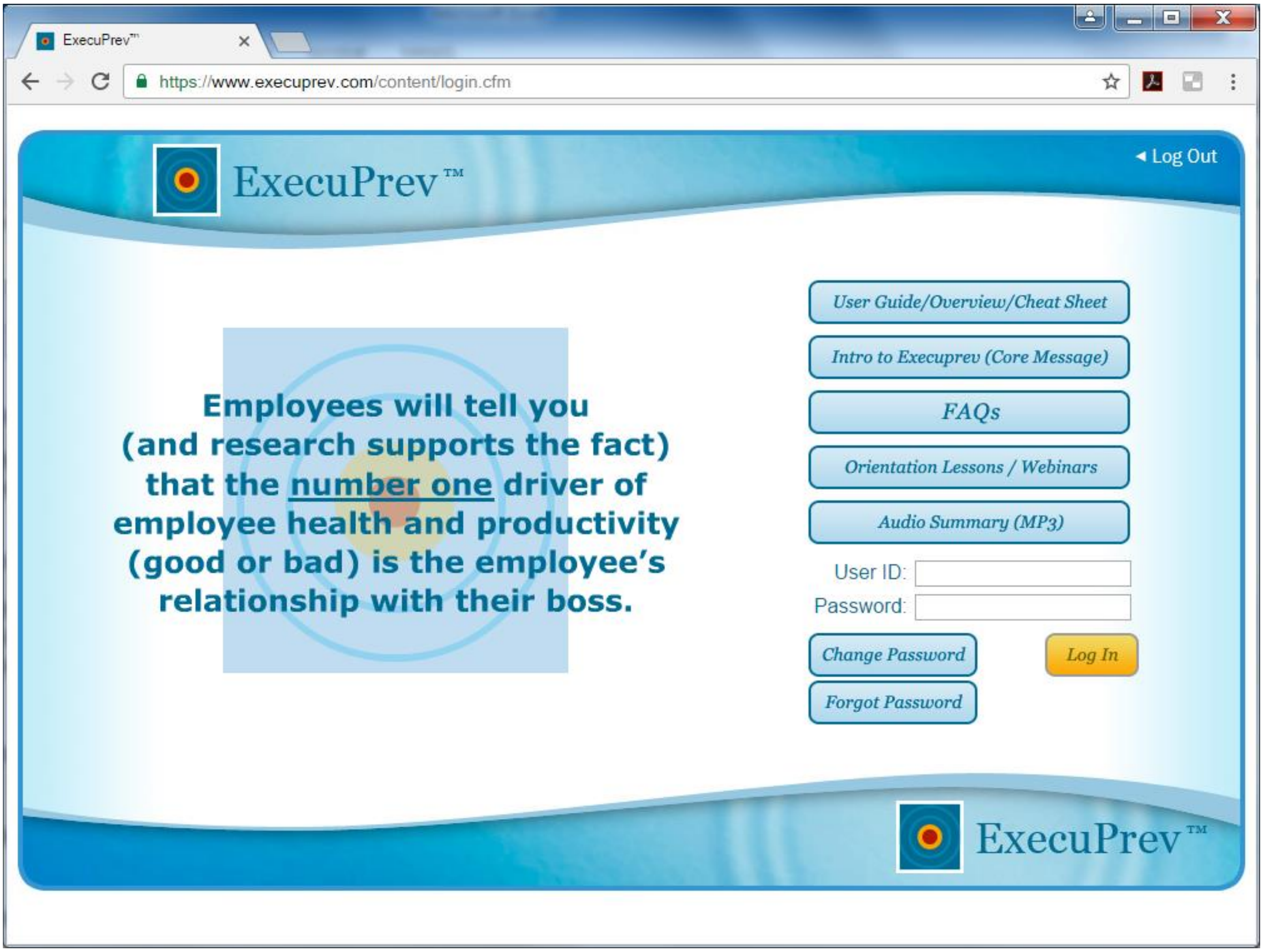

DASH for Health

Archived at: http://www.webcitation.org/6mYkVaUAM

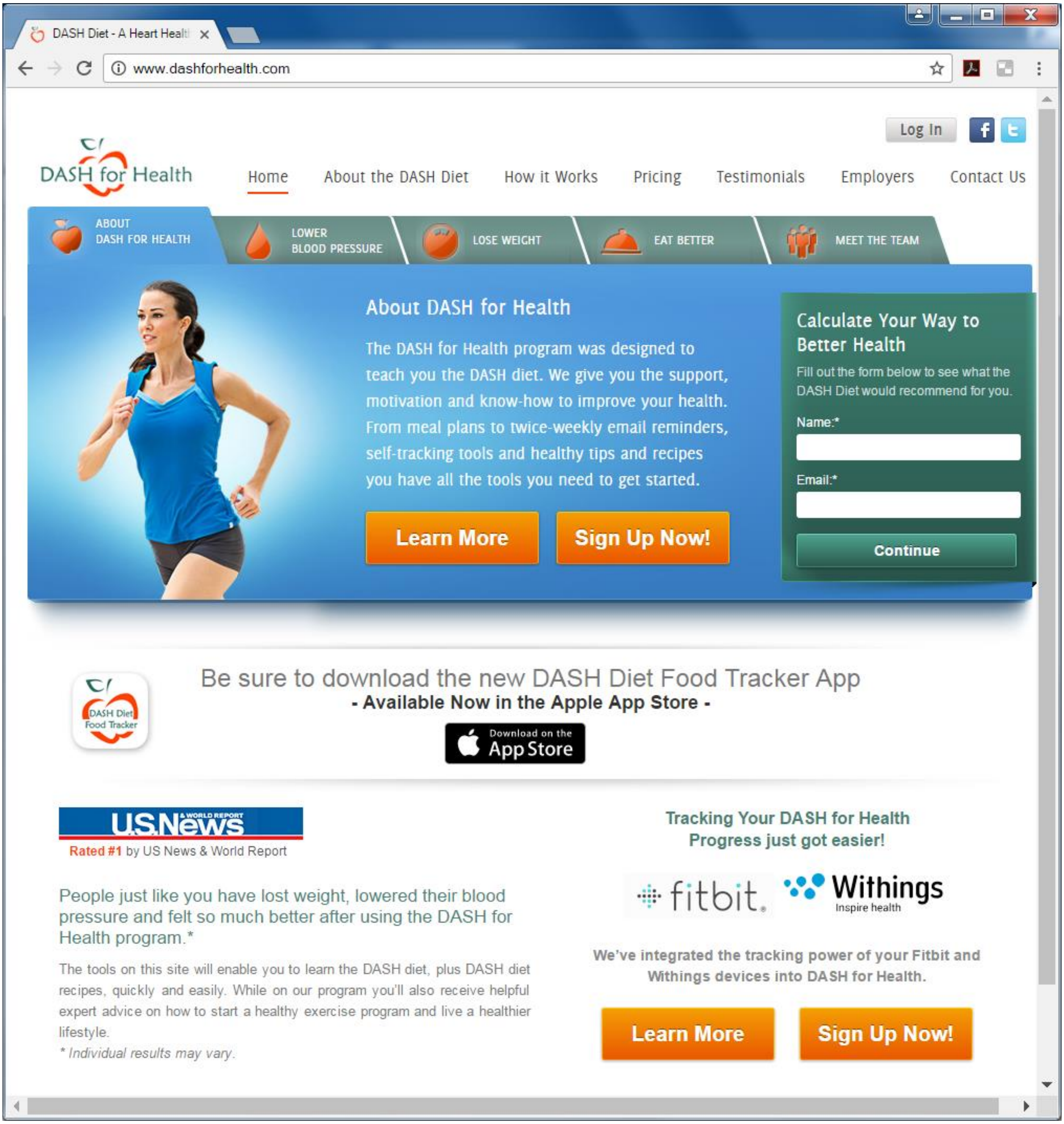

Insomnia

Archived at: http://www.webcitation.org/6mYkdB2ub

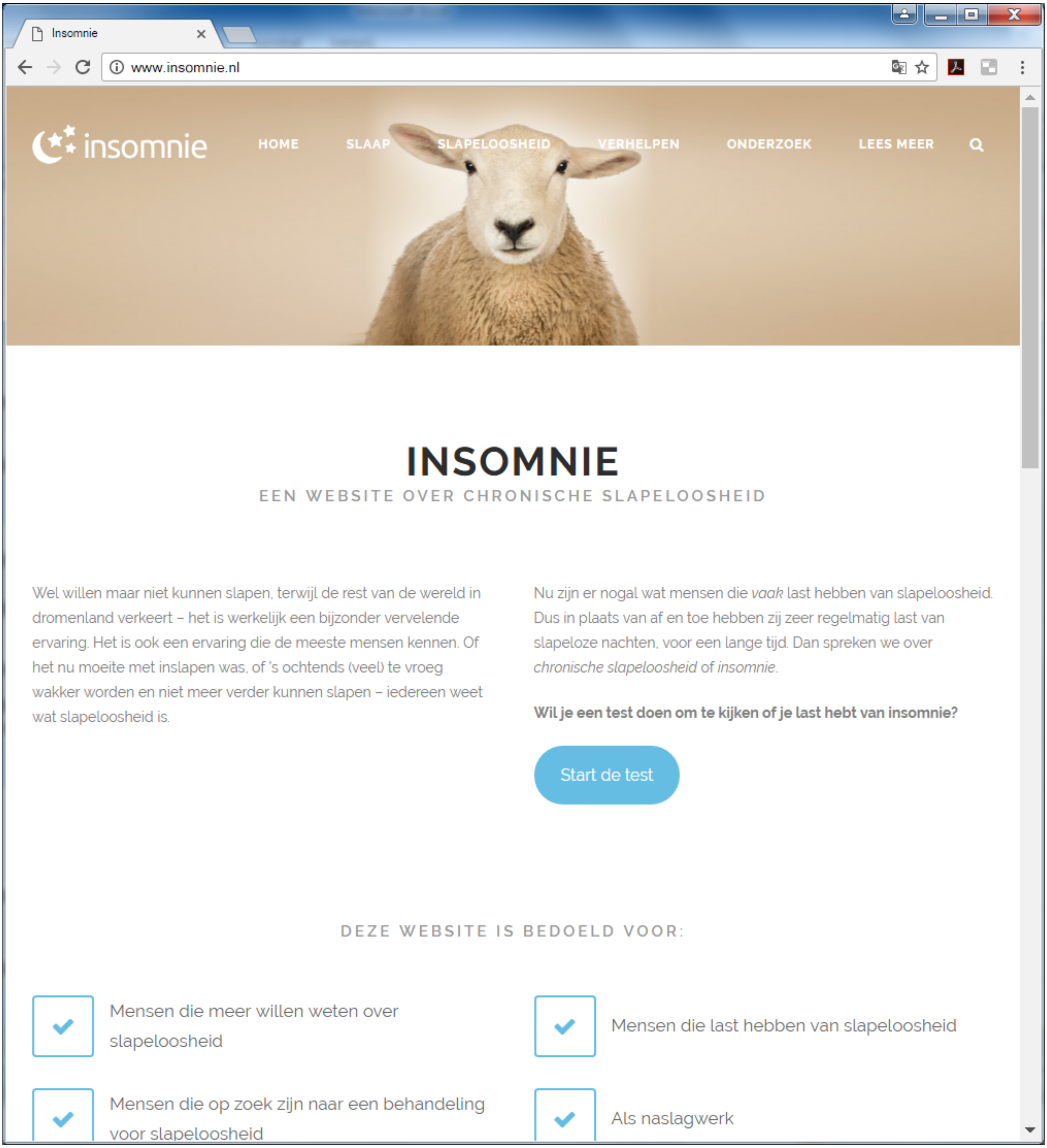

Insomnie

www.insomnie.nl

insomnie

HOME SLAAP SLAPELOOSHEID VERHELPEN ONDERZOEK LEES MEER

INSOMNIE

EEN WEBSITE OVER CHRONISCHE SLAPELOOSHEID

Wel willen maar niet kunnen slapen, terwijl de rest van de wereld in dromenland verkeert – het is werkelijk een bijzonder vervelende ervaring. Het is ook een ervaring die de meeste mensen kennen. Of het nu moeite met inslapen was, of 's ochtends (veel) te vroeg wakker worden en niet meer verder kunnen slapen – iedereen weet wat slapeloosheid is.

Nu zijn er nogal wat mensen die *vaak* last hebben van slapeloosheid. Dus in plaats van af en toe hebben zij zeer regelmatig last van slapeloze nachten, voor een lange tijd. Dan spreken we over *chronische slapeloosheid* of *insomnie*.

Wil je een test doen om te kijken of je last hebt van insomnie?

Start de test

DEZE WEBSITE IS BEDOELD VOOR:

- Mensen die meer willen weten over slapeloosheid
- Mensen die last hebben van slapeloosheid
- Mensen die op zoek zijn naar een behandeling voor slapeloosheid
- Als naslagwerk

SHUTi

Archived at: http://www.webcitation.org/6mYkl53g6

SHUTi | A proven online C[illegible]

www.myshuti.com

LOG IN

SHUTi™

Sleep Healthy Using the Internet

HOME | HOW SHUTi WORKS | PRICING | EVIDENCE | NEWS | FOR PROVIDERS | START TODAY

Insomnia is not something that needs to be endured

Referenced as “the best-studied program” by Harvard Health, SHUTi is designed to actively help retrain your body and mind for great sleep through six engaging Cognitive Behavioral Therapy for Insomnia strategy and learning sessions. Unlike printed material, video collections, or recorded lectures, each online Core is personalized to your current sleep patterns and goals, and walks you, step-by-step, through exactly what you need to do to maximum your sleep improvements, now and for the future.

SHUTi is an industry leading online CBT-I program with unsurpassed, proven results.

LEARN MORE

The American College of Physicians recommends that all adult patients with chronic insomnia receive cognitive behavioral therapy for insomnia, CBT-I, as their initial treatment.

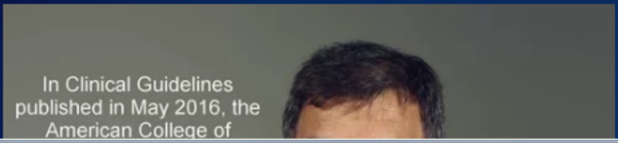

Do You Have Insomnia?

Sleepio

Archived at: http://www.webcitation.org/6mYksdTpQ

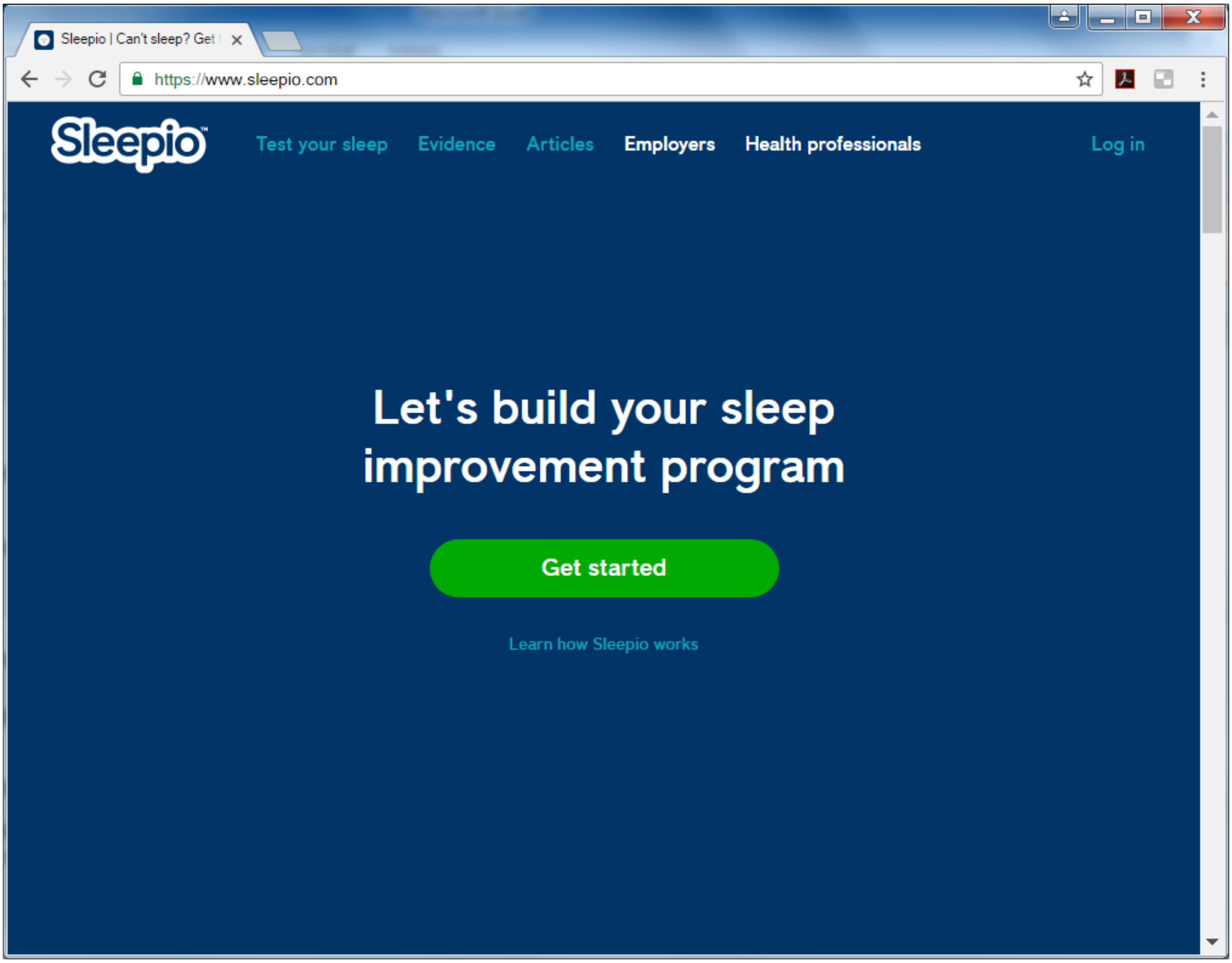

RESTORE

Archived at: http://www.webcitation.org/6mYl5apAA

Online CBT for Insomnia | ×

cobalttx.com/Products/restore.html

COBALT

Home About **Products** Partners Team Contact

Cobalt Therapeutics, LLC offers products that fit seamlessly into current clinical settings and enhance their therapeutic offerings to patients, decrease costs, and improve outcomes.

These programs have been evaluated in randomized, controlled trials and published in leading peer-reviewed journals.

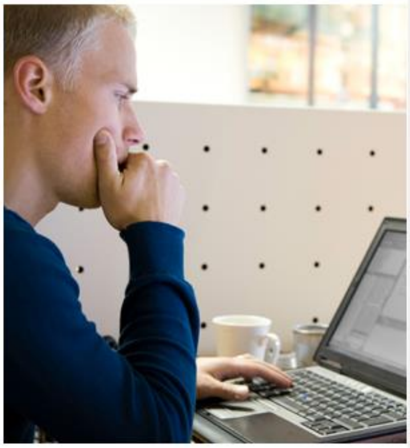

RESTORE™ CBT for Insomnia and sleep

RESTORE™ CBT for Insomnia and sleep problems, developed by Dr. Norah Vincent, received international attention when the data from its randomized, controlled trial was published in the Journal SLEEP in 2009. It was reported on in The New York Times, Time Magazine, US News, AOLHealth, and MSN. (The paper, by Dr. Vincent, was published in June 2009 and titled: "Logging on for Better Sleep: RCT of the Effectiveness of Online Treatment for Insomnia"). Cobalt is the sole provider of this program which showed:

- More than 80% improved
- Nearly an hour in additional time asleep
- Almost 40% decrease in time awake each night
- A 36% improvement in time taken to fall asleep
- A 20% improvement in work-related impairment due to insomnia

---

Awards and Press

painACTION

Archived at: http://www.webcitation.org/6mYl9lMxn

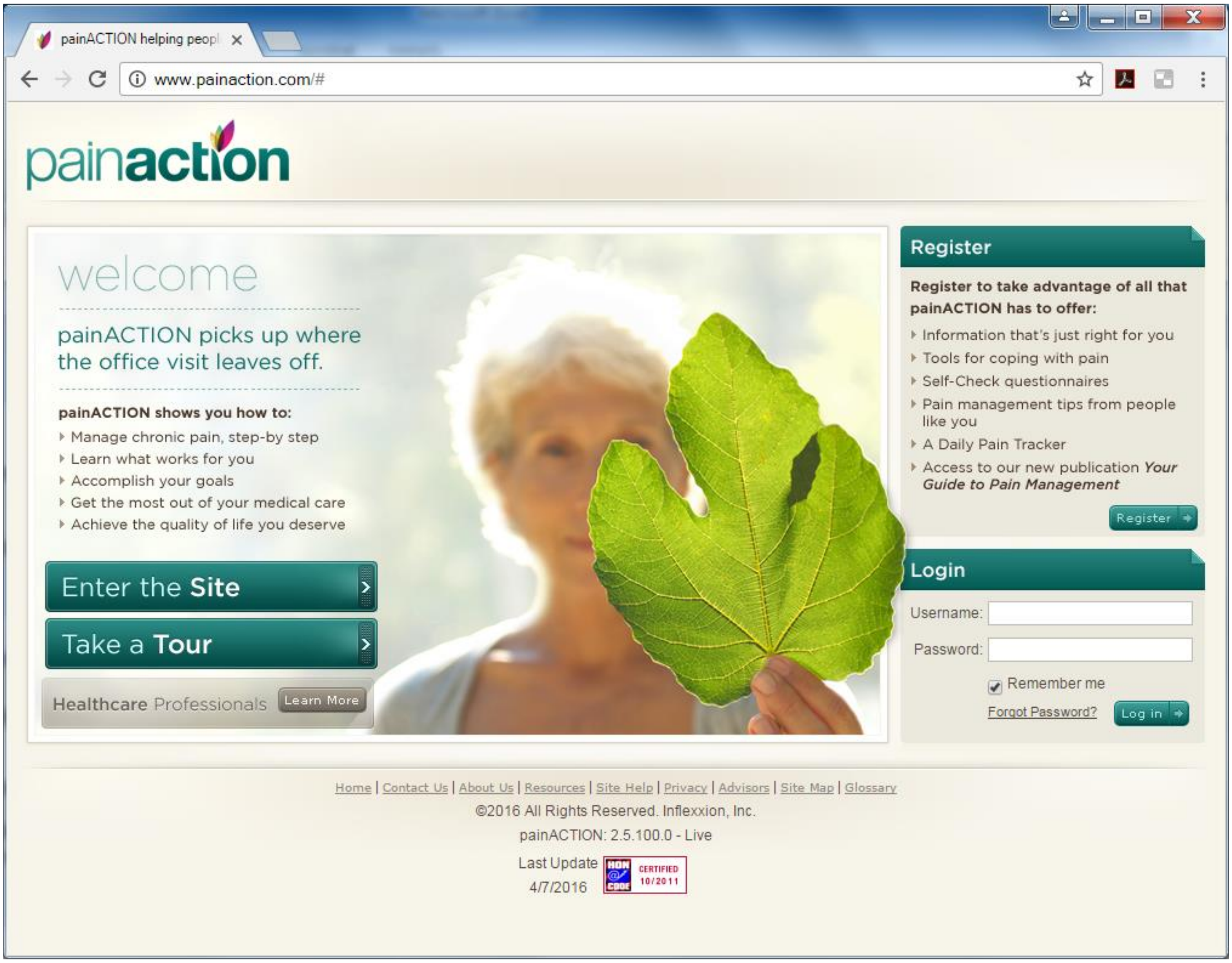

Chronic Pain Management Program

Archived at: http://www.webcitation.org/6mYlFlkZB

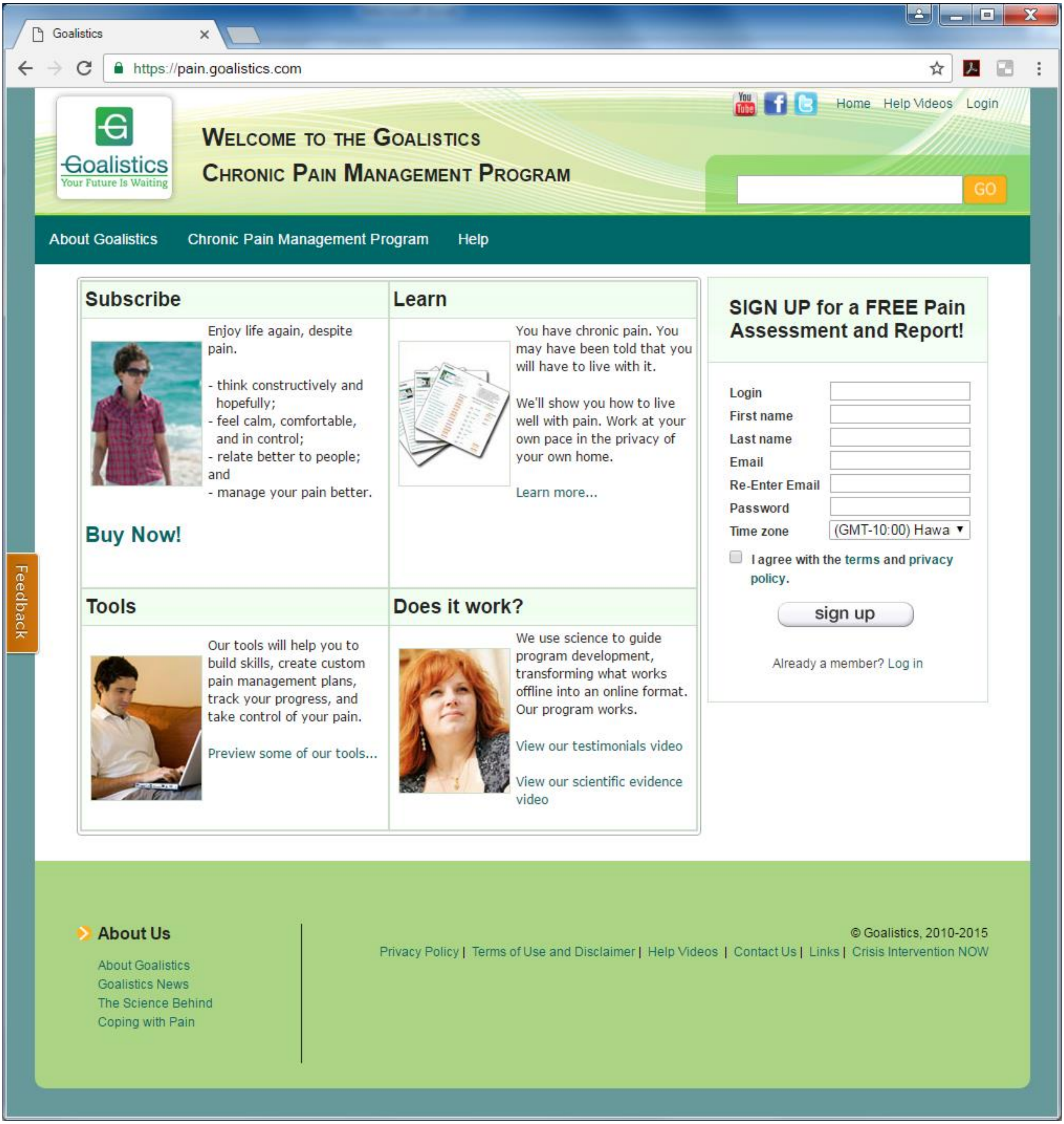

Goalistics

https://pain.goalistics.com

Goalistics
Your Future Is Waiting

Home Help Videos Login

WELCOME TO THE GOALISTICS
CHRONIC PAIN MANAGEMENT PROGRAM

GO

About Goalistics | Chronic Pain Management Program | Help

Subscribe

Enjoy life again, despite pain.

- think constructively and hopefully;
- feel calm, comfortable, and in control;
- relate better to people; and
- manage your pain better.

Buy Now!

Learn

You have chronic pain. You may have been told that you will have to live with it.

We'll show you how to live well with pain. Work at your own pace in the privacy of your own home.

Learn more...

Tools

Our tools will help you to build skills, create custom pain management plans, track your progress, and take control of your pain.

Preview some of our tools...

Does it work?

We use science to guide program development, transforming what works offline into an online format. Our program works.

View our testimonials video

View our scientific evidence video

SIGN UP for a FREE Pain Assessment and Report!

Login
First name
Last name
Email
Re-Enter Email
Password
Time zone (GMT-10:00) Hawa

I agree with the terms and privacy policy.

sign up

Already a member? Log in

Feedback

About Us

About Goalistics
Goalistics News
The Science Behind
Coping with Pain

Privacy Policy | Terms of Use and Disclaimer | Help Videos | Contact Us | Links | Crisis Intervention NOW

© Goalistics, 2010-2015

Blood Pressure Action Plan

Archived at: http://www.webcitation.org/6mYlM0hLf

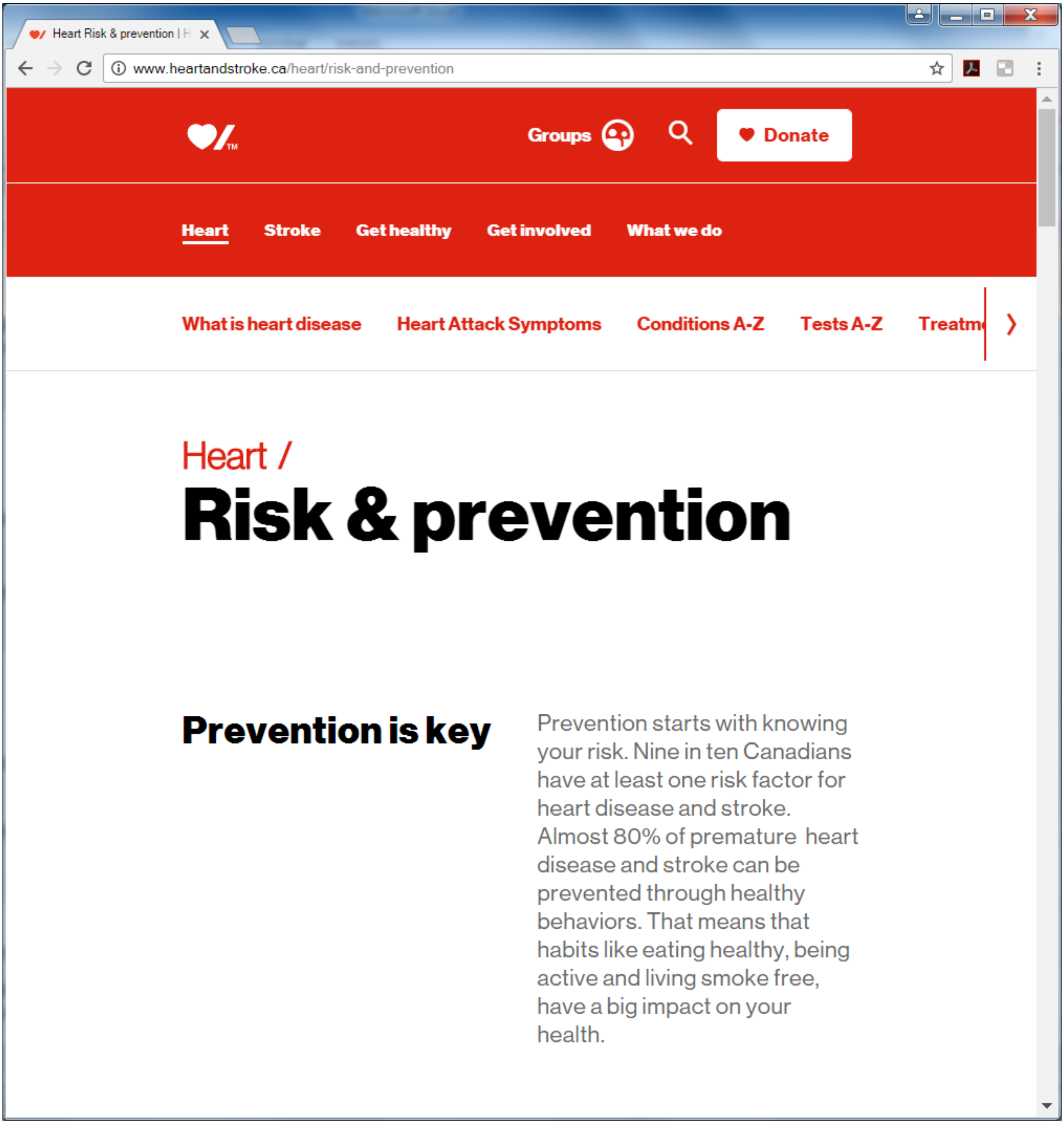

Heart Risk & prevention | H

www.heartandstroke.ca/heart/risk-and-prevention

Groups

Donate

Heart Stroke Get healthy Get involved What we do

What is heart disease Heart Attack Symptoms Conditions A-Z Tests A-Z Treatm

Heart /

Risk & prevention

Prevention is key

Prevention starts with knowing your risk. Nine in ten Canadians have at least one risk factor for heart disease and stroke. Almost 80% of premature heart disease and stroke can be prevented through healthy behaviors. That means that habits like eating healthy, being active and living smoke free, have a big impact on your health.

Triple P Online

Archived at: http://www.webcitation.org/6mYlQ50sy

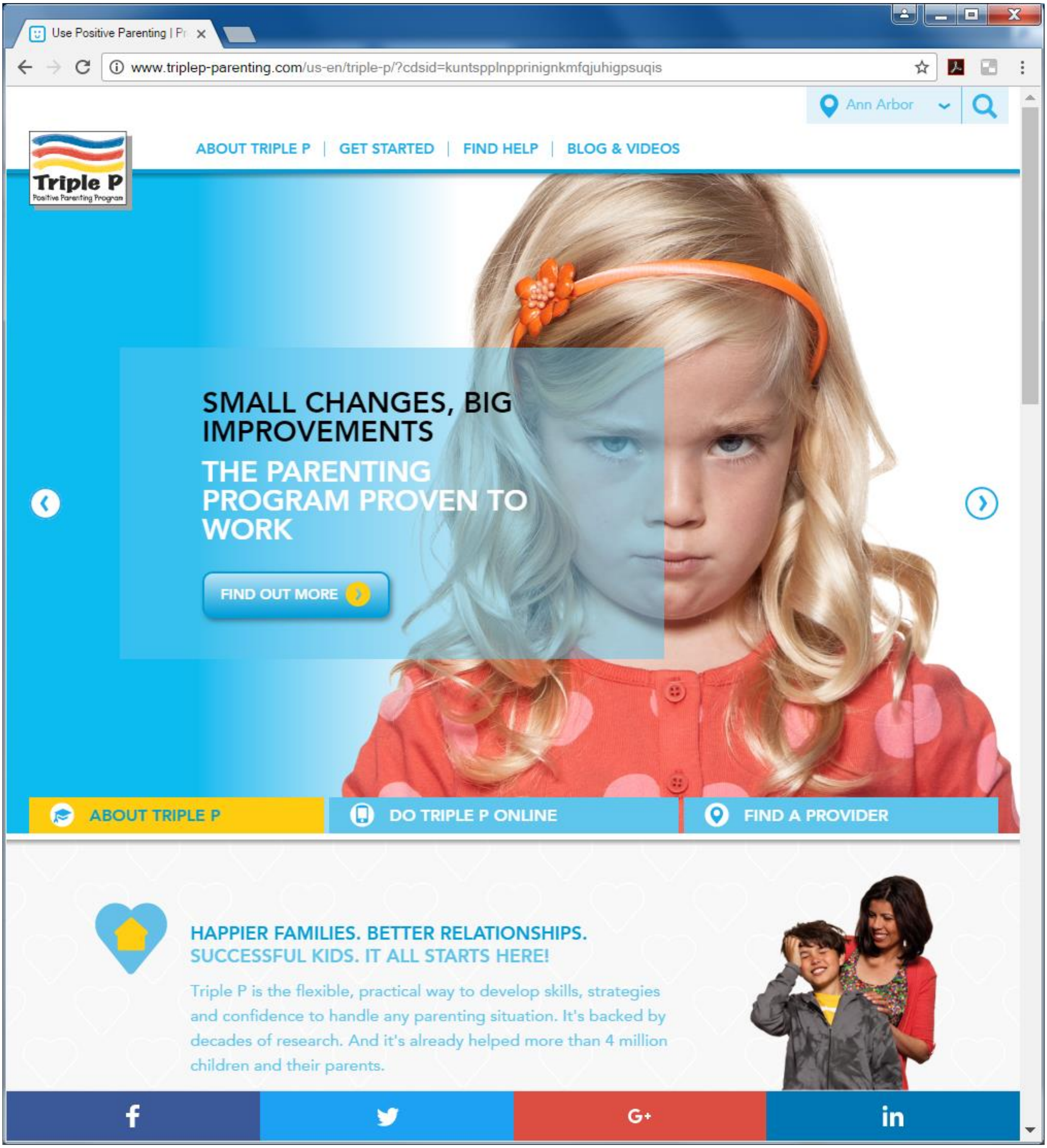

U Can POOP Too

Archived at: http://www.webcitation.org/6mYledqmJ

UCanPoopToo

www.ucanpooptoo.com

LOGIN

U Can POOP Too™

WHAT IS ENCOPRESIS? OUR COMPANY

What is Encopresis?

Home > What is Encopresis?

Search

About Encopresis

An estimated 2 million children in the United States have encopresis. When children have encopresis, this means that they cannot control their bowel movements and have poop accidents in their underwear. Sometimes people use the words "soiling" or "fecal incontinence" to mean encopresis.

Encopresis is a frustrating and difficult disorder for children and their families, and it can be hard to treat. In almost all cases, encopresis develops as a result of long-standing constipation. Most children suffering from encopresis have a history of constipation or passing very large, painful poops. In some cases, neither the child nor the parents recall the constipation since it was so long ago.

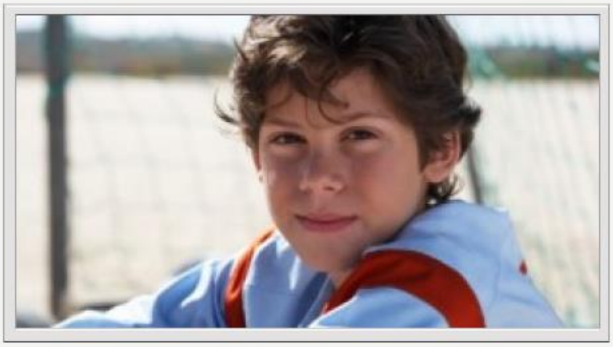

Encopresis is NOT your fault or your child's fault.

In a phone survey with parents of children with encopresis, ALL of the parents said they felt frustrated. When asked what information they would want to share with parents of children recently diagnosed with encopresis, their responses included:
